# Metabolic and Microbial Community Profiles of Century-Old Pu-Erh Tea: An Integrative Metabolomic and Microbiomic Analysis

**DOI:** 10.3390/foods15050916

**Published:** 2026-03-06

**Authors:** Huiqing Luo, Jianyang Fu, Yan Shen, Yuanfeng Chen, Siyu Zhou, Shikuan Zhao, Cheng Gong, Junlin Tai, Ya Wang, Wenshu Peng, Liang Yan, Chongye Fang, Ruijuan Yang

**Affiliations:** 1 College of Food Science and Technology, Yunnan Agricultural University, Kunming 650201, China; 18788107018@163.com (H.L.);; 2 College of Agriculture and Biotechnology, Yunnan Agricultural University, Kunming 650201, China; 3 Yunnan Pu’er Tea Factory Co., Ltd., Pu’er 665000, China; 4 Key Laboratory of Development and Utilization of Food and Medicinal Resources, Ministry of Education, Yunnan Agricultural University, Kunming 650201, China; 5 College of Tea (Pu’er), West Yunnan University of Applied Sciences, Pu’er 665000, China; 6 Pu’er Institute of Pu-erh Tea, Pu’er 665000, China

**Keywords:** Pu-erh tea, century-aged Pu-erh tea, non-volatile metabolites, metabolomics, microbiomics

## Abstract

As a uniquely Chinese post-fermented tea, Pu-erh tea undergoes profound changes in quality and flavor during aging, a process primarily driven by microbially mediated metabolic transformations. However, the patterns of microbe–metabolite co-evolution spanning a century-long timescale remain unclear. This study employed three samples—S (1920 raw Pu-erh tea), Y (1999 raw Pu-erh tea), and Q (2024 ripe Pu-erh tea)—integrating non-targeted metabolomics and microbiomics technologies to systematically analyze the characteristics of metabolites and microbial communities in century-old Pu-erh tea. The study elucidated the metabolic characteristics at the endpoint of long-term natural aging: the specific enrichment of hydrolyzable tannins, sucrose, and bipyrrole aromatic derivatives, providing a chemical basis for its unique “century-old charm”. Microbial community analysis indicated that long-term aging leads to simplified bacterial communities but increased fungal evenness, with the century-old sample specifically enriching for *Thermodesulfobacterium* and a large number of unclassified fungi. Multivariate statistics further constructed a microbe–metabolite interaction network, confirming significant correlations between key bacterial genera such as *Paenibacillus* and *Bacillus* and flavor precursors like sugars and phenolic acids.

## 1. Introduction

Pu-erh tea is a tea variety with distinctive quality characteristics made from sun-dried green tea leaves of the Yunnan large-leaf variety and processed through specific techniques within a geographically protected area. Based on processing methods and quality attributes, Pu-erh tea is categorized into raw Pu-erh tea and ripe Pu-erh tea [1]. Raw Pu-erh is produced by sun-drying and rolling fresh leaves without undergoing the pile-fermentation process; its intrinsic components then undergo slow yet profound changes through natural aging, influenced by the synergistic effects of microorganisms and environmental factors [2]. In contrast, ripe Pu-erh undergoes a crucial “pile-fermentation” step, where tea leaves experience a series of chemical and biological transformations under the combined effects of heat, high humidity, oxygen, and various enzymes, primarily through thermocatalytic and enzymatic reactions [3]. This process rapidly develops the characteristic dark red liquor, mellow taste, and distinctive “aged aroma” within months.

Numerous studies indicate that storage duration significantly impacts the quality attributes and microbial community structures of various teas, consequently altering their flavor [4,5]. The storage of Pu-erh tea is essentially a form of post-fermentation [6]. As storage time extends, the tea’s internal components undergo considerable changes under the combined influence of microorganisms, enzymes, heat, and humidity [7]. For example, during storage, microorganisms like Aspergillus [8], Saccharomyces [9], and Bacillus [10] grow, altering the microbial community structure. The secretion of extracellular enzymes by these organisms has been demonstrated to exert a differential effect on the content of theaflavins, theabrownins, tea polyphenols, water-soluble sugars, and gallic acid. Regarding aroma components, floral compounds tend to decrease, while pleasant woody, herbal, and medicinal notes characteristic of the “aged aroma” (Chen Xiang) significantly increase [11]. Therefore, the core of Pu-erh tea quality evolution lies in the microbial-driven fermentation process. This dynamic process is synergistically regulated by storage duration, environmental factors, and initial processing techniques.

In recent years, multi-omics technologies have provided novel perspectives for elucidating the aging mechanisms of Pu-erh tea. Metabolomics provides comprehensive profiling of all small-molecule metabolites in tea leaves under specific physiological conditions. Meanwhile, microbiomics reveals the structure and function of the microbial communities that play a crucial role in tea processing and aging. The integration of metabolomics with high-throughput sequencing technology has been demonstrated to facilitate analysis of the impact of storage duration on non-volatile metabolites and fungal communities in Liu Bao tea. This approach reveals dynamic associations between key fungal groups—including *Eurotium*, *Aspergillus*, and *Blastobotrys*—and the transformation of alkaloids, organic acids, terpenoids, and flavonoids during Liu Bao tea (LPT) storage [12]. Another study integrated metabolomics and microbiomics to analyze metabolic differences among oolong teas of varying grades and their microbial drivers. It identified six non-volatile compounds and 22 volatile compounds as key quality-determining substances in oolong tea. Significant microbial differences were observed in *Paenibacillus*, *Haemophilus*, among others. Microorganisms such as *Prevotella*, *Schaalia*, and *Niallia* were positively correlated with oolong tea quality [13]. Furthermore, using metabolomics and microbiomics, another study investigated the patterns of flavonoid and amino acid changes in white tea across different storage years and further analyzed the correlations between these metabolic shifts and microbial community structures [14].

However, existing research has primarily focused on analyzing volatile metabolites in Pu-erh tea aged for specific periods ranging from several years to several decades [15,16,17]. Multi-omics studies on century-old teas aged over 80 years remain scarce. This study innovatively integrates samples spanning a century-long gradient: S (1920 raw Pu-erh tea), Y (1999 raw Pu-erh tea), and Q (2024 ripe Pu-erh tea). Through non-targeted metabolomics and microbiomics technologies, we conduct in-depth analyses of these three representative samples. Through this design, we aim to achieve the following objectives: (a) to descriptively compare the metabolome and microbiome profiles of Pu-erh tea samples with distinct aging histories and processing types (1920 raw, 1999 raw, and 2024 ripe Pu-erh); (b) to preliminarily screen for key metabolites and microbial taxa that are relatively enriched in the endpoint of long-term natural aging; and (c) to construct a microbe–metabolite interaction network based on statistical correlations, aiming to generate hypotheses regarding the potential functional roles of microbes in shaping Pu-erh tea flavor and quality, thereby providing directions for future mechanistic studies.

## 2. Materials and Methods

### 2.1. Materials

All the Pu-erh tea samples used in this study were provided by Yunnan Pu’er Tea Factory Co., Ltd., and certified as genuine (Pu’er City, Yunnan Province, China). The raw materials consist of sun-dried green tea made from Yunnan large-leaf varietal tea leaves. The samples encompass Pu-erh teas from three distinct aging periods: S (1920 raw Pu-erh tea), Y (1999 raw Pu-erh tea) and Q (2024 ripe Pu-erh tea). Prior to collection, all samples were stored under the factory’s standard warehouse conditions at a temperature of 20–35 °C and a relative humidity of 60–80%. Before analysis, tea experts verified the samples’ authenticity and aging history through production records, packaging labels and sensory evaluation. The samples were then immediately placed in sterile, sealed, light-protected, food-grade aluminum foil bags and transported to the laboratory. In the laboratory, the samples were stored under dry, well-ventilated, constant temperature conditions (25 ± 2 °C). All pretreatment and analytical experiments were completed within two weeks of collection to minimize the impact on the samples’ original metabolic and microbial states. To ensure analytical consistency and comparability, three biological replicates were established for each vintage. Each replicate was sourced from a different part of the same tea batch, ground aseptically, mixed, passed through a 60-mesh sieve, portioned uniformly, and stored at −80 °C until metabolite and microbial DNA extraction.

### 2.2. Determination of Metabolites

The experiment employed ultra-high-performance liquid chromatography coupled with high-resolution mass spectrometry (UPLC-MS/MS; Thermo Q Exactive Focus, Thermo Fisher Scientific, Waltham, MA, USA) for detection. Metabolite extraction: Following the sample preparation method [18], tea liquor was centrifuged at 4 °C and 12,000 rpm for 10 min. One milliliter of supernatant was collected, vacuum-concentrated, and redissolved in 70% methanol. The solution was then filtered through a 0.22 µm PTFE microporous membrane and the filtrate was collected in vials for LC-MS analysis [19]. Three replicate samples were prepared for each treatment group (S, Y, Q). Each replicate was independently prepared and extracted, with three injections (technical replicates) to ensure analytical precision. During the LC-MS run sequence, blank samples (pure solvent) were injected at the start and after every six samples to monitor background signals. Quality control (QC) samples were prepared by pooling equal volumes of all sample extracts. These were injected at the start of the sequence, after every ten samples, and at the end of the sequence to evaluate instrument stability and repeatability during operation, and to provide a reference for subsequent data processing (e.g., signal drift assessment if necessary). The injection order was randomized to minimize batch effects. Separation was performed using a Thermo Vanquish UPLC system (Thermo Fisher Scientific, USA) equipped with an Acquity UPLC^®^ HSS T3 column (2.1 × 100 mm, 1.8 µm, Waters, Milford, MA, USA). The flow rate was 0.3 mL/min, the column temperature was 40 °C, the injection volume was 2 µL, and the mass spectrometer operated in both positive and negative ion modes. For positive ion mode, the mobile phase consisted of acetonitrile with 0.1% formic acid (B2) and water with 0.1% formic acid (A2). For negative ion mode, the mobile phase comprised acetonitrile (B3) and water with 5 mM ammonium formate (A3) [20]. Full MS scans were acquired at a resolution of 70,000 over a mass range of *m*/*z* 100–1000. [21].

### 2.3. An Analysis of DNA Extraction and Amplification

Total genomic DNA was extracted from the tea samples using the PowerSoil^®^ DNA Isolation Kit (QIAGEN, Germantown, MD, USA) in accordance with the manufacturer’s instructions. DNA quality and integrity were checked by 1% agarose gel electrophoresis. Using the extracted total DNA as a template, target gene fragments were amplified. The primers for bacterial 16S rRNA gene amplification were Bac 27f (5′-AGAGTTTGATCCTGGCTCAG-3′) and Bac 1492r (5′-ACGGCTACCTTGTTACGACTT-3′). The primers for fungal ITS region amplification were ITS1F (5′-CTTGGTCATTTAGAGGAAGTAA-3′) and ITS2 (5′-GCTGCGTTCTTCATCGATGC-3′). The PCR reaction system and conditions followed the method described by Yu et al. [22]. The purified PCR products were then employed in the construction of DNA libraries, which were subsequently dispatched to Suzhou PANOMICS Biomedical Technology Co., Ltd. (Suzhou, China), for high-throughput sequencing.

### 2.4. Metabolomics Data Processing and Analysis

Raw mass spectrometry data were processed using ProteoWizard software (v3.0.8789) [23] and the XCMS package in R for baseline filtering, peak identification, peak alignment, and retention time correction. Parameter settings: bw = 2, ppm = 15, peakwidth = c(5, 30), mzwid = 0.015, mzdiff = 0.01, method = “centWave”, yielding a quantitative list of substances. Data correction was performed using total peak area normalization to eliminate systematic errors. Multivariate statistical analysis, incorporating both unsupervised Principal Component Analysis (PCA) and supervised Orthogonal Projections to Latent Structures—Discriminant Analysis (OPLS-DA), was conducted utilizing the Ropls package in R (v 4.2.3) [24]. Differentially expressed metabolites (DEMs) were identified based on variable importance in projection (VIP) values and statistical significance [25]. Metabolite identification was performed by searching and matching against spectral databases, including HMDB [26], MassBank [27], LipidMaps [28], mzCloud [29], KEGG [30], and a self-built metabolite standard database from PANOMICS. Metabolites with MS/MS spectra in the quantitative list were compared and matched against the database fragment ion information to achieve secondary identification. KEGG pathway analysis was used to analyze the differential metabolites’ metabolic pathways.

### 2.5. High-Throughput Sequencing Analysis

In order to ensure the integrity of the sequence data, the DADA2 pipeline (version 2019.4) from QIIME2 was utilized for the following purposes: sequence quality control, denoising, merging, and chimera removal. Analyzes including OTU clustering, taxonomic classification, alpha diversity, and beta diversity were performed using QIIME2 (2019.4), R language, and the ggplot2 package, among others. Bacterial 16S rRNA gene sequences were annotated and classified using the SILVA database [31]. Fungal ITS sequences were annotated and classified using the UNITE database (version 10.0) [32].

### 2.6. Correlation Analysis

Pearson correlation analysis was employed to elucidate the potential associations between key differential metabolites and the structure of microbial communities. A sophisticated correlation analysis was conducted using the OmicStudio tools, which can be accessed at https://www.omicstudio.cn/tool.

## 3. Results and Discussion

### 3.1. Metabolomic Characteristics of Pu-Erh Tea Across Different Aging Years

**Figure 1:**
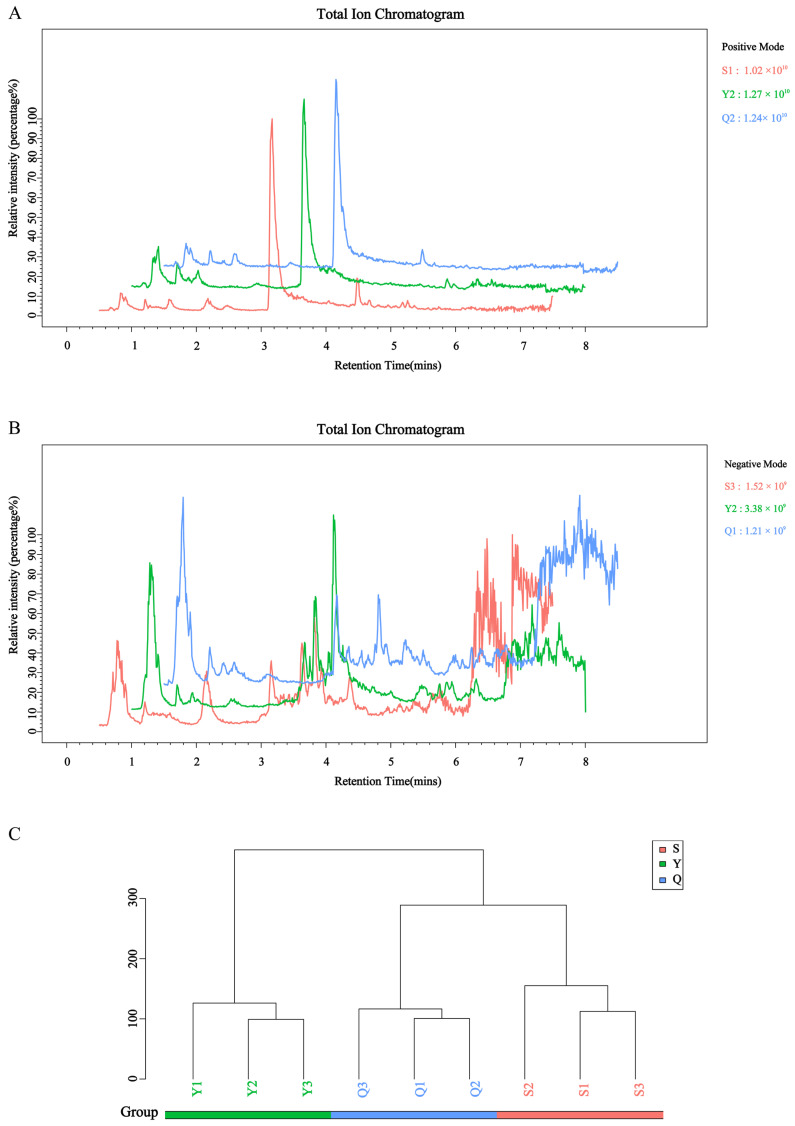
Analysis of metabolomics in Pu-erh teas of different ages. (**A**) Total ion chromatogram (TIC) of samples in positive ion mode. (**B**) TIC of samples in negative ion mode. (The *x*-axis represents the retention time, and the *y*-axis represents the ion intensity. The value in the upper-right corner of each panel indicates the maximum ion intensity for the corresponding sample. Different colors represent different groups.) (**C**) Hierarchical clustering tree of samples.

The present study employed a systematic approach to analyze the chemical profiles of three samples using UPLC-MS/MS-based untargeted metabolomics in order to decipher the systemic effects of aging times on the Pu-erh tea metabolome. Total ion chromatograms (TIC) (Figure 1A,B) demonstrated highly consistent retention times and peak intensities for secondary metabolites across samples in both positive and negative ion modes, indicating excellent instrument stability and reproducibility of sample preparation. After peak extraction, alignment, and calibration, 18,407 metabolite ion features were captured (8244 in positive mode; 10,163 in negative mode). Matching against standard databases resulted in the annotation of 1825 secondary metabolites (Table S1), including 337 lipids and lipid-like molecules, 183 organic acids and derivatives, 168 organoheterocyclic compounds, 165 benzene derivatives, 135 phenylpropanoids and polyketides, 125 organic oxygen compounds, 28 nucleosides, nucleotides, and analogs, 19 organic nitrogen compounds, 13 alkaloids and derivatives, 7 hydrocarbons, 5 lignans, neolignans, and related compounds, 4 organosulfur compounds, 2 homogeneous non-metal compounds, 2 organophosphorus compounds, 1 homogeneous metal compound, 1 organometallic compound, and 630 other compounds, highlighting the high complexity of the Pu-erh tea metabolome.

Hierarchical cluster analysis based on Euclidean distance revealed differences in metabolic profiles between samples (Figure 1C). The S, Y, and Q groups each formed distinct branches; the S and Q samples clustered together first, then merged with the Y sample. This indicates that intra-group differences within the three Pu-erh tea groups are smaller than inter-group differences, yet there is still some association between groups, with S and Q exhibiting higher similarity. Previous studies suggest that inoculating fermentation with selected dominant strains can shorten the cycle, optimize quality, and rapidly produce products with physicochemical components similar to aged Pu-erh tea [33]. The ripe Pu-erh tea process successfully simulates several key outcomes of natural aging and efficiently replicates certain characteristics of natural aging in major chemical indicators and overall taste, representing an effective technical path for the rapid “maturation” of Pu-erh tea flavor. However, it cannot fully replicate the delicate, rich, and complex flavor layers and “aged aroma” formed by a century of natural aging.

### 3.2. Screening of Differentially Expressed Metabolites

**Figure 2:**
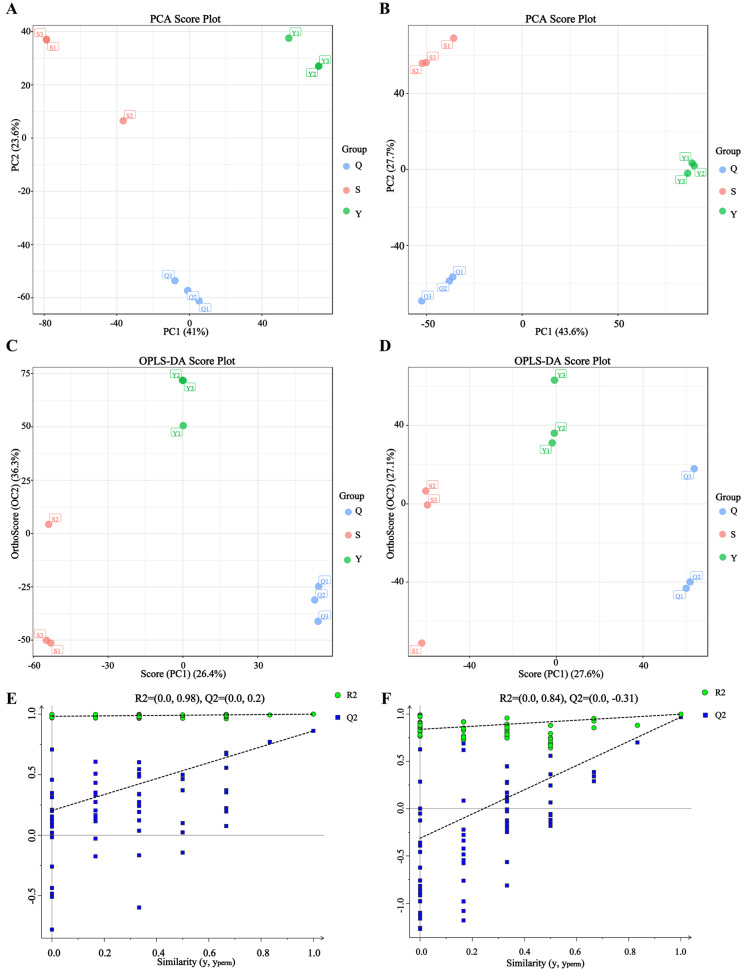
PCA and OPLS-DA of Pu-erh tea samples from different years. (**A**,**B**) PCA score plots obtained in positive and negative ion modes, respectively. (**C**,**D**) OPLS-DA score plots derived from positive and negative ion modes, respectively. (**E**,**F**) Permutation test results for validating the corresponding OPLS-DA models.

The investigation of the variances present within the metabolite categories of the three tea samples was facilitated through the execution of a Principal Component Analysis (PCA) procedure for each Pu-erh tea category under consideration, employing both positive and negative ion modes (see Figure 2A,B). As proposed by Dunn Warwick B et al. [34], positive ion mode primarily detects lipids, amino acids, and alkaline metabolites, while negative ion mode detects organic acids, sugars, and phenolics. In positive ion mode, the first two principal components, PC1 and PC2, respectively accounted for 41.0% and 23.6% of the overall variance. Conversely, in negative ion mode, PC1 and PC2 collectively explained 43.6% and 27.7% of the total variation. The PCA results indicated good reproducibility among tea samples of the same year, but significant metabolic differences among teas of different years, reflecting the reliability of the samples. To screen differentially expressed metabolites (DEMs) in Pu-erh tea from different harvest years, this study employed OPLS-DA analysis, incorporating all relevant metabolites as variables. Results showed that tea samples from all three vintages fell within the confidence interval with significant differences. The model exhibited high R^2^ and Q^2^ values, indicating strong explanatory power and stable reliability (Figure 2C–F). This demonstrates discernible variations among the three tea samples, highlighting the influence of aging duration on the metabolic profiles of Pu-erh tea.

**Figure 3:**
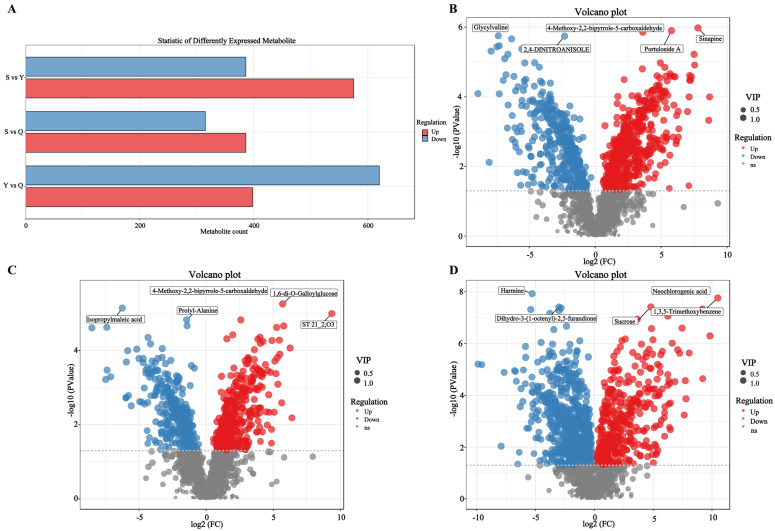
Statistical analysis of differentially expressed metabolites (DEMs). (**A**) Bar chart of DEM statistics. (**B**) Volcano plot of DEMs for S vs. Y. (**C**) Volcano plot of DEMs for S vs. Q. (**D**) Volcano plot of DEMs for Y vs. Q. (The *x*-axis represents the log2-transformed fold change in the quantitative value of a metabolite between the two samples; the *y*-axis represents the −log10-transformed *p*-value. Each point in the plot corresponds to one metabolite. The greater the absolute value on the *x*-axis, the larger the difference in expression level of the metabolite between the two samples. The larger the value on the *y*-axis, the more significant the differential expression, and thus the more reliable the selected differentially expressed metabolite. The size of each point reflects the magnitude of the VIP value; red points indicate metabolites that are significantly up-regulated, blue points indicate those that are significantly down-regulated, and grey points represent metabolites that do not meet the criteria for differential expression).

Based on 1825 annotated secondary metabolites, Differentially Expressed Metabolites (DEMs) were screened. Specifically, 961, 701, and 1018 DEMs were identified in the S vs. Y, S vs. Q, and Y vs. Q comparisons, respectively (Figure 3A). These extensive metabolite differences reveal that processing pathway (natural aging vs. pile fermentation) and aging time are core factors shaping the ultimate metabolome of Pu-erh tea.

The analysis further focused on key metabolites specifically enriched in the century-aged endpoint (S group) and directly associated with flavor quality. In the S vs. Y comparison (Figure 3B, Table S2), Sinapine, Portuloside A, and 4-Methoxy-2,2-bipyrrole-5-carboxaldehyde were significantly upregulated. Sinapine, acting as an antioxidant, might help delay lipid oxidation during long-term aging, maintaining quality stability. The bipyrrole derivative is associated with the microbial degradation of aromatic compounds [35]. Glycylvaline and 2,4-Dinitroanisole were downregulated, suggesting that the hydrolysis of small peptides decreases with aging, leading to the transformation of flavor precursors [36]. The accumulation of the bipyrrole compound hints at a unique, microbially driven reconstruction of aromatic frameworks during century-long aging, potentially contributing complex aromatic undertones to the “aged aroma.” In the S vs. Q comparison (Figure 3C, Table S2), 1,6-di-O-Galloylglucose was significantly upregulated. Classified as a hydrolysable tannin, this marks the uniqueness of long-term natural aging in tannin transformation. Its accumulation is linked to the catalysis of gallic acid esterification by microbial-secreted esterases [37], a process crucial for reducing astringency and enhancing the mellow thickness of Pu-erh tea. Isopropylmaleic acid was significantly downregulated, reflecting the consumption of organic acids during pile fermentation [38]. In contrast, in the Y vs. Q comparison (Figure 3D, Table S2), Neochlorogenic acid and Sucrose were significantly upregulated. The former, a phenolic acid, might increase due to phenol redox reactions driven by *Aspergillus*. Furthermore, the upregulation of sucrose in the aged sample (Y) is an interesting finding, potentially originating from the slow degradation of tea leaf fibers and other polysaccharides by microbial cellulases, regenerating oligosaccharides and disaccharides. This could balance the bitter and astringent taste of the tea infusion, enhancing its mellow and full-bodied sensation. Harmine was significantly downregulated, indicating that the decomposition of alkaloids during aging reduces bitterness, consistent with findings by Li et al. [35], demonstrating that the natural aging process also effectively decomposes bitter compounds. This suggests that the Y sample has already developed its own distinct metabolic characteristics, featuring richer potential sweet-tasting substances (sucrose), lower bitter compounds (harmine), and a unique pathway for phenolic substance transformation.

In summary, metabolomics revealed the ultimate metabolic characteristics and potential quality formation mechanisms of Pu-erh tea under different processing pathways. The natural aging pathway (represented by S and Y samples) exhibits stage-specific metabolic remodeling over time, characterized by the continuous hydrolysis of small peptides, the steady degradation of alkaloids (e.g., harmine), and the notable re-accumulation of sucrose in the mid to late stages—likely a result of microbial-mediated fiber degradation and sugar regeneration, providing the material basis for the mellow sweetness of aged raw Pu-erh. Concurrently, the specific enrichment of hydrolysable tannins (e.g., 1,6-di-O-Galloylglucose) and the emergence of aromatic derivatives like bipyrroles in long-term aging (S sample) collectively point towards a slow, complex flavor optimization process driven by specific microbial communities.

### 3.3. D Pathway Analysis of Differential Metabolites

**Figure 4:**
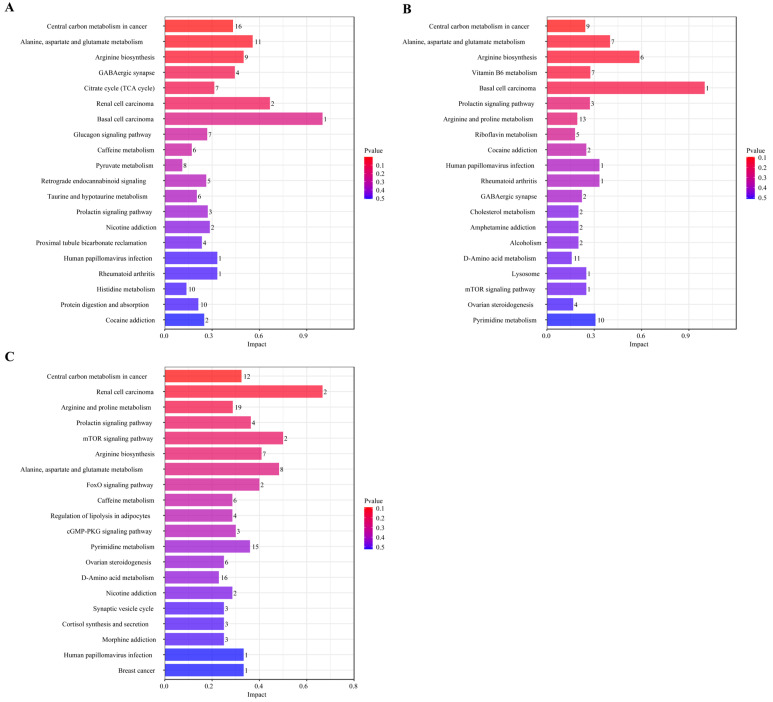
Aging-associated metabolic pathways in Pu-erh tea revealed by KEGG enrichment analysis. (**A**–**C**) KEGG bar charts for S vs. Y, S vs. Q, and Y vs. Q comparisons. Note: The *x*-axis represents the impact value of pathways enriched with differential metabolites; the *y*-axis represents the metabolic pathways. Numbers indicate the count of metabolites mapped to the corresponding pathway. Color corresponds to the *p*-value: redder colors indicate smaller *p*-values, and bluer colors indicate larger *p*-values.

To systematically analyze the functional metabolomic profiles of Pu-erh tea shaped by aging durations, we performed KEGG pathway enrichment analysis on the identified differential metabolites. Results showed 152, 132, and 110 significantly enriched metabolic pathways for the S vs. Y, S vs. Q, and Y vs. Q comparisons, respectively (Figure 4A–C). This initially reveals that the metabolic network associated with natural aging (S vs. Y) is the most complex, suggesting that prolonged natural aging drives more extensive and profound metabolic network changes.

It is noteworthy that pathways associated with “Central Carbon Metabolism in Cancer” were found to be significantly enriched in all comparisons. It should be noted that the pathway diagram in the KEGG database provides a summary of key carbon metabolism features, including enhanced glycolysis and glutamine metabolism in proliferating cells. In this study, the enrichment of this pathway and the key metabolites detected within it—including L-glutamate, L-aspartate, and citric acid—primarily reflect the active central carbon metabolism and amino acid conversion processes driven by microbial activity during the aging or pile fermentation of Pu-erh tea. These processes provide the energy and carbon skeletons necessary for the generation of flavor precursors. Claims pertaining to biological activity (e.g., anti-cancer effects) necessitate independent in vitro and in vivo experimentation for validation and are not inferred here.

The co-enrichment of alanine, aspartate and glutamate metabolism and arginine biosynthesis pathways further underscores the central role of amino acid metabolism in shaping Pu-erh tea quality. Glutamate and aspartate are key precursors for umami amino acids and crucial nodes connecting carbon and nitrogen metabolism [39]. The accumulation of arginine might be related to microbial-mediated nitrogen cycling and polyamine synthesis, influencing both the taste and potential bioactivity of the tea infusion. The activity of these pathways reflects how microbial communities, through enzymatic reactions during aging and fermentation, drive transamination, decarboxylation, and synthesis of amino acids, thereby regulating the final metabolite profile.

The core enrichment pathways observed in this study delineate the metabolic profile of Pu-erh tea under differing aging conditions. These pathways are centered on a metabolic framework focused on carbon metabolism (as the core functional module responsible for providing energy and carbon skeletons) and closely linked to amino acid metabolic networks. For the S samples undergoing ultra-long-term natural aging, the accumulation of specific metabolites is likely to reflect the cumulative outcome of sustained, subtle metabolic activities by microbial communities over a century. Conversely, the enrichment of these pathways in Q samples produced via modern pile fermentation indicates a trajectory of intense and rapid microbial metabolic evolution under conditions of high temperature and humidity. Therefore, notwithstanding the discrepancies in flavor and composition of the final products, the two core variables—aging duration and processing method—appear to collectively influence the diversified metabolite profiles of Pu-erh tea by regulating the intensity and duration of microbial community activity within this central metabolic module.

### 3.4. Analysis of the Microbial Community in Pu-Erh Tea Across Aging Years

**Figure 5:**
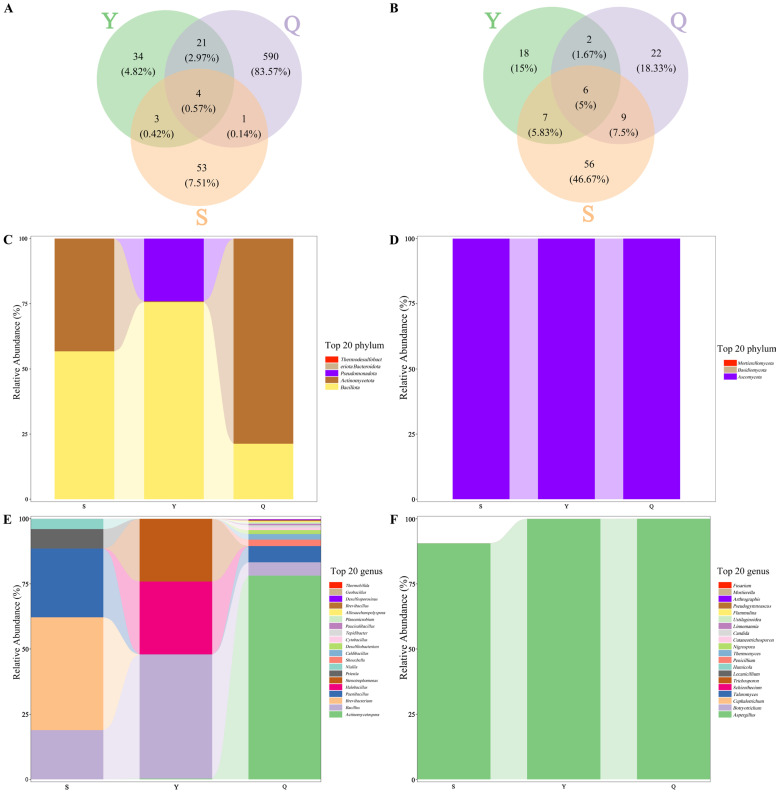
Microbial composition analysis of tea samples from different years. (**A**,**B**) Venn diagrams of bacterial (**A**) and fungal (**B**) taxa. (**C**,**D**) Community composition at the phylum level for bacteria (**C**) and fungi (**D**). (**E**,**F**) Community composition at the genus level for bacteria (**E**) and fungi (**F**).

The exceptional quality of Pu-erh tea is intricately linked to its distinctive microbial fermentation process. In the present study, we employed metagenomic sequencing techniques to delve into the structure and function of microbial communities present in Pu-erh teas with varying durations of storage. Our analysis encompassed three distinct tea samples. Utilizing high-throughput sequencing, we specifically targeted the bacterial 16S rRNA gene V3–V4 region and the fungal ITS1 region. Subsequent to the execution of rigorous quality control measures, we clustered the sequences at a 97% similarity threshold, ultimately identifying 706 bacterial operational taxonomic units (OTUs) and 120 fungal OTUs. Venn diagrams effectively depict the quantity of OTUs that are either shared or unique to each of the distinct tea samples, as evident in Figure 5A,B. Subsequent to the computation of OTUs in samples derived from diverse years, it was ascertained that the count of bacterial OTUs prevalent across all three tea samples stood at 4, constituting a mere 0.57% of the overall bacterial OTUs. Conversely, the number of fungal operational taxonomic units (OTUs) uniformly present in all three samples amounted to six, representing 5% of the total fungal OTUs. Additionally, the numbers of unique bacterial OTUs in the S, Y, and Q samples were 53, 34, and 590, respectively, while the numbers of unique fungal OTUs were 56, 18, and 22, respectively. This indicates that storage duration significantly alters microbial community structure. With increasing storage time, fungal communities exhibited stronger sample specificity, while bacterial communities tended to simplify, possibly related to microbial adaptive differentiation to the storage microenvironment and differences in metabolic functions. This study used Chao1, Simpson and Shannon to assess microbial alpha diversity (Table 1). The Chao1 index is a measure of species richness, while the Shannon and Simpson indices are a measure of species diversity. The results obtained demonstrate that the Q sample (ripe tea) exhibited a significantly higher bacterial Chao1 richness in comparison to the S and Y samples (raw tea). The S sample (century-aged raw tea) demonstrated comparatively higher fungal community evenness, thus indicating that long-term natural aging has the capacity to influence the structure of the fungal community. This phenomenon may be attributed to a gradual decline in nutrients, resulting in diminished initial fungal metabolism and reduced diversity and richness. However, as storage duration extends, the metabolic by-products may function as carbon sources, thereby fostering fungal growth and proliferation.

The species composition bar plots in Figure 5C,D illustrate the top 20 most abundant bacterial species at the phylum level. At this taxonomic level, the bacterial communities were characterized by the dominance of Pseudomonadota, Actinomycetota, and Bacillota. The S group tea sample was primarily composed of the phyla Bacteroidota, Thermodesulfobacteriota, Bacillota, Actinomycetota, and Pseudomonadota. In the Y tea sample, the dominant phyla were Bacteroidota, Bacillota, Pseudomonadota, and Actinomycetota. Meanwhile, the Q tea sample predominantly featured Pseudomonadota, Actinomycetota, and Bacillota. The three vintages shared Pseudomonadota, Actinomycetota, and Bacillota as dominant phyla. The results show that the S tea sample, compared to Y and Q, uniquely contained Thermodesulfobacteriota. *Thermodesulfobacterium* genus reduces sulfate under anaerobic conditions, generating trace amounts of hydrogen sulfide [40], which may participate in forming the “aged aroma” or special earthy odor of Pu-erh tea, though the specific mechanism remains unclear. In the fungal community composition, *Ascomycota* was the dominant phylum shared by all three samples. The dominant phyla in the S tea sample were mainly Ascomycota and Basidiomycota; in the Y tea sample, they were mainly Ascomycota, Basidiomycota, and Mortierellomycota; in the Q tea sample, they were mainly Ascomycota and Mortierellomycota. The S tea sample contained a large number of unclassified fungi, suggesting their potential involvement in the synthesis of unique flavor compounds.

Species composition bar plots were drawn for the top 20 species in bacterial abundance at the genus level (Figure 5E,F). At the genus level, the Q group exhibited significantly greater bacterial abundance than both the S and Y groups. The dominant bacterial genera in the S group tea sample were mainly *Brevibacterium*, *Paenibacillus*, and *Bacillus*. The dominant bacterial genera in the Y group tea sample were mainly *Bacillus*, *Halobacillus*, *Stenotrophomonas*, *Actinomycetospora*, *Paenibacillus*, *Caldibacillus*, and *Desulfitobacterium*. The dominant bacterial genera in the Q group tea sample were mainly *Actinomycetospora*, *Paenibacillus*, *Bacillus*, *Caldibacillus*, and *Desulfitobacterium*. In the middle and late stages of Pu-erh tea fermentation, *Brevibacterium* often becomes a dominant genus alongside Bacillus. *Brevibacterium* secretes short-chain fatty acids and proteases, promoting polyphenol transformation and thearubigin accumulation. *Paenibacillus* may promote tea cell wall decomposition through nitrogen fixation and pectinase production, releasing soluble sugars. *Bacillus* is a core functional bacterium in aged Pu-erh tea, driving catechin oxidation and fiber degradation through the secretion of polyphenol oxidase, cellulase, and pectinase, while competitively inhibiting spoilage bacteria [41].

With regard to the composition of the fungal community at the genus level, Aspergillus was the absolute dominant genus across all three vintages. Other minor genera were detected: in the S group tea sample, mainly *Trichosporon*, *Botryotrichum*, *Cephalotrichum*, *Schizothecium*, *Thermomyces*, *Talaromyces*, and unclassified *Hypocreales*; in the Y group tea sample, mainly unclassified *Hypocreales*, *Botryotrichum*, *Schizothecium*, and *Candida*; in the Q group tea sample, mainly *Aspergillus*, *Botryotrichum*, *Penicillium*, *Cephalotrichum*, *Lecanicillium*, *Talaromyces*, and *Pseudogymnoascus*. *Aspergillus* is a core functional fungus in Pu-erh tea fermentation. Its secreted α-amylase, cellulase, and pectinase directly catalyze tea polyphenol oxidation and polysaccharide degradation [42]. These microorganisms perform a crucial function in the generation of extracellular enzymes, in the breakdown of polysaccharides and polyphenols, and in facilitating the development and conversion of flavor compounds during the fermentation process of Pu-erh tea. Additionally, the as yet unclassified fungi within the S group have the potential to produce distinctive flavor compounds via secondary metabolic pathways, warranting further investigation in conjunction with metaproteomic analysis.

### 3.5. Multivariate Statistical Analysis of Differential Metabolites and Microbes

**Figure 6:**
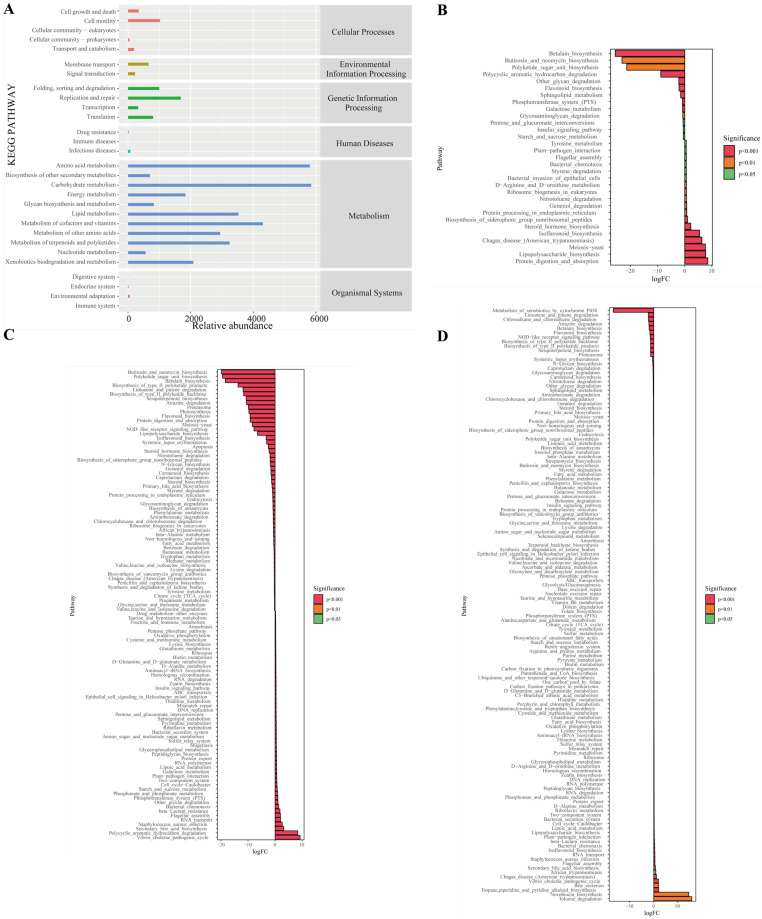
Statistical analysis of differential microbial metabolic pathways. (**A**) Abundance plot of secondary functional pathways at the bacterial genus level. Note: The *x*-axis represents pathway abundance (in KO counts per million), the *y*-axis represents KEGG level 2 functional pathways, and the rightmost column indicates the corresponding level 1 pathway category. This shows the average abundance across all samples. (**B**) KEGG pathways differentially abundant between S and Y groups. (**C**) KEGG pathways differentially abundant between Q and S groups. (**D**) KEGG pathways differentially abundant between Q and Y groups.

To investigate microbiota–metabolome interactions, KEGG enrichment analysis was performed on the three tea samples spanning a gradient of aging times. The main metabolic pathways covered Carbohydrate metabolism, Amino acid metabolism, Metabolism of cofactors and vitamins, Lipid metabolism, and Metabolism of terpenoids and polyketides (Figure 6A). Further analysis of differential metabolic pathways revealed that, compared to group Y, group S exhibited significant upregulation in 10 pathways and downregulation in 5 (Figure 6B). Significantly upregulated pathways included Protein_digestion_and_absorption, Lipopolysaccharide_biosynthesis, Meiosis-yeast, Chagas_disease_(American_trypanosomiasis), and Isoflavonoid_biosynthesis. Pathways such as Betalain_biosynthesis, Polycyclic_aromatic_hydrocarbon_degradation, Other_glycan_degradation, and Flavonoid_biosynthesis were significantly downregulated. In the comparison between the Q and S groups, 18 pathways were significantly upregulated and 33 were significantly downregulated (Figure 6C). Significantly upregulated pathways included Vibrio_cholerae_pathogenic_cycle, Polycyclic_aromatic_hydrocarbon_degradation, Secondary_bile_acid_biosynthesis, Staphylococcus_aureus_infection, Butirosin_and_neomycin_biosynthesis, Polyketide_sugar_unit_biosynthesis, while Betalain_biosynthesis, Biosynthesis_of_type_I_polyketide_products, Limonene_and_pinene_degradation, Biosynthesis_of_type_II_polyketide_backbone, Sesquiterpenoid_biosynthesis, and other pathways were significantly downregulated. In the comparison between the Q and Y groups, 104 pathways were significantly upregulated and 43 were significantly downregulated (Figure 6D). Significantly upregulated pathways included Tropane, piperidine_and_pyridine_alkaloid_biosynthesis, Vibrio_cholerae_pathogenic_cycle, Chagas_disease_(American_trypanosomiasis), while Metabolism_of_xenobiotics_by_cytochrome_P450, Limonene_and_pinene_degradation, Chloroalkane_and_chloroalkene_degradation, Atrazine_degradation, and other pathways were significantly downregulated. The functional changes observed in microbial communities in this study suggest that the potential metabolic functions of Pu’er tea’s microbial communities may undergo corresponding evolution with extended storage duration, involving alterations in the relative activity levels of various secondary metabolite biosynthesis and degradation pathways. These findings provide preliminary clues and research directions for further exploring the interactions between microorganisms and metabolites.

**Figure 7:**
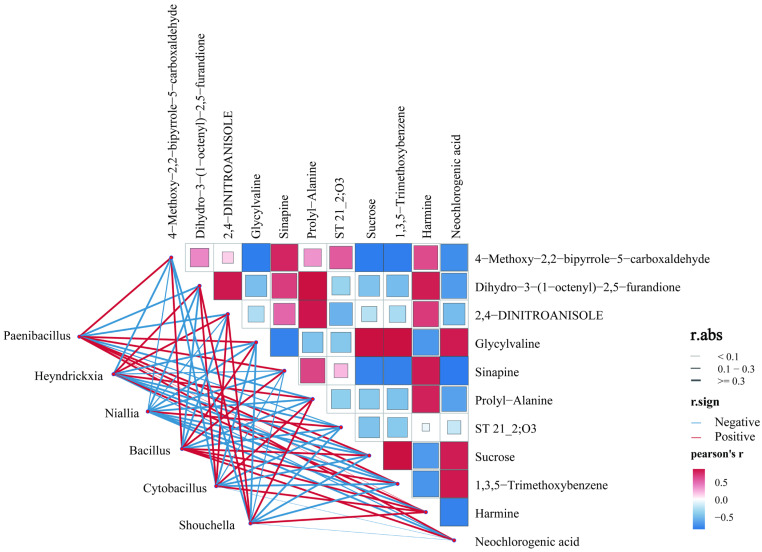
Correlation heatmap of microorganisms and differentially abundant metabolites.

To delve into the potential microbial factors underpinning the aforementioned metabolic disparities and their interconnections, we initially identified 31 bacterial genera that exhibited marked differences between the groups (LDA > 2, *p* < 0.05) utilizing LEfSe analysis. From these, six key differential bacterial genera—*Paenibacillus*, *Heyndrickxia*, *Niallia*, *Bacillus*, *Cytobacillus*, and *Shouchella*—were selected for Pearson correlation analysis with differential metabolites to construct a microbe–metabolite interaction network (Figure 7). Results showed that the relative abundance of *Paenibacillus* correlates positively with sucrose, neochlorogenic acid, and the potential aroma compound 4-methoxy-2,2-bipyridine-5-carbaldehyde. This correlation pattern aligns with existing literature reporting pectinase activity in certain *Paenibacillus* strains [43]. Therefore, we propose a hypothesis: during the aging process of Pu-erh tea, bacteria of this genus may participate in the decomposition of the tea leaf matrix by secreting cell wall-degrading enzymes (such as pectinase), potentially influencing the release of soluble sugars. This could be one of the sources constituting the flavor base for the sweet and mellow taste of the tea liquor. The relative abundance of *Bacillus* was positively correlated with multiple metabolites, including glycine-valine, erucic acid, sucrose, 1,3,5-trimethoxybenzene, and neochlorogenic acid. Previous studies have confirmed that *Bacillus* is a dominant bacterial genus during Pu-erh tea fermentation [44]. Based on these correlations and literature evidence, we hypothesize that *Bacillus* may play a crucial role in processes such as the oxidative polymerization of catechins and the degradation of tea cell wall polysaccharides. These processes are typically accompanied by the generation of pigments like theaflavins and thearubigins, as well as flavor precursors such as gallic acid, thereby contributing to the enhanced richness of the tea liquor.

It must be emphasized that the correlations revealed in this section represent statistically significant co-occurrence relationships, primarily aimed at establishing functional hypotheses linking “microbial presence” to “metabolite output.” These significant associations strongly point to *Paenibacillus* and *Bacillus* as key candidate functional bacteria driving the formation of aged Pu-erh tea flavor. However, their precise functional roles and causal mechanisms require final validation through subsequent studies, such as pure culture inoculation fermentation experiments.

## 4. Conclusions

This study employs non-targeted metabolomics and microbiome technologies to conduct exploratory analyses of Pu-erh tea under different aging conditions, with the aim of characterizing its metabolite and microbial community profiles. Preliminary findings, derived from in-depth case studies of century-old precious samples (1920 raw tea), mid-aged samples (1999 raw tea), and modern pile-fermented ripe tea (2024 ripe tea), suggest that microbial communities may play a pivotal role in determining the final state of Pu-erh tea aging. The potential functional mechanisms underlying this influence are the subject of further exploration.

Metabolomic analysis demonstrates that the chemical profiles resulting from natural aging and pile fermentation are distinctly different. The hallmark characteristics of prolonged natural aging may be attributed to the specific enrichment of hydrolyzable tannins, the re-accumulation of sucrose, and the emergence of complex pyroloquinoline aromatic derivatives. This metabolic signature indicates that the process of natural aging is a gradual, sustained, microbially driven process, characterized by sugar regeneration from fiber degradation, profound tannin transformations, and flavor framework restructuring. Collectively, these processes form the underlying material basis for the mellow, sweet, and “aged charm” qualities of aged Pu-erh tea. Conversely, the pile fermentation process (Q samples) has been shown to rapidly simulate the chemical characteristics of natural aging, yet it appears to lack the capacity to fully replicate the intricate network of metabolites formed over centuries of natural aging.

KEGG pathway enrichment analysis revealed that “central carbon metabolism in cancer,” “alanine, aspartate, and glutamate metabolism,” and “arginine biosynthesis” represent core metabolic nodes commonly enriched across samples from different processing pathways. This finding indicates that, irrespective of the duration of aging or the method of processing, a functional module centered on central carbon metabolism—which provides energy and carbon skeletons—and closely linked to amino acid metabolism may be pivotal in determining the quality of Pu-erh tea. The presence of elevated concentrations of key metabolites (e.g., L-glutamate, L-aspartate, citric acid) within the central carbon metabolism pathway is indicative of its active state during the aging process of tea. It is imperative to note that any claims pertaining to its biological activity are to be substantiated by independent experimental validation in future studies; this study offers solely descriptive findings.

A comprehensive analysis of the microbiome was conducted, revealing that the duration of storage and the processing pathways employed have a substantial impact on the structure of the microbial community. Extended natural aging appears to simplify bacterial communities while increasing fungal evenness, accompanied by the specific emergence of anaerobic bacteria such as *Thermodesulfobacterium*. The potential association of this phenomenon with the formation of “aged aroma” warrants further investigation. Correlation analysis further constructed a “microbe-metabolite” interaction network, thereby identifying *Paenibacillus* and *Bacillus* as key candidate functional genera. The significant positive correlations observed between the bacteria and sucrose, neochlorogenic acid, and pyridine derivatives led to the hypothesis that these bacteria may play a crucial role in shaping Pu-erh tea’s flavor profile. This process is facilitated by the secretion of polysaccharide hydrolases, such as pectinase and cellulase, which are responsible for the degradation of tea cell walls, the release of soluble sugars, and the catalysis of the conversion of phenolic compounds.

In summary, the present study provides supporting evidence for the following hypothesis: the ultimate quality of Pu-erh tea is the result of the combined effects of “processing pathways” and ‘time’ acting upon the “microbial community,” which systematically reshapes the tea leaves’ “chemical profile” through its metabolic activities. Natural aging is defined as a progressive metabolic remodeling process driven by specific microbial communities that cannot be fully replicated, while pile fermentation intensifies and accelerates its key pathways. These findings offer new analytical perspectives and data references for understanding the quality formation principles of Pu-erh tea (especially rare century-old aged teas) and provide preliminary theoretical foundations for future applied research on precisely directing ideal flavor profiles through targeted regulation of microbial communities. Subsequent research will focus on isolating and identifying the aforementioned key candidate functional microorganisms, validating their functions through inoculation fermentation experiments, and ultimately laying the foundation for achieving targeted design and regulation of Pu-erh tea flavor and quality.

## Figures and Tables

**Table 1:** Alpha diversity analysis of tea samples of different ages.

Sample	Chao1		Simpson		Shannon	
	Bacteria	Fungi	Bacteria	Fungi	Bacteria	Fungi
S	31.74	54.30	0.76	0.57	2.60	2.29
Y	27.84	21.33	0.74	0.24	2.06	0.88
Q	263.80	24.97	0.71	0.10	3.22	0.42

## Data Availability

The original contributions presented in this study are included in the article/Supplementary Materials. Further inquiries can be directed to the corresponding authors.

## Supplementary Materials

The following supporting information can be downloaded at https://www.mdpi.com/article/10.3390/foods15050916/s1: Table S1. Secondary metabolites in Pu-erh tea from different years. Table S2. Differentially Expressed Metabolites in Pu-erh Tea from Different Years.
