# Supplementary material for: Metabolic and Microbial Community Profiles of Century-Old Pu-Erh Tea: An Integrative Metabolomic and Microbiomic Analysis
Source: Foods. 2026 Mar 6;15(5):916. doi: 10.3390/foods15050916 (PMC12985185; doi:10.3390/foods15050916)
Supplement: Supplementary file 1 [file foods-15-00916-s001.zip › foods-4111129-supplementary.pdf]

Table S1. Secondary metabolites in Pu-erh tea from different years

name	S1	S2	S3	Y1	Y2	Y3	Q1	Q2	Q3
Glycerophosphocholine	561465019.285	73748440.271	524673216.358	1117488059.397	989611599.982	1084361236.241	493696154.435	65547166.557	77773022.941
Kaempferol 3-O-arabinoside	30517427.686	17916600.932	28108235.044	4130886.506	3829242.535	2891179.374	8669363.720	13670622.965	13591468.748
Betaine	328746562.860	213402362.402	364222038.972	229470563.612	218622916.464	245618488.967	504941869.283	487574451.925	507648870.441
Isoquercitrin	614740654.882	787240806.933	1319596001.060	1627795672.573	1427549709.527	1218918256.107	523789557.080	601358062.802	534871406.359
18-Oxooleate	92010642.737	49579942.004	100047582.240	39161116.364	32651159.239	34949689.452	28830033.913	30059188.928	29367496.524
Saccharopine	9885369.121	5612850.247	8600346.136	18694488.648	4404447.619	17772514.328	10723582.421	10170082.104	9458901.259
Prostaglandin H2	11431203.716	18985677.500	7735584.750	2899739.590	799743.247	614933.642	345122.205	522865.383	1529611.641
ACMC-20dgc9	97603903.118	92717776.563	400813726.617	93148648.811	75117436.151	64385883.446	78040164.186	128854667.780	50735107.272
L-Lysine	78170507.738	46010404.881	67625058.700	714995284.912	618779897.225	640239963.888	272032349.164	317147787.657	311228214.770
9,10-Dihydroxystearic acid	202582678.113	124751364.391	205546213.247	8924320.491	7108864.937	7840291.179	60423416.270	65971022.053	64763876.948
Afzelin	176947989.132	118135628.051	192753583.729	177375831.823	182792886.592	147425328.892	301892311.856	338317210.736	322051748.894
15,16-epoxydolabrene	6200443.234	11680441.885	21169176.178	2286215.492	6362265.137	1796974.915	17371439.120	4380155.680	14091616.048
Floionolic acid	521846230.833	290040190.460	502482156.703	14970896.916	14262346.757	14573333.523	163743109.733	161979444.613	188290610.535
FA 18_1;O	229542587.842	140913635.038	221130815.157	369853168.614	315230750.280	312316567.281	477144015.945	513936971.350	485498838.903
Cortol	18280057.558	10386540.365	15070945.149	36699169.504	34854912.288	35175729.739	53204429.891	43765257.823	38996314.191
N-Succinyl-L,L-2,6-diaminopimelate	37160436.681	24980063.123	37509458.671	174116520.107	149033709.111	173170296.582	192743088.715	204869637.235	215429594.508
Riboflavin (Vitamin B2)	42678618.127	24437355.814	34324875.212	3693539.211	5250076.129	334212.074	23840471.135	28851222.602	31886433.072
2'-Hydroxygenistein	244011446.012	135456789.679	229507387.061	237897884.692	219348388.798	220254527.635	403946313.711	411430882.678	430442177.218
N2,N2-Dimethylguanosine	17684989.693	11017288.771	18211009.518	15357488.013	14958266.417	14744816.211	44365952.827	47882086.441	43171996.297
(R)-3-(4-Hydroxyphenyl)lactate	23383944.819	19663636.427	46518120.774	17899583.421	13058649.866	18920945.456	23501135.423	17605308.167	15658037.006
Guanosine	198456701.036	89907185.875	177395603.568	182879698.267	168139332.413	172233288.277	172408225.365	209341369.576	213248436.944
11-HpODE	75214077.545	10507811.106	16476462.263	13976226.054	12874598.092	12558462.787	22133150.716	23109307.961	5886389.307
P-Coumaraldehyde	67048878.794	38553671.451	70795771.558	4459073237.714	3982243962.361	4191730855.627	48317392.394	142499989.947	145781482.429
Theophylline	84822533.929	47787053.626	85394343.874	89507952.374	87163740.570	80231601.169	86288641.950	76703512.901	88165573.048
Oleamide	148355909.864	96351630.264	188002171.396	76490783.470	48528147.245	61093456.268	59375772.910	73945164.967	71041733.413
2-O-Methyllicodione	110064800.407	43386451.477	109800324.502	38468687.272	35875520.222	34574144.069	47742517.659	51705805.455	52803813.782

name	S1	S2	S3	Y1	Y2	Y3	Q1	Q2	Q3
Galactosylglycerol	126702731.258	80232814.966	122869482.801	189473958.753	180201764.478	161748257.672	100833157.802	108033223.307	105653432.318
7-ketolithocholic acid	134392858.459	19256367.906	41821054.368	21561608.881	3914310.817	882196.968	3493257.219	4310161.305	5914590.890
Rutin	962063386.786	585041976.508	935048360.530	1040588242.864	988101279.939	946088848.519	360009330.868	366382900.496	388729324.073
16-oxo-palmitate	37063022.767	21937078.106	38709237.181	1182254.209	880274.512	893510.981	15069452.537	15908392.049	16810662.114
2-hydroxyphytanic acid	82187568.865	49993850.335	88473018.412	16620170.780	15270116.556	13429535.319	124935708.167	116855055.286	126995304.184
FA 20_3;O4	2710761.277	1626954.912	3645961.013	285509.235	319750.812	296284.865	2363241.110	2471931.343	729054.811
Secoisolariciresinol	9158937.461	7780290.111	8778766.744	8026247.869	5221124.109	9512941.114	15381483.907	16229879.648	15508237.255
Linoleamide	42242117.056	14160613.612	43304081.854	87715175.225	41279558.181	34829829.481	13092003.837	15763348.709	27678719.266
ACMC-20m5rp	143260038.412	83092931.460	144310386.273	95523671.692	73893738.951	92928013.775	86016968.918	95841610.981	70185908.944
(9R,10E,12Z,15Z)-9-hydroperoxyoctadeca-10,12,15-trienoic acid	15137748.257	10243006.830	55415168.047	27614551.893	22251227.280	21781216.493	22772397.087	20379626.609	24271912.704
Sucrose	72092466.747	43179531.700	66140510.668	537851354.763	497643896.338	491465611.920	18153671.216	17933542.013	18539069.125
2'-Deoxyguanosine 5'-monophosphate	195538860.570	139565620.872	197188718.689	322057242.212	259918505.317	253234783.308	24069388.409	16685067.808	20632620.448
FA(18_4)	175151668.780	114200184.470	177474228.240	338539166.069	45186508.194	45000093.611	208722132.642	189835281.573	188305783.267
9(S)-HPODE	77334206.077	67528487.824	172064570.782	60573191.462	115363898.468	70975587.582	58151487.589	93434775.179	102054324.494
Meloside	623605042.902	388026952.734	512072791.074	844537801.302	589325942.707	263557837.155	121161887.980	240885604.515	332144061.211
Prostaglandin A2	7514616.802	994261.293	119715489.264	54010846.697	54821621.814	38043716.417	38181361.256	7434283.468	7082194.320
Astaxanthin	762523366.634	388035031.950	730919775.509	291283326.535	143726596.331	295949393.486	383555450.365	184464604.104	289020849.482
6-Ketoprostaglandin E1	43490438.448	116899747.620	173684421.761	8906814.691	8173863.890	6878596.015	48099432.410	47969013.612	13294743.051
2-methyleneglutaric acid	3467816.310	1807298.630	2621150.809	35421524.613	31771195.106	34037790.184	619503.544	2147899.961	914704.792
16-Hydroxyhexadecanoic acid	206905278.333	79113743.825	214737680.923	20867546.042	16069604.957	15207933.702	30565288.775	36282582.755	44374397.736
Prostaglandin-c2	394266432.274	52140710.937	123610394.328	115424078.699	87324595.716	87541403.235	77422797.141	82498738.401	93276818.291
3a,21-Dihydroxy-5b-pregnane-11,20-dione	7942789.421	6433680.439	9745199.302	6067634.265	2442368.117	3028012.639	2393838.972	4908514.873	3968254.624
Machiline	369563903.516	235245767.109	350332301.879	170232568.791	141391340.203	145434334.261	136672709.117	138670598.427	138449847.623
Gamma-Linolenic acid	1263041713.836	921412754.341	1462358887.093	34146693.879	29710389.934	29908872.907	50825424.261	51078196.216	102066322.756
Fisetinidol-4beta-ol	126334080.096	20416793.243	135755914.058	840525420.545	757433524.458	192537042.889	25904179.603	28343975.454	23068712.561
1-Hydroxyisoquinoline	258593784.909	24781386.077	40348605.522	10575100.232	10422575.619	9044015.712	33184508.155	19848614.390	17760286.795
aureusidin 6-O-glucoside	841205734.450	524129524.534	427278846.289	1315122167.997	926664638.040	1006574013.726	410092512.606	314907726.034	383003536.895

name	S1	S2	S3	Y1	Y2	Y3	Q1	Q2	Q3
Sphinganine	332482018.439	168050277.601	640236171.583	388663948.850	121048407.146	135411313.867	153996765.666	153231122.037	312319225.834
9,10-DiHOME	192631680.913	112058765.814	172047177.260	9382003.925	7808989.151	8074609.157	39213497.332	43358399.485	40345885.531
4-Hydroxysphinganine	3326126.838	849622.211	2852541.328	7640669.387	1569442.426	1468880.547	8767218.826	10064910.329	8388582.741
ent-cassa-12,15-dien-2beta-ol	25133533.247	12747793.757	21959070.176	91490.403	127466.403	210941.030	4028291.998	5378306.128	6753405.730
Indan-1-ol	256696037.479	154475708.874	253855666.642	544492760.122	495454571.565	501767940.193	133893163.581	139254766.115	134536123.362
Prostaglandin C1	281681145.076	145167188.199	228733542.650	70707288.936	49954323.272	46624715.946	74750664.253	84577127.935	77624104.255
Trioxilin A3	2694997181.839	1557568842.111	2618891577.134	57600308.144	48779586.128	51231397.790	1130088462.969	1236913321.067	1283193719.004
2-Methylquinoline-3,4-diol	2595794.855	28038759.443	1635690.369	7947765.313	912166.909	1045262.567	80055070.018	85617967.496	79753858.217
Linoleic acid	326519484.395	102694922.661	324758744.071	48802378.193	23396033.308	19803902.139	65174135.133	58161060.721	132048152.651
7-hydroxylysine	59952425.840	40791500.865	114512968.877	152217246.736	59254830.427	52796009.438	37884220.644	11031737.187	133527331.871
4-Oxocyclohexanecarboxylate	209085628.616	117895763.435	211622956.827	92872239.109	82758317.754	87409235.295	134463959.757	145925865.526	141497451.939
N-benzylformamide	76808782.435	102165207.345	155608042.472	24868119.471	19990474.291	18763593.520	33956344.609	36215227.861	32745746.512
N-Formyl-L-glutamic acid	3951056.373	2291204.326	4001550.109	13305478.810	6466089.557	6960863.479	2818342.510	20169902.526	4081020.464
18-Hydroxyoleic acid	130724373.703	74120583.953	130178732.579	7583400.802	5757396.671	7717239.595	34965338.593	39467525.781	37674527.940
(5R,6R)-2,6-dihydroxy-5-(hydroxymethyl)cyclohex-2-en-1-one	15546082.012	9563665.874	14736565.287	78084949.406	69584078.054	70188402.355	164156817.700	157705566.971	158718176.244
10S-HpOME	2239719151.034	1350191697.949	2243902395.869	78655312.939	65020054.840	65627646.748	738051656.746	767344956.823	779730597.782
LeachianoneG	70689079.396	42998468.598	67734066.909	13817561.270	14746756.419	10962633.419	42774938.013	44357041.425	44122433.740
12,13-EpOME	83983749.257	68435699.392	73340681.051	17188049.331	9960724.943	8413841.255	4179052.806	4530665.136	16063910.245
9,10-Epoxy-18-hydroxy-octadecanoic acid	244396935.933	143580984.713	237839972.051	14688319.626	13579200.604	14678453.201	55711414.632	63126451.615	58347120.822
Tetrahydrocorticosterone	39353870.082	12451561.319	24212332.409	1301651.968	507873.086	102962.911	3569004.597	2630303.808	5084099.940
(R)-Mandelamide	31133711.727	26369254.111	32552090.783	14087974.032	3644447.862	3330121.274	5542442.982	5678647.631	13168714.815
2-Furancarboxaldehyde	45700505.983	27662254.021	45750656.927	26915171.153	86017346.056	22494797.969	59121532.268	72271215.616	173860618.227
FAL 16_1	43008472.382	24233902.446	45110451.049	20016389.493	15997624.175	17978552.928	22795775.725	23784585.514	23868246.551
Cytidine	60169994.058	34630083.290	61285580.458	56647539.168	52799483.392	54197125.885	150310880.141	155470746.423	148960024.740
8(R)-Hydroperoxylinoleic acid	94622836.551	56449044.160	89250437.378	72025218.043	63938959.208	72669030.239	42346189.835	46765172.413	44842618.778
Gibberellin A15	101478423.236	57094904.407	38426407.384	69974991.156	94538756.586	32672495.118	138141658.637	67919187.569	80091066.675
Neurosporene	7527086.201	1847996.766	6279323.420	3373923.586	12091758.558	13488320.877	5680786.412	4530145.364	3309944.915
Gibberellin A53	119333	587773	510695	185972	166035	171602	118960	148218	107297

name	S1	S2	S3	Y1	Y2	Y3	Q1	Q2	Q3
	04.850	5.540	3.361	0.253	8.375	8.496	21.062	23.512	65.663
FA 20_5;O2	195871180.735	40716357.876	83713661.610	153414461.605	47371842.602	151585418.424	99077370.984	171941909.317	238590537.930
10R-HpODE	8706297.289	14064359.977	36191591.866	5679669.649	2098083.125	5365152.223	2877166.756	11805074.835	2825085.429
Cyclohexanone	26968704.843	13700537.868	23366483.124	5086536.780	6522500.790	145740040.048	9760966.758	10331604.551	10741745.070
20-Hydroxy-leukotriene B4	499966562.108	78414314.743	124382328.006	100583451.007	99760506.884	81847946.414	156163911.215	169614550.725	175185646.428
(S)-2-Methylbutanal	539457917.444	328889320.059	557293283.743	2535610669.127	2271952967.481	2284276945.462	718139171.626	807056200.980	768696655.968
5-Hydroxylysine	60576746.236	32839049.447	56014250.932	62395036.297	61941814.011	15163930.348	32851457.209	36212796.668	10387696.919
2-Aminoisobutyric acid	51333284.501	29364525.353	46661446.572	111239800.711	100676237.246	93031984.491	21215192.604	24372481.209	26950530.845
FA 7_3;O4	69877798.025	40880373.047	66903554.855	13453662.451	20678406.789	17650097.288	20715679.302	23934167.963	20522569.527
D-Ribose	3523583.394	4641597.257	25675670.530	1657290.642	10836220.220	9106183.277	13240542.812	17754739.643	1597931.799
Aminocaproic acid	27897533.201	78727847.822	35295483.666	96795640.557	87081748.600	96414444.589	266177325.972	132888378.977	284096623.960
Pantetheine	7452168.677	900154.697	6974778.141	528729.017	447543.636	518477.340	7815428.490	7939863.058	7885673.463
trans-4-Carboxymethylenebut-2-en-4-olide	43751190.166	23705404.318	43164105.712	57516439.926	27685867.841	27439894.439	21734029.772	24290023.014	23237950.387
Prostaglandin E2	775135174.410	1050478143.849	1423082779.277	19742454.174	15828385.600	17625070.551	68440246.400	75229430.312	72712436.611
Delphinidin 3,5-diglucoside	57909972.167	35386790.380	354131191.503	63305530.634	386343186.229	61060762.086	138139855.844	71021645.155	39697407.563
cyanidin 3,7-di-O-beta-D-glucoside	169885430.002	42462522.170	25472411.260	70754694.283	67573331.936	62584833.171	54481238.362	63408249.216	48000308.761
3,3',4'5-Tetrahydroxystilbene	26726196.318	17680040.900	27865884.832	21257457.082	17016443.418	17106614.984	137593146.431	135108554.938	130720616.156
Kanamycin B	26167761.890	12441085.079	17816972.785	1163239.372	1702549.527	2139331.450	14155808.277	12419910.552	13999231.715
10-oxocapric acid	10715041.180	13049046.385	3327422.823	5719708.346	3851531.690	3209077.977	1188463.509	1147743.297	1184448.601
Dehydrochlortetracycline	656804047.681	397646690.386	676405492.607	1654115926.521	2088957959.252	2494745091.710	59740947.487	53673670.236	40654713.850
2-Aminovalienone	9122256.125	9130143.405	11072459.450	6432119.663	5002403.503	5859473.220	10747172.078	11110952.959	9719878.101
Pheophorbide a	339075869.570	49903849.199	41762584.701	14534900.030	11752430.176	20933063.817	6884739.904	10996838.533	40237690.071
(2R,4S)-1,7,7-trimethylbicyclo[2.2.1]hept-2-yl diphosphate	35197048.386	18404744.487	34065341.579	124171996.448	105262195.833	109296339.530	4053688.171	4513853.021	4901593.501
2-oxo-5-Methylthiopentanoic acid	106968987.977	71013463.395	110716952.423	425561777.057	375207125.726	313124162.967	3707076.188	3940361.100	3659121.515
Butirosin B	1596685.965	1843600.437	5552271.238	1889078.849	5642438.306	108000944.910	214534793.971	271815485.806	72433237.239
1-Pyrroline-2-carboxylic acid	83761295.345	22416687.079	90952852.280	94174207.748	88145725.234	87977535.115	26584497.379	29026319.662	26589177.438
S-Citramalate	13819829.046	5603982.601	13800763.724	61791058.623	50105834.199	54472595.158	15040672.878	19546929.211	12956567.213
N-acetylalanine	175160	106539	185617	244318	254186	186390	874599	944882	167841

name	S1	S2	S3	Y1	Y2	Y3	Q1	Q2	Q3
	332.658	542.968	804.955	03.491	02.383	10.904	85.802	61.826	707.320
Isopropylmaleic acid	5028479.734	3268242.771	4775586.542	30492279.963	21833457.046	26241729.853	308466582.415	360869926.780	317959460.757
Avermectin B1a aglycone	25439629.456	16197264.015	40111376.089	99252407.421	79196315.522	76210571.071	29820201.271	31032812.528	31498194.748
6,8a-Seco-6,8a-deoxy-5-oxoavermectin 2a aglycone	34146755.074	59765484.532	72448880.126	36521703.113	84326042.752	76621829.973	54676323.229	49985051.450	51988691.015
Tetrahydrodeoxycorticosterone	5410575.187	3035634.262	4484000.156	2759763.487	1524891.091	2377535.989	3206506.761	2742530.406	2643529.658
Indole	824877.008	1909863.651	3085139.075	8830567.674	7707436.481	7179022.502	8196514.431	7050496.813	7275621.156
8,12-Diethyl-3-vinylbacteriochlorophyllide d	6213502.417	10654833.353	44957819.174	8628303.789	1028106.878	587370.162	590495.413	2659133.444	4494772.178
Sphingosine 1-phosphate	38605431.169	19193871.596	34187346.306	261887.396	191095.893	188443.692	12749281.511	12529389.780	11546909.136
11-cis-Retinol	1021265.594	124894.875	4955003.551	1985604.588	882869.699	2187411.018	14172698.240	11942420.102	21745570.222
Ajmaline	1157405965.255	201365901.101	44721085.587	111451848.873	22873037.691	18711350.972	23136964.642	79125155.093	127047778.407
Pyridoxate_1	147430293.809	93385179.572	143531259.894	89623077.807	99113657.671	119837119.177	506761722.126	540791176.890	559101797.176
Prostaglandin F2alpha	5316816.272	17362576.400	7721572.108	2973034.074	2907987.340	96096.999	14632062.407	17464685.690	16557768.022
Ketospirilloxanthin	113304590.375	30832387.451	139848533.548	79551417.475	50931305.904	35123882.638	71516987.849	51849436.599	63906153.545
Delphinidin 3,5,3-triglucoside	14528010.397	9455992.824	15325403.742	25721295.479	15790096.427	26079432.832	6689208.869	6705876.746	6499284.775
Glutaric acid	10106390.605	4087784.469	8773166.208	87204176.492	84225634.606	82206336.374	93920786.408	5178160.232	3992953.091
Hesperidin	21322776.406	13409112.971	73380176.105	254284847.612	71724499.745	207390767.442	78598676.362	9896877.394	31564465.541
Deoxymyxol	9042556.976	2952040.122	7960212.063	61444542.947	44742676.903	13976121.099	11632806.962	2952114.691	10075080.100
ST 29_3;O	3026311.146	1513088.643	1624024.628	4109078.184	4508548.387	4549947.087	22693435.474	25349176.565	25881021.724
miconazole	32834477.156	22140555.251	44160816.457	10408128.734	10218385.449	10656752.839	27880012.527	30021682.200	29072995.585
5-Methyl-2-furancarboxaldehyde	960055317.992	131775344.963	322365411.979	286364131.150	238758770.267	253751765.438	174122401.359	146683346.847	187184450.339
Orientin	82720097.706	51260442.801	17855719.968	117312553.781	85060416.742	73862939.739	51482278.163	42822640.080	51674166.038
Betonicine	175114105.820	112354504.571	177948950.667	163875176.202	3981545.506	152976462.584	313890262.383	367332223.303	359542316.698
2,3-Dihydro-7-methoxy-2-(3-methoxy-4,5-methylenedioxyphenyl)-3-methyl-5-(1-propenyl)benzofuran	40001584.681	23132030.036	43028796.236	48440710.120	31749332.200	32327334.787	46457718.626	59049002.591	58045328.383
2,5-Dimethyl-2,4-hexadiene	10072589.903	19150980.532	16194550.209	5344255.267	4277115.827	5362945.625	9599596.696	13262752.749	9006486.465
Armillaripin	32962845.163	22516953.891	54269260.363	12072754.492	11896829.514	11785086.776	33636242.985	34410814.790	36827309.437
3-O-p-Coumaroylquinic acid	225557736.763	148466019.591	229811132.600	204224130.530	157851830.757	204889562.966	84083134.755	82013161.192	70773624.441
Ganodermanondiol	2918634.618	657084.928	294340755.661	156468156.534	64789033.868	2227656.260	5742117.934	3192296.501	50080415.771
hydroxyoctadecadienoic acid	165853235.133	96386495.486	139330045.062	136105306.504	123091949.308	125567905.324	64571231.112	67810779.800	67702832.241
LysoPE (16_0_0_0)	206999	870640	161820	330435	150865	124913	225278	277368	399663

name	S1	S2	S3	Y1	Y2	Y3	Q1	Q2	Q3
	44.056	7.410	79.135	957.031	751.834	417.770	21.739	62.266	13.565
Corchorifatty acid A	9403696.746	26861349.679	76935612.159	6682140.766	44878951.274	6628507.429	12480440.853	28123312.859	12521051.601
Etiocholanolone	751805.723	539370.153	844331.687	30129638.080	28128556.218	28378850.935	6193294.502	7048464.495	7199902.602
3,5,5-Trimethyl-2-cyclohexen-1-one	79461832.683	45818279.466	70437247.860	159208815.228	145230996.730	137767277.181	34258516.982	39161086.531	32344146.580
Calendic acid	17880191.240	9660122.594	19649626.049	110126637.254	96019853.002	77803005.835	32672748.210	14315924.318	15242225.612
S-methylazathioprine	56742847.556	102656620.658	54209361.912	67822997.342	60247335.475	61015242.813	74383212.811	79086385.071	78142041.017
Avocadene	278262394.865	161439401.848	264410515.698	140669444.798	129303903.160	124075215.815	151554932.951	148328351.908	146173548.830
(R)-2-Hydroxysterculic acid	473790874.409	248067487.901	749420999.274	291457141.581	250848838.065	261464501.460	288768873.288	305685588.857	305984738.857
dihydroalbocycline	313906946.836	187247020.193	296510338.543	181766159.283	155497376.052	161761679.030	171718083.068	191759284.938	177460878.075
Homocarnosine	49908806.200	35803317.194	46682483.291	132979754.507	140338917.935	132006975.242	9202176.267	11201574.996	11228202.520
Gibberellin A5	23902163.133	12932582.083	22876736.600	7390070.021	1304475.012	1400892.874	17163512.813	18352280.409	15349147.358
(9R,10S,12Z)-9,10-Dihydroxy-8-oxo-12-octadecenoic acid	453482829.753	293105791.195	438902896.947	332306084.514	305471859.298	321902261.909	379415878.532	423012939.845	401585418.835
Ethyl tiglate	89878874.721	53684024.394	88512044.111	48830971.973	43909903.371	39709606.906	36306828.049	43873112.430	50878553.181
CYCLOHEXANOL	156472009.924	38092226.342	62658671.282	160574480.613	104265497.616	30475810.975	144021668.028	148697277.840	191884174.627
AI3-34796	88126182.944	43829821.606	21604597.137	42819373.138	50722346.603	43308450.116	12724228.787	88982079.291	65857426.097
Succinic anhydride	22540900.788	17861809.447	41288931.832	72104604.401	113398449.762	85012993.613	72386298.030	20860591.163	44233521.730
L-Valine	617480992.823	13822311.494	313071755.210	40267534.843	123921284.483	170134958.761	109530502.415	229953513.986	85256553.154
Kaempferol 3-O-alpha-L-rhamnofuranoside	26459843.910	15757745.453	41252495.612	25840286.503	20635828.154	23895803.944	12634704.762	3310812.016	7989999.699
4-Methylbenzaldehyde	32951154.943	8542815.642	31228702.947	65961185.606	55162719.530	57669297.521	29991229.090	30910006.226	29686881.173
4-Vinylphenol	81408897.077	52325204.998	80857690.188	17022631.148	13925719.328	14262524.561	22418048.658	26374236.341	24526496.145
Valyl-Gamma-glutamate	7971586.210	6112701.711	11492450.693	86712813.040	86442159.164	84863182.959	17418509.384	15818919.559	17198693.114
Adenine	2197652424.708	1333593491.272	2253470090.167	1319658801.518	1208581829.689	1207084648.438	1435241195.692	1507957770.961	1518677060.103
Isoglutamine	31879013.857	124572565.297	269641144.684	1103763562.416	1037126429.005	170605251.041	273722729.838	248632351.265	223917213.182
(E)-1-Propenyl 2-propenyl disulfide	19376061.317	304827636.408	29679048.232	12978431.600	15030281.868	62914568.404	232395502.496	381072582.391	12802194.685
(E,E)-Di-1-propenyl disulfide	748507592.451	137945474.645	221990619.200	1867235422.054	1541983441.011	3039992164.744	335692947.881	375610729.119	378638173.896
Leukotriene F4	10388980.442	3417959.564	10576590.894	11531271.804	7143641.502	2093435.198	12495289.900	12215781.209	5618626.072
Tricin	66062241.313	40612942.708	60788587.224	18523478.710	18982145.461	16435698.977	56658802.689	60900490.012	60730408.711
PA(i-12_0_i-17_0)	405120	229465	437432	277543.	214436.	191238.	994836	109580	600318

name	S1	S2	S3	Y1	Y2	Y3	Q1	Q2	Q3
	73.410	15.937	81.757	262	488	036	1.505	59.233	8.423
gibberellin A7	43185670.379	26485597.560	41050329.971	26495446.872	28806115.746	27125703.140	15181220.127	15177344.489	15776156.455
Phloretin	11759787.597	6073897.082	12745077.834	65699978.446	51874730.037	57220904.727	400465.618	174878.429	248081.406
Stachydrine	45531093.292	23296605.411	51182091.384	74358223.786	64469872.066	62460342.012	199815208.680	219906652.798	222456689.098
Neochlorogenic acid	112602662.361	66248197.910	119121108.864	384502237.741	397351296.160	378512194.931	7555581.366	8013260.437	8008096.441
N-desmethylimatinib	29523372.394	15855753.514	33831579.314	844231893.260	688753250.456	2282113881.785	173839289.633	145343320.541	171946901.024
Ectoine	149833446.506	87899419.501	149342763.375	126723484.645	121704775.654	115575503.435	121970518.009	127189614.657	183031097.362
Tetrahydrocortisone	10171247.086	4841265.503	7705741.290	23816564.245	22662670.530	19528641.214	15910334.822	15790790.165	10562382.557
Isoleucine	414763796.369	237044664.608	383236152.810	491879322.792	555410342.830	433464009.331	230515231.677	225569177.065	229564500.721
Jasmolone	32742384.982	150794194.201	391778064.311	157698046.325	165666186.153	525359961.043	244267682.114	957275260.478	234393753.765
Thymine	338983366.216	198656729.397	336400454.040	6610213.633	24206110.184	22506709.577	249256705.328	72669019.141	253157599.454
Geranylcitronellol	243582976.463	138939628.080	203734226.404	24856484.645	17689056.536	19014964.895	21379655.218	21811694.667	23815314.838
(E,E)-Futoamide	43978253.306	19744712.881	28426662.602	11636033.088	9839954.748	9444152.351	10275998.972	11761252.969	10866034.236
Dihydro-dids	48852120.500	13245961.995	36526170.631	42918359.526	25601241.659	13138178.880	42591529.615	36121392.664	59978223.629
2-Dodecenal	36459930.627	14522885.244	35494568.760	8864938.701	6773423.328	7295833.690	14661179.189	15055413.511	4143944.908
Isonicotinic acid	259751395.625	156191464.314	260779773.584	925417615.782	528084080.421	520712964.848	288906835.919	306488360.110	297923905.167
2-Ethyl-1,3,3-trimethyl-2-norbornanol	100450226.816	27041761.575	127593781.290	18151057.983	31491883.074	14194768.942	23312661.686	9971513.200	10234360.249
[(2R,3S,6S)-6-[5,7-Dihydroxy-2-(4-hydroxyphenyl)-4-oxochromen-3-yl]oxy-3,4,5-trihydroxyoxan-2-yl]methyl 3-(4-hydroxyphenyl)prop-2-enoate	137940944.160	79206289.243	124275049.145	100462628.085	69803159.964	86209992.738	49906150.982	51588893.218	60726005.076
Diosmetin	16735753.824	9020128.605	12543779.762	8279263.813	6382536.388	6869995.250	15369984.431	17510633.319	18467551.921
N-formylanthranilic acid	97828096.965	49468938.982	78891009.091	53486126.682	43138997.977	37518333.652	35803750.766	11743340.193	12263853.827
N-Desmethyldiphenhydramine	84849273.376	43741262.135	89766929.018	29827516.629	26781071.187	31119316.658	68145752.674	73694207.782	72617525.461
Oxymesterone	69236178.221	48620434.093	73654474.645	42235559.888	35436781.046	36228813.168	30439177.521	28608144.385	29318503.961
1,3-Dimethyluracil	22725982.482	70280166.730	13737989.606	8401943.675	15916324.090	58796355.822	28222737.768	129665286.267	12209642.963
Icosa-2,4,6-trienoic acid	505012.611	107049.033	372232.956	12711782.626	12277088.627	11658053.810	2914330.128	2581502.912	2035234.291
(-)-Epicatechin 6-C-glucoside	453897.819	225720.921	421175.452	19351819.505	25798259.097	27458778.917	14274806.298	13182375.989	11791258.744
Diethyltoluamide	172095235.744	98595030.867	163257084.925	68890112.547	58521388.722	54459608.448	88466312.906	89350275.291	91568666.098
Stearoylglycine	7284351.073	2763300.273	7148256.519	33094885.648	13226388.622	10722491.346	6200860.596	7481581.598	15966606.568
Ne,Ne dimethyllysine	221566125.283	109891808.442	186905839.452	74454552.467	81037879.056	81922341.143	279124164.853	277991030.402	118809944.938

name	S1	S2	S3	Y1	Y2	Y3	Q1	Q2	Q3
1,2-Ethanediol monoricinoleate	51192911.624	27563197.716	46834474.101	7772799.834	8082657.157	6549591.714	16810931.668	15133349.686	15712473.345
Myricetin 3-galactoside	158790664.874	107549159.237	215920921.359	79511588.350	319833539.335	321549070.569	79522329.759	87294103.421	47973197.104
13(S)-Hydroperoxylinolenic acid	184440131.134	85048994.173	255375441.402	214918768.356	210858522.414	162644349.491	150095251.524	144076721.073	218639264.241
Oleic acid	13821150.228	2592902.385	4344376.407	23891591.415	18462337.727	20602013.682	25661268.400	13742104.647	12265756.653
12 Hydroxy arachidonic acid	8804643.941	4912684.288	10464398.703	2952398.023	54581396.543	38535979.437	8161946.329	8050768.045	7872113.915
8-Oxohexadecanoic acid	526025533.522	326858869.830	516020220.340	442706209.107	384586583.152	375321718.228	200061677.488	208460406.028	216189659.861
MG(0_0_16_1(9Z)_0_0)	84118738.248	63861388.037	95912000.373	29008808.213	35200849.709	29150720.698	30540818.264	33822034.466	33501826.441
4-demethyl-	665850963.082	683519250.563	1136564425.378	879906174.822	2194637969.255	2022430515.734	313825437.593	2099532921.924	1648021263.624
(-)-Epiafzelechin	7426385.967	4328584.278	5628715.314	69848742.084	64135115.228	57624343.342	2963125.379	3382873.317	2746087.215
2-Decylfuran	71942281.277	37098107.565	73093063.302	29803762.465	22298253.949	22916065.131	35956784.543	33385369.884	32819747.606
Volemolide	229009.740	53038.295	41416.886	31644051.164	26784973.645	26750738.020	1496140.301	1212361.945	1586371.952
Dibutyl decanedioate	53112470.044	30029761.341	52439715.019	66711.720	35230.255	244203.185	26523832.312	30280186.204	27643561.954
3,7-Dimethyl-5-octene-1,7-diol 1-glucoside	48588561.513	8409672.906	45676001.727	16192244.870	7741213.861	10315383.993	10826697.992	5464715.166	11089219.040
Adrenic acid	64565957.719	32274660.597	64871905.168	109018877.432	80871911.075	76598201.487	43150594.072	47255066.149	52416791.045
(2S)-2-[[2-[(2S,3S)-2-[[(2R)-2-Amino-3-mercaptopropyl]amino]-3-methylpentoxy]-1-oxo-3-phenylpropyl]amino]-4-methylsulfonylbutanoic acid propan-2-yl ester	94659192.581	34592832.890	89721054.586	19979573.278	43875373.554	45510033.431	20104686.067	49063591.811	63624219.302
Musabalbisiane C	44296590.815	17991135.957	19965612.257	4316264.153	3802200.352	1898158.622	6891395.396	2098257.323	4465153.961
Citronellyl formate	10391570.469	5221715.447	9145109.620	3246820.100	482248.296	2620849.076	5697802.885	5736892.804	4933660.069
Glutaral	75516245.978	215101838.672	72789102.812	102222142.482	152026360.819	107597978.223	255671197.837	202564165.303	116412078.541
Dihydro-5-methyl-2(3H)-furanone	42359605.500	27274173.993	228090851.114	73721600.966	43413544.788	315765776.743	32483863.614	112435346.956	136995640.486
Pregabalin	133115430.763	76107554.646	135193290.469	48989439.592	43758128.041	45998311.938	28237655.621	25846224.690	29244961.884
Isovaline	103636133.358	74547659.870	27490107.075	55273375.738	13099871.293	60809625.462	16356620.641	99865316.280	50840766.914
cis-Quinceoxepane	13774749.976	9558003.368	17433131.241	8215811.530	7389874.114	7463389.934	8613913.665	13083483.294	7436302.393
(1(10)E,4E,6a,8b)-8-Angeloyloxy-14-oxo-1(10),4,11(13)-germacratrien-12,6-olide	10282356.886	6154400.533	11262890.848	8449723.828	6607156.892	6194002.696	4037496.025	5452985.103	4131006.131
12-oxo-PDA	232104545.824	142667046.309	304207053.757	167262815.224	143998477.584	302792626.410	151122217.266	165273160.602	163628572.810
(6E,8E,10R,12Z)-10-Hydroxy-3-oxooctadecatrienoic acid	14324200.602	21053788.171	13788834.084	8043636.786	8649780.445	2096279.590	8462652.569	14593940.448	15026996.759
5-Hexyl-3,4-dimethyl-2-furanheptanoic acid	59775299.613	55167150.981	52859092.991	50874706.145	48051407.656	39356010.354	28492235.336	35091728.524	54947787.643
(6E,8E,10S,12Z)-10-Hydroxy-3-oxooctadecatrienoic acid	50400557.503	26882910.844	40789807.501	35741120.640	33780951.991	31731212.850	5968675.397	63783053.161	52994098.008
Avocadene 1-acetate	238246	141488	206861	523389	487832	419319	197083	209836	204565

name	S1	S2	S3	Y1	Y2	Y3	Q1	Q2	Q3
	2030.708	8404.184	6642.359	98.209	73.553	13.837	336.003	931.282	523.792
D-Tagatose	13943987654.769	10259639062.651	14475725768.941	11087647757.783	9874094725.936	9941634900.216	10806569464.182	11659409943.044	11212047922.283
Hexadecane	101724026.504	61231352.588	92104583.122	73756384.910	61395038.913	55266895.528	20280692.192	31151391.652	19878191.654
Astragalin 7-rhamnoside	106560359.730	14291055.683	8951976.735	10164090.891	2797245.793	3907135.468	15244981.959	24067472.829	15420112.073
Avocadynofuran	78237877.517	48691718.689	78725025.841	64560681.114	56133576.615	58256485.305	23197498.590	22290431.183	22889433.633
L-Lysine hydrochloride	44117442.624	54788322.966	46341620.052	16364479.387	15507017.134	13747290.084	38254547.896	45052347.502	37463512.368
Stanozolol	179390811.717	134664697.452	456712336.752	71716619.738	64155113.027	60833108.233	77482497.335	77808320.038	76459362.210
MG(16_1(9Z)_0_0_0_0)	61997637.070	34918981.610	138322712.330	38223573.941	33525894.262	3253463.004	9401097.853	9528723.943	9049338.651
5-pentyl-2-furannonanoic acid	116999633.895	71881159.107	113666719.448	5347005.355	6309848.181	4041705.179	21951068.910	22617391.312	20559461.690
DG(24_1n9_0_0_22_6n3)	50717244.662	32316048.128	53633218.058	35423069.487	14741187.724	10116871.786	25134723.055	15560966.153	34740724.589
Peonidin 3-rhamnoside 5-glucoside	59358181.363	16365519.754	45860501.327	11217006.675	7648698.918	14105353.836	129025264.807	22695871.953	15973419.540
Trenbolone	786515550.696	528099847.989	864260781.864	1380089185.264	1101578280.774	1185643162.993	23227001.650	13965216.642	11280539.510
Geranyl acetate	17719360.805	7675821.976	16806129.300	19660589.749	10772403.619	9859839.840	12122860.637	11430659.361	18170897.938
Octadecanamide	220096166.108	79628441.126	231704640.656	94358868.413	70098896.275	54752686.124	64567239.414	41879589.521	93407090.024
Cyperine	107909547.516	70478173.088	120271037.643	4295218.147	3145089.913	3581809.179	29539951.111	31945898.236	32224445.442
MG(i-20_0_0_0_0_0)	6978477.676	4142650.191	1724437.851	3637080.111	1764521.621	481143.491	8611983.405	9515074.440	9313951.025
Voacamine	3279918.739	872194.424	1590451.147	15212410.006	8470115.072	9120530.674	6263102.692	7417649.154	5658534.435
Saponarin	15957777.409	42615142.159	9997494.378	43174175.256	57164880.578	48980403.157	53230278.146	56902150.503	34319909.126
2-Polyprenyl-6-methoxy-1,4-benzoquinone	6012404.813	4246123.336	6290558.111	15781325.404	13407167.132	1338610.027	2549329.491	3855058.598	3143987.168
Dihydro-3-(1-octenyl)-2,5-furandione	34639447.090	16462765.836	23719122.601	5759603.018	5797756.160	5899013.603	45526756.159	47887969.028	48038102.648
FA 18_1	722642.575	308314.766	351643.481	11498952.603	5651268.036	1087501.333	3071459.931	3764096.059	6580101.833
2,3,4,5,6,7-Hexahydro-7-methylcyclopent[b]azepin-8(1H)-one	33258753.861	18514163.674	29674309.602	35514485.424	25340230.523	26853319.305	20468044.965	20617381.647	19314716.741
MG(0_0_18_1(11Z)_0_0)	5564604.125	12824563.540	76772888.784	19403230.301	8469932.428	6713422.387	12427030.802	7062650.000	12336313.906
Copalic acid	226528049.207	134015386.426	223420364.818	7152710.873	6351606.535	6795790.623	73496512.969	75279137.401	73253411.955
S-2-Propenyl methanesulfinothioate	62070824.182	9223936.736	34642002.813	6026412.327	5566249.450	5171953.915	25617227.617	13389364.356	34283882.647
2,4-Undecadienal	56655477.654	22927903.176	51638091.838	300959.696	243471.567	4937595.016	43176276.104	49641502.883	42932581.901
Isobornyl isovalerate	430877707.491	329109852.501	461871902.115	220915097.886	183454417.454	171753399.334	98423766.958	120804115.354	118730997.682
Nandrolone	410309	237424	391787	176678	150744	665260	466225	172822	161737

name	S1	S2	S3	Y1	Y2	Y3	Q1	Q2	Q3
	64.662	64.933	63.520	278.639	319.640	04.837	22.567	523.190	776.418
9,10-EOT	156654181.249	93484773.814	38993797.799	119174134.610	102188827.071	24970404.081	66704072.451	81952207.044	77487464.746
3,5-Dimethylphenyl methylcarbamate	28781215.265	16297799.829	23258797.584	13827764.652	15218285.075	5962592.423	23120899.426	26998867.425	24462382.371
Acrovestone	911847.449	754278.216	3135706.486	7941305.578	3023932.743	85653890.975	56015174.617	179696345.404	64569565.869
2-Propanoylthiazole	244110331.268	140987658.514	236373732.963	62273287.873	52627896.047	54084665.578	198071647.853	212942594.807	221166140.090
Galactitol	5689890.413	14198819.918	5951240.711	3165170.761	4986343.614	3101428.431	21267814.068	22912137.959	23765010.327
Homo-L-arginine	439905941.191	66283262.965	452582757.415	28685909.439	25955986.832	26434956.171	113945200.701	120710665.044	118396408.576
Peonidin 3-(6'-p-coumaroyl-glucoside)	204123138.735	142748705.948	585124727.557	17128373.920	9184214.149	12317389.527	45978994.673	68761409.987	515917673.147
L-2-Amino-3-(oxalylamino)propanoic acid	34799046.257	20246745.771	8882149.387	13256362.931	14590420.637	13389875.530	24831362.911	18352854.063	18434292.693
(13R,14R)-7-Labdene-13,14,15-triol	20279222.837	11816288.713	20287441.456	8440142.222	6998785.809	7595036.871	9265266.982	9550051.652	9296372.602
alpha-Terpineol formate	12260564.485	17056060.449	8539252.054	15929941.716	15709415.864	14585102.598	20573929.745	20426888.626	20238965.885
Portuloside A	149895906.725	138100025.818	170451556.668	3075133.315	2781675.587	2496735.011	37771106.632	40895098.873	36623327.905
Genistein	43565260.940	25679875.787	43641690.565	22072126.220	21879033.183	22299582.381	50583443.476	52571263.127	53241723.405
Flazine	258214875.907	102562640.988	320351706.585	28458204.615	15728782.580	21924081.040	30321653.755	33651588.352	46658056.843
(S)-Carvone	18835233.307	11933090.086	20836146.436	42718005.967	38320643.333	38717047.339	18945891.798	22246677.270	18860441.981
2-Ethylaniline	22811671.586	355941433.220	16481782.248	11325887.359	51750402.790	201258355.539	43244660.654	194682262.960	12549280.232
3-O-alpha-L-Arabinopyranosyl-L-arabinose	983567076.639	25709888.587	391940965.094	105076327.824	21104388.855	27919191.309	21132051.467	61238042.171	43511623.782
12,15-Epoxy-13,14-dimethyloctadeca-12,14,16-trienoic acid	735858.304	3116090.527	9063585.254	8082732.964	6692227.813	10801201.476	1520612.705	5219040.334	2086825.557
Acetaminophen glucuronide	7815381.239	4590798.069	6668364.262	15001959.547	12935636.149	14541431.178	28744960.859	4631722.459	11170944.118
L,L-Cyclo(leucylprolyl)	35559978.216	21507795.485	78909043.468	11649393.476	10862247.968	11005557.328	153762503.315	150711508.798	159564734.965
(S)-2-Aceto-2-hydroxybutanoic acid	638523773.233	343979466.352	576167029.308	1422626180.339	1330539076.352	1310272125.330	426391045.449	447697716.987	424210053.690
2,3-Dimethylpyrazine	301922825.103	187333673.696	312254409.158	227924406.018	214509479.429	213102923.573	256115349.304	280264377.629	263917976.309
Myxocoxanthin	236381092.025	30893520.034	48346078.870	27689023.707	47414103.059	25565716.064	78161176.422	26356989.187	95826124.730
Aminoadipic acid	282205346.718	97031147.638	281246423.796	307217801.004	288882450.644	288521482.654	340223448.515	385055629.638	380371962.668
Arabinopyranobiose	14940210.259	5016100.826	16309536.312	10714581.104	7011533.770	9154165.972	9781896.738	5212795.968	8340134.150
2-Tridecenal	18776203.605	12383287.746	40148556.051	5104307.412	4853448.519	4346448.708	5896493.310	6184849.764	15233895.214
Metaxalone	79735003.207	46838167.915	76812827.844	23401813.600	22414809.745	20600220.677	23873306.247	24422947.257	24719297.469
Carnitine	349575891.997	247937339.223	301509908.886	174937393.257	159950586.733	159145704.439	415513249.565	395470301.069	403445035.998
Fragransol A	546739	299549	467378	146400	429404	988155	300067	342112	352487

name	S1	S2	S3	Y1	Y2	Y3	Q1	Q2	Q3
	06.031	21.390	23.028	22.533	8.701	4.006	30.910	85.464	75.195
Indole-3-lactic acid	14727446.171	7394317.458	13172516.083	742553512.932	697582552.645	599881410.658	3607524.127	4124627.322	3878625.871
N-acetylglutamate	178169730.798	106045722.172	172480199.011	760596470.058	446894968.121	706832278.403	229824315.407	258697157.559	251634384.293
Artemisinin	26754061.066	14740648.632	27886754.182	19079960.972	18409398.703	17563395.761	7594983.891	9145687.114	7607581.146
4-Tert-Butylcatechol	48107725.175	38181179.962	51149983.174	9518123.024	7024575.457	6579052.462	19500584.985	21663157.445	21926526.909
Glycodiazine	38502749.982	38073339.331	68886642.562	38016003.686	41143103.494	37093727.289	34006279.073	33192362.910	30529004.548
Methylprednisolone	5024857.096	2141693.254	2461417.915	62969.126	37880.116	52334.920	1409205.811	1233533.416	1698602.491
5-Ethyl-3,4-dimethyl-2-furanundecanoic acid	116376630.568	71293123.899	119022984.500	78325663.953	67219661.389	17927722.774	65492652.612	75348525.363	69311838.104
Dihomolinoleate (20_2n6)	172396723.042	74728490.268	37402766.211	48480380.197	40433646.596	44878061.721	180718410.563	193960998.084	188267059.886
(2E,4E)-2,4-Nonadien-1-ol	67367518.722	39087920.337	73853069.343	26053110.464	20904458.754	21721454.618	26061362.157	8895952.429	8538759.765
N6-Methyladenosine	147408849.464	83795736.741	142177531.863	37337127.662	30357252.671	35303333.898	139374152.894	144344519.408	150336030.360
3-Pentadecenal	9535475.085	9983012.247	8115956.736	2551906.600	2043048.133	1857355.588	9922929.865	9603354.862	11258676.561
Dibutyl phthalate	228552434.307	131515629.428	279110504.729	88820792.465	80880654.686	87525696.477	113257934.053	137304536.952	115811003.214
Heteropyrithiamine	168720655.896	100120626.442	158965022.485	89621673.386	72071076.145	82903291.130	91814013.441	100880310.821	101246952.370
Tamarixetin	2912783.184	584436.516	891775.917	3774350.702	2661195.047	723548.048	20102932.148	20756432.876	20118082.287
Behenoylglycine	10366833.322	4680941.326	7239327.791	102700.842	312098.318	25306.451	507133.437	2162448.134	3256085.314
Leucylproline	32752942.106	22947178.273	35634000.834	11306209.163	10934227.382	7398776.992	33139071.693	38194607.259	34679343.313
4-Isopropylphenylacetaldehyde	5486067.651	1854697.019	3606650.514	12980152.225	12629344.126	11534475.801	6925164.729	8392970.863	7873841.436
Alanylglutamine	118141082.171	78186860.352	120257398.935	21256887.179	17890813.513	17725808.015	36223756.578	40018953.243	35673380.219
Phenylpyruvate	6693656.421	3698543.468	6645019.146	54769838.255	45732045.540	50764854.705	4410501.484	4180888.765	5119222.875
Arachidonic acid	9234527.003	5176947.797	8494063.335	6692477.239	4006881.081	2782057.768	6131672.746	2865637.316	4274168.332
2-Octenyl acetate	43884976.080	24369844.510	41975440.266	3507951.994	3376986.061	4748820.750	6241549.957	6446767.629	7083390.555
Methyl (Z)-2-decene-4,6,8-triynoate	26634815.207	14269047.232	28259087.462	2088204.636	2177637.013	2235839.394	23335008.892	28464604.936	26540331.311
4-Hydroxycinnamic acid	403826732.618	126010774.341	205598622.136	198495951.969	143438976.559	175459673.426	60232906.268	54321080.677	50084586.080
9-Methylthio-2-nonanoic acid	12947322.053	8453635.615	13123756.999	4640770.440	3854678.643	4650425.341	8716667.861	8556228.077	8522140.747
1-Acetoxy-2-hydroxy-16-heptadecyn-4-one	45321496.400	13181062.010	25499605.963	11648807.371	8429456.218	6882288.181	8520901.567	7765397.320	7655486.801
amorpha-4,11-diene	155780333.917	87630340.386	149568901.896	88144749.755	76325614.467	78533325.904	428909877.526	495804565.956	510069439.658
(+)-Ledene	40167208.127	24473979.368	37347299.660	30667743.285	29816875.556	21149532.223	130438716.843	126992822.860	135104392.711
Ternatin C5	39033000.683	10028980.524	25611991.497	9515291.788	6411778.140	2089853.533	2224054.198	2230709.117	6944634.380

name	S1	S2	S3	Y1	Y2	Y3	Q1	Q2	Q3
3-(2-Heptenyloxy)-2-hydroxypropyl undecanoate	12825445.528	2029142.887	8076529.405	60609440.620	26251506.080	18171472.935	15291564.067	16305757.871	29756027.570
4-Methyl-2-phenyl-1,3-dioxolane	14655174.905	9932517.101	15283330.469	1464009.196	1303203.885	1523369.510	8141298.326	2554053.034	6189494.330
Perillyl aldehyde	5447559.433	3886223.123	6961029.261	10953430.568	8139363.596	8423004.731	27073870.600	18422909.720	28359370.043
Maleic acid	27801529.151	17237971.550	24498462.200	143902213.218	123346416.890	129627933.930	184091196.337	198276297.680	190013040.807
cis-Parinaric acid	32167949.247	5381892.555	11351608.840	26555115.419	13847761.283	17760227.896	24180614.934	17343379.075	12960658.865
Shogaol	6238092.840	21651316.258	6547566.722	70178043.879	36939308.554	40641973.694	2870956.887	5617541.664	8653481.656
L-Glutamic acid	94588222.802	46927114.576	86674593.904	407641751.528	348904799.396	352748182.106	159749519.744	166569531.162	191115420.006
6-Methylsalicylic acid	49629023.167	39440649.834	26250630.120	8327649.633	20545726.788	8517140.832	8825025.081	9356607.111	11773051.923
Scoparone	12834608.058	8624342.017	13359354.635	2196973.624	1787112.627	1876422.944	28290389.429	28198317.886	27209217.626
Corchorifatty acid D	289011798.710	166364168.392	283354921.761	86287639.172	93617236.071	121192410.622	122568459.649	184869978.003	147866435.606
(+)-Gallocatechin	32054934.993	24602718.701	33737313.776	1574025142.185	1318468445.174	1362962134.006	24478501.614	32878520.904	27615559.403
3-Hexylpyridine	8502435.974	3799432.793	7393583.249	7196297.551	6277043.093	8897622.485	7304372.707	7780060.003	6160212.032
Valproic acid	39525523.210	80458560.471	367975609.460	83322323.883	69810989.004	17737316.858	22028772.235	264455641.612	76878562.806
(3E,5E)-2,6-Dimethyl-1,3,5,7-octatetraene	7812322.797	33110974.126	13464807.959	13071285.183	11021820.485	71112691.330	5734868.596	6176932.879	6465735.099
2-Methyl-5-(1-propenyl)pyrazine	13765975.566	5341908.661	8603348.008	4552934.651	7359368.530	9845032.001	4517699.908	4084498.418	3819630.441
Dalbergioidin	154243284.156	91656719.369	164281157.684	514164305.645	429887809.501	447194381.371	12390861.060	7908743.138	8489038.734
Nookatone	26209037.365	14366762.583	26088942.041	55026804.579	39741034.278	49418500.741	2951723.508	3633494.276	10536484.628
Xanthine	164465827.044	90126987.850	148337440.629	14344840.797	129112230.474	12208118.914	281891343.170	331455511.618	322245114.534
o-Ethyltoluene	34440383.871	27657085.353	63829272.873	72096977.762	5729007.096	5922206.110	84629489.315	85312438.778	16379158.054
2,3-Dihydro-5,6-dimethyl-1H-pyrrolizine-7-carboxaldehyde	116951072.916	69479316.002	117104477.476	7814092.392	7043113.749	7430567.041	30712526.276	33588297.666	38584382.602
Artemisyl propionate	946830578.973	562576046.840	910547651.381	434286039.953	393578364.467	393522000.712	579459792.673	562516404.021	536309835.280
alpha-Terpineol propanoate	42098690.799	24821173.605	64981252.566	15444290.128	10159640.832	19010687.240	18072066.653	18422036.877	24208948.986
Prolyl-Alanine	46936875.572	47561496.817	51322656.899	31638226.254	26998943.607	28710146.942	124507454.700	135593392.659	135655406.241
Isopropanolamine	6037551.305	3753664.803	5584891.373	35302944.683	30994735.420	29199814.613	95281817.140	114690095.573	97375793.952
Diisobutyl phthalate	58626377.718	15848389.185	60382893.650	7372705.840	28823741.552	31875348.377	35834251.298	43863469.687	24075738.785
Perillyl alcohol	216612181.525	70863107.231	128490558.878	109016023.100	296993448.707	307587615.761	107630151.702	105506142.072	108858979.986
cis-3-Hexenyl isobutyrate	12042019.309	52470302.699	13760921.613	218013670.531	213999247.563	244362270.987	132288238.280	163696794.882	183232463.036
N-LAUROYLSARCOSINE	24133170.417	3150985.316	24809991.461	2741187.825	2166680.125	2171892.451	3396198.981	5149452.628	5846443.068

name	S1	S2	S3	Y1	Y2	Y3	Q1	Q2	Q3
Phosphocreatine	109340932.406	524257708.034	129301415.453	544440982.228	498719238.271	517097347.495	623901822.371	540311308.922	604540528.031
4-Guanidinobutanoic acid	39743589.571	26004238.506	44346387.294	613550088.988	560188552.212	568240504.065	90709827.257	95328142.642	95887689.037
3-Oxohexadecanoic acid	22696403.171	13675272.157	21704534.875	7595267.229	5110388.071	6269875.178	7362598.961	7864239.340	9562253.242
LPC 18_2	188189981.076	67184713.943	244465440.602	6163344509.349	2730275900.787	4582843639.785	667754274.915	970183344.276	923338114.978
Danazol	13566943.610	7500751.310	11323924.136	2323413.264	443091.508	414590.214	3045884.196	1090628.219	3970913.446
Panaquinquecol 1	10354736.280	451249.615	8114561.048	33247662.723	3132482.227	3136487.623	7406705.390	2653623.282	19771344.456
(E)-4-Undecene	321010111.079	202524235.354	49375029.663	39720960.576	16821365.527	12755792.821	82929025.551	116103524.835	110852375.914
(-)-3-Isothujone	1659037.732	951142.915	1873327.216	28447829.741	23496633.145	53900101.596	15697038.105	19134209.873	2231780.906
Epigallocatechin	1297717.909	779648.826	1240455.047	276273011.999	256105292.929	252662829.587	8441693.965	8859593.556	9682931.441
Suberic acid	565152520.805	361934922.921	572903757.211	710387113.055	618684076.459	603430036.885	461136250.028	494703597.056	490182765.399
Ethyl lysine	325546275.253	202697330.181	314482259.392	15377780.761	14736695.160	22594392.012	16074039.159	18735618.244	16126033.008
Homogentisic acid	68050022.291	41455971.951	65850957.846	106094105.460	101168373.051	102010916.231	5208558.215	3935715.747	401379.531
Memantine	2649449.876	539325.855	1550557.592	2173763.585	1926773.099	2072109.604	4182142.189	3772735.040	5184494.835
1-Nonene	53346141.110	23141321.658	35114334.948	13688365.439	11988103.235	10962986.686	16508439.360	17952031.131	16778368.767
1-Ethoxy-2-methoxy-4-(1-propenyl)benzene	65919562.096	39184632.351	67128756.780	21398537.792	27439304.533	14999828.807	22716088.256	25728722.138	26253044.800
(S,E)-Lyratol propanoate	87571907.921	52360668.949	125873420.297	29522809.012	15757997.053	36099298.549	31948791.829	34756271.697	48675068.596
2-Amino-4-oxopentanoate	24091733.628	20036701.890	20565750.238	27150652.886	25943271.196	27807460.471	76447430.828	30647641.330	40165366.525
Dopamine	48026149.251	29986145.423	54045757.657	29539050.347	2598024.394	2276881.499	14826690.505	19312621.324	13173365.114
Benzyl ethyl ether	34552356.844	20001725.754	31617702.660	6039823.825	5516170.753	5982828.447	20910773.588	18799495.047	21395629.698
Biopterin	27343925.991	19490363.704	33053970.559	11956376.324	11110728.136	11098559.683	28987461.935	33627452.217	30877919.118
4-(3-Hydroxybutyl)-3,3,5-trimethylcyclohexanone	56694943.360	31580737.544	49881096.119	25070540.956	17910119.229	24263212.616	25935931.219	27673555.407	23202794.750
LysoPE(18_2_0_0)	12738006.511	8677707.263	10562746.054	397981564.963	144665964.465	181850864.619	115038946.980	80777167.102	66684650.819
Cardoltriene	3104161838.557	1766409099.886	2713069505.363	53872542.501	45300862.046	45697510.314	264841564.657	290644460.320	274500080.634
3-(Methylthio)hexanal	37334842.656	18683340.349	11872376.613	24067369.523	17687596.750	13278350.793	11796750.535	19128800.479	30078317.372
LysoPC(22_5(7Z,10Z,13Z,16Z,19Z)_0_0)	44856907.735	14774516.221	48768220.304	25563239.092	19276096.542	45237945.750	18910958.668	27033730.454	21076919.946
4-Hydroxyquinoline	1338140239.626	783862105.645	1249638781.516	96396271.476	88317785.454	84478510.738	239720480.415	236400746.614	220240938.686
(-)-Epicatechin 3-O-gallate	2777690215.66	1782120801.57	2783532708.95	3613454555.46	4369474087.01	4040497078.54	147225762.102	123785778.756	117228110.153

name	S1	S2	S3	Y1	Y2	Y3	Q1	Q2	Q3
	3	3	5	7	7	4			
4-aminobenzoate	198096<br>924.066	116908<br>868.750	191660<br>597.382	571485<br>29.816	470933<br>94.341	499118<br>33.912	408205<br>162.731	459781<br>075.749	563272<br>217.083
Ethosuximide	575571<br>64.126	327553<br>75.484	535712<br>29.555	584827<br>29.211	510261<br>16.964	568812<br>24.364	117889<br>841.578	143521<br>599.148	144603<br>458.452
4-methyl-5-(2-phosphonooxyethyl)thiazole	900899<br>3.869	547053<br>7.769	880091<br>3.533	305601<br>18.386	251926<br>64.112	237596<br>79.819	313187<br>74.391	336579<br>62.136	304062<br>98.507
(2E,4E,7R)-2,7-Dimethyl-2,4-octadiene-1,8-diol 8-O-b-D-glucopyranoside	155180<br>35.609	942029<br>5.597	536143<br>97.141	217433<br>23.017	195289<br>56.796	198571<br>41.027	323274<br>05.551	336357<br>33.903	325558<br>55.069
2-Methyl-4-heptanone	120540<br>93.371	123183<br>02.138	130988<br>47.958	473724<br>31.860	288859<br>12.065	141284<br>91.631	197273<br>31.896	676435<br>85.774	183013<br>76.944
a-Methyldopamine	171105<br>606.624	103512<br>953.179	168134<br>257.119	489762<br>59.494	443080<br>70.385	442809<br>15.272	190634<br>337.954	229937<br>978.124	205864<br>007.911
N-Acetylleucine	628028<br>67.749	405633<br>45.555	860854<br>84.919	175917<br>74.401	134661<br>44.922	965362<br>6.171	270785<br>72.691	307035<br>35.767	270711<br>48.867
3-Methoxytyramine	235225<br>97.015	130478<br>52.605	189634<br>59.515	934547.<br>916	673410.<br>966	482016.<br>060	112997<br>72.635	123625<br>79.886	937945<br>7.521
1-(2-Furanyl)-1-butanone	137094<br>064.735	950303<br>68.114	160060<br>195.785	551726<br>795.218	488929<br>812.769	505928<br>760.502	174184<br>23.723	146636<br>52.680	169350<br>01.651
4,6-Nonadecanedione	366971<br>48.262	150433<br>75.168	241699<br>99.647	959375<br>5.387	760023<br>2.202	710915<br>9.175	112333<br>47.407	118417<br>46.970	112108<br>45.816
Norbicycloekasantalal	192678<br>68.918	114673<br>91.599	363513<br>51.559	387591<br>58.937	328735<br>16.766	346203<br>00.585	245152<br>89.307	225633<br>58.456	237706<br>04.151
Alanylproline	181891<br>17.332	117540<br>81.760	177704<br>91.240	175107<br>00.932	163135<br>19.519	206190<br>17.471	151182<br>94.545	142201<br>33.553	133808<br>05.987
Pyroglutamylglycine	173795<br>95.252	101763<br>63.107	162298<br>79.327	105463<br>56.210	893726<br>7.967	675228<br>2.463	107540<br>15.096	107438<br>12.710	116790<br>41.299
xi-Tetrahydro-6-propyl-2H-pyran-2-one	374149<br>17.587	237868<br>41.013	355413<br>45.835	125469<br>66.974	100376<br>62.310	987954<br>3.011	259616<br>99.415	289274<br>35.082	291856<br>81.750
L-Arginine	816870.<br>360	106137<br>5.572	116892<br>9.716	267940<br>99.317	232115<br>04.549	235183<br>77.888	228619<br>2.582	180292<br>0.745	395059<br>5.986
1-nitronaphthalene	868858<br>0.952	124962<br>1.418	244015<br>9.854	156688<br>01.309	868523.<br>796	335279<br>0.077	411671<br>7.040	112162<br>2.684	302051<br>3.583
p-Menthan-4-ol	466846<br>16.975	294622<br>78.565	421601<br>90.756	182265<br>4.069	173473<br>9.236	177716<br>8.876	906494<br>3.171	901458<br>5.259	952592<br>6.601
Nerolidyl acetate	175075<br>23.442	100793<br>11.341	228690<br>49.015	173927<br>71.715	130859<br>21.000	137925<br>60.860	123886<br>40.898	140730<br>73.305	122675<br>15.439
3-(4-Methyl-3-pentenyl)thiophene	449271<br>69.166	203303<br>14.389	367950<br>92.353	846643<br>3.393	559878<br>4.264	745903<br>7.036	181206<br>6.112	223139<br>8.081	205126<br>5.249
TG(8_0_17_0_10_0)	768715<br>958.432	335978<br>752.708	800625<br>260.406	277662<br>89.857	393836<br>17.359	340064<br>65.528	240105<br>376.223	451806<br>137.960	263493<br>408.030
Lilac alcohol	102411<br>06.266	506528<br>1.221	707982<br>7.487	182316<br>02.422	152032<br>05.161	100895<br>76.673	604828<br>9.336	140896<br>36.936	166897<br>82.660
Linalyl isobutyrate	471288<br>76.334	288891<br>84.101	461166<br>01.743	705153<br>8.919	526401<br>8.894	611818<br>5.777	205239<br>64.822	210409<br>01.833	194127<br>37.968
Mono-(2-ethyl-5-carboxypentyl) phthalate	357091<br>4.275	136968<br>8.383	749248<br>2.432	213397<br>1.293	141002<br>2.142	144183<br>4.692	307772<br>15.979	730382<br>6.773	289776<br>4.195
2,6-Dimethyl-2,4-heptadiene	519529<br>83.364	293596<br>11.401	887464<br>11.292	746511<br>6.969	774790<br>5.970	550985<br>8.295	415983<br>0.358	416112<br>6.906	421924<br>3.469
7-Methylxanthine	305502<br>084.919	192845<br>809.517	313379<br>391.631	113352<br>745.115	116808<br>430.929	106417<br>601.880	103731<br>851.366	114547<br>184.853	127620<br>887.268
L-3-Phenyllactic acid	249952<br>28.356	962255<br>67.310	168089<br>491.815	415044<br>29.351	377696<br>04.678	911350<br>29.786	448998<br>81.668	187481<br>89.311	103423<br>34.074
3-Phenylpropanal	685070<br>0.306	956012<br>8.805	764582<br>3.744	224069.<br>041	292919.<br>397	264544.<br>051	208477<br>35.273	202914<br>89.880	203246<br>31.104
2-trans-6-cis-Dodecadienal	477796<br>13.240	165914<br>67.470	276873<br>64.587	382565<br>71.759	937992<br>6.587	937137<br>8.485	526252<br>94.223	327859<br>20.745	510402<br>67.909

name	S1	S2	S3	Y1	Y2	Y3	Q1	Q2	Q3
(2E,4E)-2,4-Dodecadienal	71219668.741	7868441.947	12567613.934	9438606.587	12809250.078	15337292.767	13452431.395	12759046.711	15724668.465
Homodihydrojasmone	88898275.565	58462976.232	50087816.880	74730179.602	13779816.676	71204395.603	86215331.479	52413743.636	22533527.189
Methyl hexyl ether	8287890.182	5033219.359	7250177.916	2341933.542	1600511.823	2044539.009	1924989.806	2592883.975	2865590.107
1-Hydroxyvitamin D5	55340788.832	14215386.870	70765820.711	44728471.597	41343728.830	34894975.818	34631252.341	19851530.607	34375611.189
Cathine	54228853.525	31225914.181	54436672.887	27942108.157	25201826.012	27113135.301	20015059.250	19842830.453	20056972.953
Pulmatin	36147095.398	22710734.270	42747495.204	6325148.961	3537710.395	5231961.129	7555041.040	9095973.477	8598252.507
xi-8-Methyldecanoic acid	68706929.080	10950504.610	93030594.947	21862999.838	6792736.724	10704474.086	25450686.805	30649989.035	21274269.001
Punicic_acid	30727691.075	9970474.847	32815938.711	5546424.926	2453674.892	3657379.409	11757283.413	13791256.412	19714498.828
Methyl caprylate	15139924.179	24060630.143	54778294.410	3965100.270	3313787.487	5181143.966	2410319.144	3005428.388	10284081.736
10-Undecenyl acetate	2571386.874	1343974.371	2210125.122	14613309.637	12084701.198	15167502.859	4485433.383	8304257.749	5197998.523
beta-Carboline	3109561.388	27437438.526	3433619.537	11935320.185	8285182.722	11620927.446	31447641.744	31667535.276	29466047.966
(10E,12Z)-9-HODE	2331309442.638	1391529959.131	2348724508.247	95767111.637	98965236.355	99643056.340	764142719.598	779903771.618	807115987.659
Prostaglandin H3	1737142.101	884827.709	2171083.382	8238546.973	6626557.956	6851866.884	709848.378	1108766.689	810808.483
Harman	32443665.679	19959978.699	35477310.421	13905818.945	12146941.813	10607579.169	39818735.483	48001959.559	43650615.779
6-Pentyl-2H-pyran-2-one	16446477.548	13618827.620	13176791.407	7893904.373	6327359.499	5794007.745	12466485.697	6097531.027	12437148.111
2-Phenylpropyl isobutyrate	7314746.060	7064004.859	9906344.254	20390224.447	18283603.164	18013367.199	12693277.788	14648204.658	29328386.967
4alpha,5alpha-Epoxy-11-eudesmen-3a-ol	19226144.391	10839740.205	17785037.865	1668778.082	1503945.752	1704864.172	9819566.455	9984418.363	8539413.048
Betaxolol	2420653247.688	1441153324.654	2450853925.796	232753771.174	190939633.590	198780858.433	306233198.100	313402465.493	320921960.452
Butylbenzene	80794424.401	13978379.570	27567360.613	36017857.633	44791690.750	45596389.492	18286871.457	21934872.326	42181394.069
Tetradecanal	15135757.362	2467253.417	17332829.705	7397079.841	1520162.409	1250107.168	2948020.427	11784265.459	12521818.288
Ethyl octynecarboxylate	21550609.392	13977737.767	21167334.033	36085557.339	32099281.985	33113316.710	20214338.614	20741942.842	17920739.451
1-Phenyl-1-propanol	49786359.820	38090719.349	49862695.504	4166176.925	3735335.230	3583181.502	22009643.583	26834340.190	31661756.533
Oleoylethanolamide	22203691.420	8309557.623	18530126.326	13461085.741	9671510.413	9977772.195	15966115.855	10252772.437	12556141.450
Prenyl glucoside	1794768.766	1232408.334	1698736.654	14378444.916	5606626.380	5315609.054	10491519.697	6446773.821	6758189.735
cis-3-Hexenyl pentanoate	14421482.468	8806179.723	13581184.835	1772853.620	1616446.830	1467608.393	12493981.184	14969033.566	14525084.609
Dihomo-alpha-linolenic acid	60574075.771	37400001.246	123323109.085	31503358.164	30291110.376	30603708.779	51957520.287	58898823.555	49754988.181
12-Hydroxy-8,10-octadecadienoic acid	3268626.096	1431244.797	170181691.649	67022668.289	37507450.396	53532647.132	9630549.794	15465801.190	12085596.470
Quercetin 3,4,7-trissulfate	173540	111306	147126	657356	103574	700142	356842	276845	382510

name	S1	S2	S3	Y1	Y2	Y3	Q1	Q2	Q3
	99.917	94.782	04.118	82.255	15.761	66.411	4.031	5.040	2.618
5,8,12-Trihydroxy-9-octadecenoic acid	66325064.639	19004027.514	56024952.190	253617986.105	106129242.286	146636601.173	83319044.347	99063460.656	157305574.727
Estriol	94880541.157	56575522.854	84503661.536	16223437.523	18225718.088	16556010.247	25015396.555	25142248.714	27452064.244
Glucosyl (E)-2,6-Dimethyl-2,5-heptadienoate	3936994.198	106102.381	1233648.981	48541687.297	17224812.188	18817678.964	4848098.679	6676823.085	12569951.491
Acuminoside	11876901.804	5968224.228	7576541.478	116383133.853	41591757.906	27321826.105	9645741.134	12110524.990	10865138.817
alpha-Cyano-4-hydroxycinnamate	11590714.003	24236187.951	12019938.289	28919252.603	23686820.116	22090503.670	15369468.075	9052642.107	13473071.456
Castanospermine	51482314.908	21545096.031	35944147.364	60215811.941	45566447.118	48472750.860	13088503.316	33579442.512	16529288.393
selenodiglutathione	70032834.152	28145048.288	12563963.414	129911332.162	72652357.466	116843762.870	4894967.144	5357960.945	3189040.210
Pinolidoxin	13266667.437	8068936.657	24284479.218	22468586.143	19675912.173	20029336.061	12776161.938	15980375.486	15876860.156
L-Aspartic acid	2805052.931	489130.022	535744.698	84917282.155	67535698.398	75495406.932	5067711.783	14656822.350	10268611.394
CE(7M5)	10875872.039	6548419.432	10416232.578	10930849.499	16101596.035	16316247.004	20065228.655	2904571.968	6797889.246
Octadecadienoate	24523447.442	7007262.289	11691738.915	16635654.970	13690611.204	13022528.909	28304722.605	23686727.838	31499783.417
2-Hexylidenecyclopentanone	34654716.684	19585885.640	31762821.763	8847861.079	8509398.210	8203111.915	13686573.671	12880885.780	13673059.024
(R)-Amphetamine	1141152354.394	669228215.967	1062039008.510	542032188.867	463480027.464	511627945.487	748506740.444	784834586.129	738503070.456
Phenylethylamine	7743655.625	39047145.657	24994116.811	69989754.244	57676060.028	58949197.974	17259456.751	20299644.676	19431193.947
(1E)-1-Phenyltriaz-1-ene	96441817.565	60389969.028	117086444.154	18857322.949	24520962.678	25798210.753	33246825.118	37175977.732	35696052.771
(S)-Nerolidol 3-O-[a-L-rhamnopyranosyl-(1->4)-a-L-rhamnopyranosyl-(1->6)-b-D-glucopyranoside]	4239777.652	2681093.558	5154749.490	121597039.755	109622645.619	65682909.689	9268766.757	14145795.104	5116936.312
2',4',6'-Trihydroxyacetophenone	67195055.093	9160794.616	62288960.081	1846071.260	1437489.281	1295966.296	30335960.614	36149964.991	33171305.681
Diallyl Trisulfide	18231305.160	7043414.372	17083136.851	9344044.454	6053136.371	9909943.100	22793294.490	25385335.058	26980866.542
(-)-cis-Carveol	87491350.865	13563148.708	128340330.262	124589790.293	48454949.525	45969455.776	2911829.204	3300176.027	2934115.026
(2E,5S,6E,8E,10E)-5-hydroxydodeca-2,6,8,10-tetraenal	3282761.399	1967371.549	3505095.809	45958729.921	4001880.661	1961669.320	5521658.794	7752114.447	6062301.952
Pendimethalin	34532770.628	18859458.962	30331797.631	61095646.365	49030644.876	54057310.077	3080149.653	4072687.700	3701784.172
3,7-Dihydroxy-12-oxocholanoic acid	143318788.071	86147373.026	143854458.152	99308890.213	87999509.855	93107990.431	37735995.446	41387750.666	19555116.107
Boceprevir	2388445.309	534932.270	1436573.447	136691566.293	45894184.260	155270207.566	8505697.477	9957132.364	14253361.034
Ethyl 4-phenylbutanoate	25650627.044	12361334.617	33926918.824	4797310.189	2227386.659	2274202.828	4552108.748	4921039.444	5154136.491
L-Tryptophan	8910162.938	9993890.457	14481046.259	1461380979.936	1287769135.507	1141708387.196	8461535.263	8984735.143	11334571.876
5-Oxoavermectin 2b aglycone	18092735.708	8991763.646	16288157.908	1726008.798	1077960.488	1245150.219	7055679.795	7940209.467	7166735.933
Chlorzoxazone	606234	345880	547693	686467	530027	569320	394489	405255	405589

name	S1	S2	S3	Y1	Y2	Y3	Q1	Q2	Q3
	72.707	52.415	92.120	65.496	04.223	19.881	97.316	13.194	71.953
Propylbenzene	15939137.449	7894784.275	13099181.817	134062338.735	114030337.009	124206969.235	19397786.896	16363919.133	15457100.142
11b,21-Dihydroxy-3,20-oxo-5b-pregnan-18-al	198238146.614	114443759.946	186755145.403	32861628.007	30902170.636	30456117.167	78530978.136	90219403.446	84127517.265
3-(3-Methoxy-4,5-methylenedioxyphenyl)-2-propen-1-ol	51641093.608	29258871.407	49317922.298	26023781.909	20150220.339	19223424.293	29626436.207	48844022.159	52101724.100
gamma-Aminobutyric acid cetyl ester	584029.652	477928.885	37135190.021	59398121.108	23730225.973	26010523.159	22852843.507	23874675.986	55845860.687
3,6-Epoxy-5,5',6,6'-tetrahydro-b,b-carotene-3',5,5',6'-tetrol	34067931.526	3893882.711	6819230.352	21476421.396	17836127.941	9325705.040	9118449.456	44680553.190	61088504.449
aphidicolin	36261131.367	19534713.520	30471657.415	5210502.713	3107641.123	3090481.182	49521226.441	58061193.412	55525149.129
3-Pyridinebutanoic acid	84974856.678	14766588.176	23186136.929	3672959.470	11698670.974	13259352.109	26600520.578	20258804.901	25825079.129
Creatinine	6221383.965	39014469.199	6000062.631	37012309.004	36447718.231	33664211.069	97565396.711	102930389.428	108109452.266
4-Heptenoic acid	77088505.755	43584004.667	143658667.627	15825978.509	13186491.550	12038025.426	109155255.591	128523427.505	129407301.152
Ouabain	84304479.401	14774179.622	59970273.682	9600630.458	8323750.704	17699566.983	11502499.589	18761336.281	30250393.494
2,10-Bisaboladiene-1,4-diol	29051116.898	19431255.693	26610942.583	20460549.737	16769289.266	18267068.190	23771923.597	25069928.814	22032672.600
N6-Carboxymethyllysine	128608564.861	76104403.439	118854450.161	41652371.445	78335238.223	37583196.718	44400375.503	107897996.971	66095519.311
Margaroylglycine	398840.220	874591.883	49783.677	22200779.919	23745862.083	20566392.504	1432615.444	1284864.503	1120923.372
LysoPC(16_1(9Z)_0_0)	11851461.686	6973016.193	15752908.877	128148319.942	116429749.747	112252686.583	26410133.634	28279722.675	23372715.431
LysoPC(18_3(6Z,9Z,12Z)_0_0)	1543801.268	191261.886	298445.667	39307595.201	56325863.866	40581482.224	6354798.468	6782314.648	8277854.550
Diethyl phthalic acid	33820301.172	19504637.066	37535009.083	13762112.687	12757081.341	14977264.225	18315723.017	23155066.654	18294648.857
3-Ethenyl-2,5-dimethyl-4-oxohex-5-en-2-yl acetate	7014696.241	6240244.051	7025505.661	13021631.084	1636405.869	8416035.541	6744474.792	1955838.677	28072506.621
Momilactone B	22812193.753	9027601.815	29829688.338	28582443.466	10884013.923	16474919.416	14098906.019	15481951.563	49361989.604
DG(18_2(9Z,12Z)_20_5(5Z,8Z,11Z,14Z,17Z)_0_0)	64639528.669	21076400.380	95173100.368	11089634.537	6870642.872	4857771.655	7989816.091	11700354.705	21879639.247
LysoPC(0_0_16_0)	78738843.265	20861552.214	109102343.277	137640743.766	313855726.671	253586790.906	92206752.444	22098448.596	149541652.592
Vitexin	17368066.335	15958743.526	23805559.402	24456338.037	26671410.801	15400258.705	15764799.891	28041163.658	15498125.058
Aprobarbital	60271965.244	37510943.327	50706408.673	18566835.401	14215959.473	22840965.292	75043286.451	74572338.333	66781672.876
Citral propylene glycol acetal	44607172.449	26528273.192	10148561.114	19200368.509	18392280.981	17037794.743	28751316.020	41914802.108	30290792.621
Cytosine	114038903.502	68150334.305	112184781.383	74313462.870	62805487.118	63630467.164	155284330.139	153816809.621	152769570.850
beta-D-Glucosamine	2100800.350	1911250.399	2413150.677	56870852.603	83634494.507	990214.890	3194442.874	65503626.131	56861008.032
L-Histidine trimethylbetaine	113776948.047	67911045.445	113314210.060	206472374.828	189965917.089	152275274.418	49237476.378	52750094.166	51820304.745
1-Methylxanthine	2479987187.802	1140284815.944	748990143.873	529701054.343	465448301.419	483058703.700	634458002.149	643852841.508	674123715.655
Cheritamine	570372	989712	501180	138023	567496	145269	652543	451845	241586

name	S1	S2	S3	Y1	Y2	Y3	Q1	Q2	Q3
	785.849	41.701	544.742	384.764	30.322	030.059	83.676	71.448	75.778
Tyrosine	95075814.258	14788483.570	97592134.864	380156614.352	315592257.666	323556674.344	37565092.579	43641072.793	63172740.189
Ethyl octadec-9-enoate	461572.614	5229253.424	187304.689	2765052.068	2399569.105	2534549.206	6458306.041	8154033.866	6941445.221
beta-Cryptoxanthin	125047342.683	289880454.524	413722873.761	191939288.614	43030285.531	198911767.958	213014312.879	80386745.081	146688212.157
7,8-Dihydroneopterin 2-phosphate	46183193.371	31176537.447	57838668.291	101741076.714	83674842.493	81259107.149	18113853.724	21982170.213	18259422.403
(-)-trans-Carveol glucoside	37741907.639	17837234.678	28633014.788	816883493.804	733204165.756	684591065.900	134187234.128	107460302.060	107931772.384
Lucidenic acid K	19981368.247	12792723.560	20973824.968	10511999.719	72026440.014	22347198.599	37970888.326	14268411.348	41366538.986
SFE 7_0;O	363698810.356	200802540.357	319388173.800	342229184.979	289774269.103	295840882.821	203215502.040	227794844.069	202231966.946
Androstanedione	297782361.672	187789910.693	204512586.971	126714113.195	107231039.275	110346451.057	188504859.589	233299057.153	188583432.534
SM(d18_1_14_0)	456981934.083	202609390.335	418950171.993	73076286.451	134685161.967	130840485.066	212385129.537	181885849.287	283424376.471
aminophylline	21945661085.762	14011020986.667	23237311808.948	1366657282.428	1238297586.812	1223846172.831	2174904694.860	2309295320.989	2088086233.221
Methyl 2-octenoate	83451938.390	50236222.712	78499565.479	150924955.860	134240032.780	136174905.673	1869266.043	1924860.551	1740706.156
Roridin A	25963967.210	15102317.632	65528133.399	10938972.769	5364467.293	5497065.033	2446425.243	8001207.100	11530384.327
Alpha-Linoleoylcholine	170954340.032	97998851.157	199872106.847	20815286.690	19061608.939	5691751.232	116677888.069	104346059.390	95199626.183
Tulipinolide	22672267.078	10303029.076	19621438.906	5397537.810	3889530.438	4101350.024	4679845.694	5186955.942	5253017.044
4-Hydroxy-4-methyl-5-hexenoic acid gamma lactone	46067653.230	27451711.549	45021318.794	9123395.975	6960287.390	6477639.561	16944590.006	18702113.602	17694320.805
Niacinamide	19025268.744	12372068.443	19887551.291	129441872.795	71872484.284	83993240.867	70960700.112	79766545.091	74748801.987
Soyacerebroside I	209549.672	248719189.654	23531080.288	42928534.348	10305293.964	40129938.591	49208917.148	51686045.157	95252704.323
2,4-Toluenediamine	4468745.059	2638574.752	4517460.607	41624866.525	35439897.566	37315793.355	9741657.195	10501096.546	15511044.472
2-Methylbenzenethiol	23396624.610	13577282.276	23044808.734	4998750.216	4337731.635	4301639.466	3244913.504	4392715.953	4393183.871
Cefpirome	14281730.183	3110695.203	8969639.694	25888344.625	18023115.840	24781396.815	15902740.614	17236039.196	19739572.027
Homovanillic acid (HVA)	1704582.264	56618532.141	1153831.092	695803.669	13186072.083	25165671.542	24633139.438	83321668.688	837563.013
3,4-Dihydroxyhydrocinnamic acid	2352872.865	2231542.073	24684204.589	9370222.825	6725743.183	7598314.139	6743888.885	8990746.288	7445556.734
Neoxanthin	145556904.004	29629355.955	54709192.328	47822082.628	19977721.884	8457506.165	43761184.087	14842107.242	36243445.110
2-Heptyl-4,5-dimethylthiazole	5972528.942	2575556.662	3065127.802	2561103.169	2521014.921	1763093.056	16451030.402	15215012.931	17170241.243
3-Hydroxyisovaleric acid	127401840.221	76024216.691	121491285.947	142384022.831	113516367.654	115782719.068	84906657.539	98042405.501	89834950.717
Asitrilobin D	68399981.594	63267437.612	33507917.581	12635334.512	10960959.899	4948683.575	49914988.556	17616628.645	46785558.155
N-alpha-acetylornithine	559663515.160	332696143.316	562952211.406	163037077.033	211908566.790	225886784.903	641739884.208	581596930.369	540237179.429
Leucopelargonidin	188998	122961	191259	147261	133536	915596	123929	161074	165288

name	S1	S2	S3	Y1	Y2	Y3	Q1	Q2	Q3
	29.248	01.866	00.958	48.379	08.704	4.916	26.105	67.601	19.235
Sagittariol	111038264.359	65087085.574	112874816.407	10105639.420	13246134.244	31855758.799	80984459.734	85174050.587	79049792.481
2-Heptadecylfuran	684895667.951	388918657.115	649763568.162	366920243.382	348686636.115	326095581.369	578940160.974	715244186.145	598611491.391
Cuspidoside	10077393.521	4789448.886	8469163.731	106253763.709	97410868.633	87098453.193	38687704.855	56429373.588	53926354.712
5a-Cholestane-3a,7a,12a,25-tetrol	43403663.224	27703780.585	35420193.556	14041215.342	12605993.539	12632035.554	43032235.510	45238712.001	50634244.002
Tetrahydro-5-isopropenyl-2-methyl-2-vinylfuran	72809984.981	40641524.094	67920767.954	27073239.121	25891863.922	26276571.069	185171018.747	192754390.105	186524678.609
L-Theanine	62201975.246	43337659.409	68334235.960	10819947576.768	9399033174.397	9959768482.850	18384331.618	17346499.179	15818532.124
Dextroamphetamine	5951970.266	4200199.388	6857497.187	2447320.421	2214169.064	2087299.893	3935642.659	3342930.761	3861221.370
Nafcillin	148180385.832	88534617.214	153649592.346	72389448.739	64620869.818	65068698.984	83433807.839	83335099.790	77897807.091
Octadecyl fumarate	12099585.577	3964156.075	7036412.295	5810874.382	4722561.229	5907475.290	3204615.906	4632640.519	3202591.689
2,4,6-Trihydroxybenzophenone	132712216.285	67601606.157	141144191.638	27729397.583	20075018.028	17612235.537	24650687.254	33530146.258	26296920.426
Methylpyrrolidone	290474976.752	152093214.255	257386761.949	24552145.480	19096757.652	24423459.683	27513523.346	16684173.912	21107415.486
cyclic N-Acetylserotonin glucuronide	4204916.851	19009992.320	22302022.145	13120378.960	37040896.308	36443266.864	10986635.495	6270729.046	2965773.590
4-Hydroxy-3-(2-hydroxyethyl)acetophenone 4-glucoside	795541737.941	493992249.646	834294803.432	14934327.669	13349137.879	13497948.620	43909646.833	45147611.384	46153314.297
(2xi,4xi)-2,4-Nonadien-1-ol	33986739.755	20978196.591	31255148.652	8449208.165	7355733.365	6357108.221	11401545.715	10860758.662	10059150.467
Vobtusine	710421.741	318381.824	708162.198	22285181.845	24192734.109	18687907.534	5512719.788	6846589.751	8723295.669
Avocadene 4-acetate	12977984.440	528174.960	629880.868	15714706.826	14742284.420	4670433.048	27311237.607	11752131.555	10212333.181
Dihydrorhizobitoxine	47350940.741	28964307.176	41749861.351	59444809.897	57112381.879	53425722.150	77532349.887	82641511.513	83471243.234
Germanicol cinnamate	166959658.052	46498799.695	193663955.916	112375219.907	44095020.300	70892984.852	91063301.761	84074285.889	131712632.553
Nonanal propyleneglycol acetal	4847112.053	2601676.467	3765287.371	39360001.137	35084403.988	34454960.280	13336681.167	11209167.657	17120848.678
CoA 4_1;O2	4705875.748	13610334.178	19756119.970	71027188.456	63180712.338	26574277.822	19115896.821	22579005.236	27664800.127
(13E)-Labda-7,13-dien-15-yl diphosphate; (13E)-Labda-7,13-dienyl diphosphate	30473612.691	19042190.559	30450345.129	36464857.810	28607768.655	33475384.272	14227199.816	16686668.098	15218286.158
M-Coumaric acid	24024166.775	15972253.213	30512751.383	7900393.357	7712041.004	2420097.411	10502597.675	15834044.351	10822319.419
(all-Z)-8,11,14-Heptadecatrienal	458281.142	607647.021	1401462.530	1063315.731	838746.390	1108757.022	558852.398	195933.112	231699.849
2,6,6-Trimethyl-1-cyclohexen-1-acetaldehyde	35207980.460	2803351.106	30759968.421	439105155.653	390624922.308	416758406.497	3434576.665	6970914.979	3609723.939
1,4-Diazabicyclo[2.2.2]octane	82889740.840	17313065.094	142250882.914	81924622.607	10845382.649	11048645.462	14736968.275	93802931.010	91709430.774
Terpendole C	46551257.954	18068474.486	64685467.489	1625531605.305	627538804.025	2089949826.822	280106878.333	354022504.325	348323143.638
(S)-Nerolidol 3-O-[a-L-Rhamnopyranosyl-(1->4)-a-L-rhamnopyrano	1656489.128	1068245.935	2423024.343	94346655.639	84133709.895	62669406.550	8834403.571	6608723.412	9898661.205

name	S1	S2	S3	Y1	Y2	Y3	Q1	Q2	Q3
syl-(1->2)-b-D-glucopyranoside]									
Icariin	43001923.262	10836034.854	20100033.877	73295923.908	25796729.357	67950759.222	47825691.249	49686132.966	54932565.871
2-Hydroxybenzyl alcohol	9521173.475	4542494.314	8572396.081	39314669.574	29201333.605	33876911.623	846921.765	732295.057	609856.194
LysoPC(18_3(9Z,12Z,15Z)_0_0)	46994039.225	25158987.202	53254241.913	2249025696.068	317377760.755	432513669.774	95458632.652	91520521.655	91041700.042
4',4'-Dihydroxyanigorootin	202214981.990	43523799.392	215208207.630	39432248.916	54933037.914	47098340.355	24243467.490	27739547.384	114436949.751
Ursodeoxycholate	28545184.025	17382830.814	30882804.406	226782.398	906958.718	682969.684	7586419.790	6908885.194	7642658.575
S-(4-Methylthiobutylthiohydroximoyl)-L-cysteine	412977.926	207696.031	386248.553	5553118.902	4812583.745	5468446.918	15676651.720	16075393.118	15972128.284
Costunolide	160310583.111	92966288.945	129833760.761	70121478.029	63572325.346	59663597.273	100119881.576	108505295.101	111007530.353
2,5-Dichloro-1,4-benzoquinone	81884104.882	26328565.804	174501404.331	17664903.492	12112258.337	16617145.944	16183315.515	15508941.370	5140012.693
LysoPC(18_1(11Z)_0_0)	13855552.428	151900641.150	319174313.548	176710342.437	1056690029.992	652042816.397	1773419381.225	100760778.673	446270933.282
1-Acetylcyclohexyl acetate	727850.450	101279325.597	244812.679	136075.075	13529551.240	16136172.351	19800227.647	89400564.079	198074.102
Nigakilactone E	55834319.025	48940118.532	62696888.146	54999488.063	47775063.021	46171770.228	15522319.831	7816581.994	8261670.079
4-Ethoxy-4-oxobutanoic acid	123059511.460	19530591.873	443540465.774	16567746.597	14819103.437	14045589.138	51038567.460	39608528.797	137126865.694
2-Phenylpropyl butyrate	82104234.032	42084225.727	75566583.912	20148303.605	18867923.870	18378145.660	114289045.950	128585682.794	126647280.238
Coenzyme Q9	28451590.269	15409276.217	43028669.345	12957979.461	12240522.561	13701668.925	19499058.768	23806144.377	14373370.105
Moxifloxacin	2798766.880	3666068.889	2647955.562	53605477.273	21898365.165	21837393.067	7935960.108	30307746.720	13323472.893
Ethoprophos	54090293.569	35457124.036	54757912.155	164721522.014	137035596.608	156274622.277	152744243.881	169781683.255	162096116.478
2-(2-Thienyl)furan	2136460.824	1018350.134	114536402.954	2431939.638	1770241.552	1526673.723	1034778.953	965024.710	1075201.614
Benzoic acid	434301668.518	282428958.707	432578505.702	705031080.720	691109261.955	628807062.788	35465970.086	30940820.047	28070560.220
2-(4-Allyl-2,6-dimethoxyphenoxy)-1-(3,4-dimethoxyphenyl)-1-propanol	3964262.158	1945343.392	2746492.744	4939426.029	3239137.935	2676048.597	1479696.453	1573262.574	1461980.474
2-Hydroxyestradiol-3-methyl ether	189520984.535	109896926.720	172163547.637	33596498.078	33858662.325	48458633.531	62333975.604	61022874.894	61727969.216
Kaempferol 3-O-beta-robinoside 7-O-alpha-L-rhamnopyranoside	15087623.678	8442119.316	13506257.692	39038355.872	47410741.772	33886389.393	11950981.574	21845277.340	17121526.908
gamma-Glutamylalanine	21005276.405	13413724.459	21639121.231	34857882.272	28710896.203	29543870.892	20352503.742	42964318.242	38387327.918
1-Ethyl-1H-pyrrole-2-carboxaldehyde	158826431.367	117892478.727	203875428.404	127444041.459	97304333.970	69426604.915	110754655.104	108528310.209	96995530.861
Corchorusoside D	6262066.462	2379849.579	3032894.539	106478895.114	88466374.274	82324907.433	26479040.984	28485351.533	28898933.474
20-Hydroxy-E4-neuroprostane	50654382.901	33791543.408	54162919.284	1623546.833	1369268.537	1646050.984	9156807.018	9853701.630	9032770.995
Theobromine	247005804.910	174408921.597	196180216.533	303518799.086	50392449.367	52155881.582	70543152.153	218065708.212	72105198.572
p-Xylene	57593653.805	37613609.861	54273335.892	18475433.523	5567799.054	6357274.872	3745274.357	6791626.486	3965117.607

name	S1	S2	S3	Y1	Y2	Y3	Q1	Q2	Q3
Sciadonic acid	3423926.986	6210536.655	7994324.180	900671.217	725417.431	1625500.539	11323817.131	7960083.247	2821285.305
Kurigalin	27213130.060	17308822.268	33044289.769	33093096.998	25463718.567	57969665.100	2769911.978	204638.058	322785.274
MG(0_0_20_4(5Z,8Z,11Z,14Z)_0_0)	65940849.591	41499705.117	68158368.585	12403079.887	3039092.481	13033225.175	22217765.109	25176813.875	25015109.061
Monobenzone	18650059.506	6377699.039	31310386.074	4593130.007	5971350.005	3905701.288	6889987.162	13317389.987	15428892.409
Octadeca-6,9,12-trienoic acid	54555452.867	26505728.574	107597242.637	62853479.488	32525747.791	72262863.853	24590699.577	44488399.189	76861086.295
7a-Hydroxy-5b-cholanic acid	15709031.690	9805618.147	15354993.402	132991.804	142232.213	44218.195	3922377.783	3228119.925	3196429.466
5-(2-Heptadecenyl)-1,3-benzenediol	80840078.455	41904415.188	65054079.705	48546185.519	37553218.167	39884917.740	28504464.087	29981370.450	26782005.553
Ginkgoic acid	171905778.837	102536129.198	165495545.569	7687410.567	6510181.360	6986695.076	6184803.085	30276277.279	5385441.448
Corticosterone	5654385.102	3297933.026	5176260.310	1557661.592	431010.582	1198617.619	1423503.275	1363091.621	1284384.040
Dihydroactinidiolide	98212781.270	61146482.691	89845799.617	29315101.008	25428163.553	25954415.161	75137086.759	81626938.435	89382645.882
beta-Caryophyllene	363396.735	59790.615	3607853.873	1463509.820	1244799.822	1657687.401	272757.424	313707.770	1611734.599
2,3-Dimethyl-5-(2-propenyl)pyrazine	69727550.454	37616074.800	60828685.837	18568101.238	9906200.495	8213386.086	25629471.249	28379869.469	23797464.128
2,5-Dihydro-2,4,5-trimethyloxazole	5145963.191	853110.281	1470176.626	2752502.580	3771390.586	4369940.420	6058434.440	6693547.556	5162086.260
Hexobarbital	49087480.389	25890108.008	41216478.340	27688274.160	21994489.633	19975470.055	26990268.155	19901206.607	26272128.310
Tubocurarine	410063362.487	269170380.862	221715171.984	68106075.409	157147630.078	62525208.520	64846643.689	318462970.534	68329330.792
3-Eicosyne	35330263.270	21792365.384	57046060.698	1616221.197	1424991.696	5288934.393	26718063.647	13602403.805	12159996.082
Sabadelin	528246.793	71715.090	214773.856	12795387.420	4904299.708	6341774.506	149924.913	2556311.948	4433069.098
LysoPC(18_0_0_0)	30316911.275	20333253.863	83765599.565	42657555.077	230650527.204	179472488.447	141350432.251	51995753.474	126234245.937
3'-Geranyl-2',3,4,4'-tetrahydroxychalcone	44986566.821	25908465.463	29893741.110	20255411.074	16875786.528	17970802.728	25265507.723	30386907.153	33043205.395
Proneurosporene	404895749.921	109902903.420	355541164.217	203930579.204	104347800.491	182248133.332	134960580.388	180587344.236	253274205.340
DG(11D3_9D5_0_0)	8983844.100	2832766.947	7057248.360	4976928.169	5520684.458	3932981.150	4015219.629	5337953.780	4211346.755
Caffeine	29047340803.514	211803310955.907	30459514871.185	113536367699.303	174047830046.378	158318279152.140	225526733019.237	216597641792.335	205028554472.359
Butyl (S)-3-hydroxybutyrate glucoside	112599674.744	30776544.172	104896980.053	44700584.428	30662750.285	30438350.335	51121269.369	66865737.146	59522868.736
Pimelea factor P2	58435582.739	18344043.608	46596227.839	9792869.246	3452971.187	790115.910	2940191.591	6027322.246	12197346.202
pymetrozine	16341853.587	6575606.613	16983154.085	806478.271	681582.132	358243.523	6660802.464	8348053.042	10399881.985
3-Icosa-5,8,11,14-tetraen-2-yloxypropane-1,2-diol	257065978.449	133981327.039	232450392.173	77927639.866	66549087.889	70969003.501	102906947.236	114500022.862	110129787.597
5-alpha-Pregnan-3,20-dione	5916635.049	3141903.946	5232102.855	3334094.894	3180426.099	3142840.259	6139612.535	7753810.779	5629620.044
Capillene	8654195.172	5214546.786	149303593.667	95018506.430	66985954.530	8980102.783	6377298.554	69849655.487	6083395.730

name	S1	S2	S3	Y1	Y2	Y3	Q1	Q2	Q3
Pyrohyperforin	3562874.613	348492102.352	985376320.312	673650763.147	1183109426.273	97420202.618	909278261.493	470758291.353	1215359183.424
Mevalonic acid	3890823.290	129955549.853	3864130.227	2249083.801	13020213.401	34582789.118	33838311.754	67461892.436	2262568.329
Kanzonol K	10524927.006	7539292.381	12294320.587	219351.141	78315.166	595261.788	5768385.880	6290349.122	5520333.373
Lucidine B	311287867.010	52278363.187	196624734.899	152736449.809	91853420.035	81568180.288	66592254.942	50507523.059	159557236.378
alpha-Bisabolol oxide B	4705642.609	6165548.614	3702722.544	1821788.586	848163.950	801338.984	2124614.831	3749401.484	2962854.331
3-(2,4-Dihydroxyphenyl)propanoic acid	79013824.646	48760820.366	117058807.337	43695017.303	47337514.078	41353035.752	37939663.852	38469434.332	41027848.967
Kaempferol 3-(2'-rhamnosylgalactoside) 7-rhamnoside	26012115.750	14680706.522	25749828.874	2912176.508	2167608.605	1852304.710	10270572.177	28381455.433	8390124.989
2-Naphthyl beta-D-glucopyranoside	7629101.425	3170818.826	4783465.498	38161791.671	28681029.136	38259260.135	18344423.078	19239706.439	26601131.090
S-2,5-Dimethyl-3-furanyl 3-methylbutanethioate	12227084.689	7603628.926	11844704.992	476869.946	3891078.423	2198411.824	3533719.109	3824537.225	5084202.188
2-(3-Hydroxy-4-methylphenyl)-5-methyl-4-hexen-3-one	108944261.009	65099308.737	104447900.064	86431805.080	95164939.046	76396923.192	39335556.392	42446313.744	39461737.206
5,7,3-Trihydroxy-4-methoxyflavanone 7-(2,6-dirhamnosylglucoside)	26410107.428	26031440.054	44768441.355	527334.498	417608.501	509246.416	12298087.914	23205441.109	8013530.260
Tanakine	4718109.538	2762380.972	4839124.135	2437790.450	1507390.089	14510415.020	2698153.369	2247027.005	2020051.475
2-Propenoic acid, 3-(2,5-dihydroxyphenyl)-	79974683.879	47486116.372	83661113.172	73735518.638	63952523.612	61152320.024	41008257.521	45275546.844	43131279.925
Anagyrine	9093868.976	3789969.164	8557202.563	11401489.239	9060639.026	11622656.747	29593267.830	36290890.073	33300197.389
ST 19_2;O3	9706843.871	5668322.955	8517924.116	5297028.816	737523.523	4254765.071	6210476.868	5740492.826	1281982.952
Quercetin 3-(3',6'-di-p-coumarylglucoside)	34976328.068	21994408.938	38900468.489	33962982.279	22652216.520	23473502.822	30449559.810	41638429.841	21591548.557
BQ-123	40332259.517	20185179.908	54044382.753	19823177.354	29341242.713	56890637.025	6845426.954	3836694.315	4445510.198
Barbituric acid	20427751.072	27379049.455	24434585.731	15259062.882	12479325.932	15191691.912	2701030.040	11121302.005	78286990.628
1-Naphthyl isocyanate	35566776.718	15325092.710	21941717.489	15794005.532	13578667.298	4052519.927	19357427.847	19799012.670	26398502.129
3',4,4'-Trihydroxypulvinone	61992138.956	30330294.695	113366133.658	32877750.703	15368265.316	17680447.053	9415858.763	8816035.098	23707141.927
Avocadene 2-acetate	6020628.993	1934327.026	4914375.067	47484085.543	38824984.174	48843891.094	23259330.103	27393232.461	51052290.137
Glaucarubolone 15-O-beta-D-glucopyranoside	801613.588	353324.844	758559.366	15467837.322	6886433.983	8316534.904	1871844.227	2119509.326	3954810.449
(2Z,4E,6E,8E)-3,7-Dimethyl-9-(2,6,6-trimethylcyclohex-1-en-1-yl)nona-2,4,6,8-tetraenal	10666152.718	7328631.593	10921396.325	338102.178	214666.091	1212517.422	4700330.634	5615592.312	5692538.693
5-(10-Nonadecenyl)-1,3-benzenediol	11436549.114	8763565.937	15400950.557	11656639.286	11460516.655	11585233.360	5552589.863	4806085.567	5492111.976
Quercetin 3-rutinoside 7-galactoside	12515687.098	8482916.162	14429949.541	19310865.133	17287392.142	19276760.016	6719944.803	6218992.646	7733740.099
Hydroquinidine	15217990.896	3545719.375	12543683.059	38482553.297	43729851.145	33111261.905	28496932.459	7785903.359	36994386.165
Vitamin A2	53490601.864	34052804.059	49874056.819	46530847.941	43699494.894	45394784.020	68915309.172	73588486.832	70408618.174
Exemestane	18277862.913	14439628.560	25054386.153	9286718.234	1665204.053	11114874.979	6477609.923	6213556.696	7378619.898

name	S1	S2	S3	Y1	Y2	Y3	Q1	Q2	Q3
(1'R)-Nepetalic acid	22363136.526	11331374.616	26421397.995	5370683.251	4652843.648	4699028.843	2681397.182	34047781.437	2770243.649
CDDO-Im	8430810.375	1709574.108	3529881.703	233465963.296	80435813.124	126720698.090	27928162.895	41425230.715	15632625.487
3-(4-hydroxyphenyl)lactate	8602709.464	3161864.854	4959275.839	35168126.273	27262406.634	31135271.602	15633975.173	22551517.252	16003460.418
Santonin	20080092.441	12281371.179	51572707.911	13674421.463	13140724.445	13997725.594	21036038.049	26308772.018	23908756.664
Estrone	7464280.552	3753750.125	6875146.113	3446163.185	2868456.275	2510754.264	30985659.866	34317412.784	33942325.064
4-Chloro-L-phenylalanine	7075832488.736	4622199165.223	7083661044.214	3438971553.851	3267914421.333	3012700259.968	4241125971.159	4329051334.681	4289888917.683
Prolyl-Methionine	27709952.512	17537969.603	28409808.057	33479443.397	32635618.595	29903573.267	41602521.363	48940323.204	51223805.150
(R)-Marmin	94543411.805	56519869.362	88018779.660	77107365.350	71155879.778	66983096.359	36748159.304	38496187.179	39577349.964
3,5-DICHLOROCATECHOL	42569227.423	142191725.177	86707298.622	70068694.793	23101243.533	141638157.406	115274961.974	548811217.593	79000457.109
Oxandrolone	215724597.367	145887516.554	217171461.966	8450455.628	6849499.705	7065628.891	59285003.089	65833638.444	66724412.460
Bullatin	51258766.725	13583519.166	46955640.068	28619310.114	38274102.935	37304368.787	28757836.125	71414279.969	33071090.234
TTPA	17443820.836	13783713.537	41455811.486	20789186.900	24891367.284	24418575.672	26396620.657	21056470.960	8198161.994
Reserpine	183340083.512	91047012.449	324877188.898	908690841.249	92419891.877	447640028.248	135626968.641	132642898.314	131585315.136
adonixanthin	17473718.408	10120971.409	14635569.209	13664900.015	12427960.820	10492970.609	38185713.628	38568171.665	33946666.385
Methylphenidate	114127554.079	73775853.986	119124265.564	1195626147.505	1064328316.121	1172669273.871	418225146.302	476371908.344	452322580.089
Securinine	15375010.121	8925816.592	13949047.097	34679376.096	31571706.371	32098208.799	11935640.457	11528733.979	8712976.665
Chloroxanthin	93873397.341	12457553.673	134648841.775	29809459.470	19350060.929	23708185.549	50715037.447	55029779.783	27131673.702
Bisphenol F	689470410.375	452335726.161	698091580.594	333182926.703	288003566.263	290564694.795	411648691.703	427483061.417	420232264.886
Galantamine	181282189.690	167241938.681	121380346.350	69245573.285	139027962.625	36670459.773	221708304.507	48684546.638	76408637.017
borneol	26242839.124	26487566.936	25571830.567	7806955.264	11035486.033	9402859.558	7964288.781	9029224.945	9675428.196
DG(20_3(5Z,8Z,11Z)_16_0_0_0)	176118011.546	76672214.328	125299138.764	68061836.730	62504608.815	58730924.462	68919967.605	73072136.526	57944507.593
Geranic acid	22037393.842	14870933.314	19311483.678	17428993.101	13519308.197	13106217.563	12173724.218	13137871.781	12481183.885
Sequoyitol	22928235765.937	23581273721.825	9043135454.122	16011026614.005	15043465966.221	6009834530.433	11430166491.857	11082183350.895	26346198368.639
Oxymatrine	109121519.435	82218440.084	140794647.372	54005407.637	43214889.155	48743742.865	224250730.090	145220819.014	175295234.473
Cyanidin 3-(6-malonylglucoside)	76682517.956	53307014.201	3766920.682	22242635.966	28413355.298	151407757.339	365911.767	1607971.471	2854841.442
15-Demethoxy-epsilon-rhodomycin	32746238.496	19210826.847	27143481.907	20709308.423	10768215.048	17168615.713	21456471.786	19120559.085	21260996.034
LysoPC(O-18_0)	25543281.944	13617297.757	45677570.571	40075728.696	39278348.810	57140837.901	40971447.332	30225041.846	62610789.632

name	S1	S2	S3	Y1	Y2	Y3	Q1	Q2	Q3
trans-4-Nonenal	20385284.562	6433416.726	19821916.224	4348229.888	2321512.708	2445587.939	1816847.522	2710711.431	4476240.958
Columbianetin	42008473.176	24419534.991	43133262.953	35694569.705	34569247.649	36034391.570	48636577.128	63255391.026	54217074.961
Iloprost	48231389.319	19541915.949	45001232.023	21203030.000	17269455.457	23086449.093	10112641.012	10068224.925	19879999.052
alpha-hydroxysalmeterol	15419819.048	9038831.628	15725390.298	86425885.540	46986836.963	4381197.923	44460249.510	17068748.852	16121993.346
Lactyltrimethylammonium betaine	41507328.925	26472986.810	44390723.706	10088432.371	16861146.815	15288040.030	95936449.675	101145868.535	106734788.329
Cystathionine sulfoxide	112478974.732	67407592.559	121678979.459	230816933.542	199613978.912	190896043.672	154280167.699	173882102.827	149864437.472
8,8-Dimethoxy-2,6-dimethyl-2-octanol	10586514.519	12553133.696	10435073.619	5556308.117	6107942.015	9605328.027	5632096.501	6243193.369	3822633.050
TG(i-15_0_i-15_0_i-13_0)	14846506.149	10066563.704	21582896.242	18960989.172	9764661.099	13141791.005	10247921.218	30511614.787	15530296.264
3-ureidopropionate	10324589.671	6367479.003	10224899.633	12082649.438	21307486.419	18157297.820	8805253.119	8869857.142	8299645.949
Stigmasteryl glucoside	66213838.005	24770249.048	95160914.813	71014500.855	46015329.910	76875001.892	79138953.674	87140728.971	99727971.067
4-(Methylnitrosamino)-1-(3-pyridyl)-1-butanol glucuronide	198545321.819	23415611.609	150373462.036	90769880.606	73462645.542	50229004.680	37924143.241	35257360.943	94103733.684
(±)-Methamidophos	11726836.504	28677799.424	49905386.792	12721202.613	4801102.073	14352773.005	7396588.382	11311419.596	18355901.621
LysoPC(16_0_0_0)	88906043.323	72687495.230	192889442.216	1176048139.961	631958900.477	176306587.925	230025977.230	205950841.073	279356114.481
Imidocarb	29417137.812	30135713.411	122656963.584	8369390.122	7690946.084	31726758.737	8711327.987	8772304.812	76724762.546
p-Menth-1-en-9-ol acetate	124145652.722	12110236.443	29105822.809	6711256.794	7639193.709	5958978.206	58373878.812	62823926.141	55673738.419
Hydrocortamate	32546269.356	38456596.747	166182489.188	20701224.367	15435048.906	11635505.044	7146262.217	96069473.626	20786610.145
5-(Hydroxyphenyl)-gamma-valerolactone-O-sulphate	13779080.090	11521615.589	13663510.837	8954916.193	5482207.716	7218547.562	2210229.771	2288857.632	1994093.481
Hydrastine	17517320.482	11120905.591	19332656.368	11877429.147	9066110.430	9342008.576	19425821.277	23591711.039	20101719.174
L-Olivosyl-oleandolide	20078029.683	38338749.102	21137275.969	84934289.777	34849706.020	72744289.957	28880195.744	31380569.065	59251760.849
Russian VX	142984327.734	82631174.680	111000873.935	5459827.443	1256718.434	1308712.249	23027334.037	23400457.120	25645289.923
Aspartyl-Glycine	5426880.014	2519435.748	4150216.022	2704075.507	1918833.867	2702014.800	1928577.941	1484678.484	1559614.821
DG(18_2(9Z,12Z)_22_6(4Z,7Z,10Z,13Z,16Z,19Z)_0_0)	59333912.878	42097456.478	35811116.077	11293539.854	9474268.040	9524575.535	20906803.753	25789512.911	29430088.411
D-Ornithine	15901432.004	7570132.632	14922576.200	4011929.321	3560451.304	3110496.721	8344493.714	8751637.472	7535957.835
Cyanidin 3-(6'-malonylglucoside)	88782860.181	18446820.908	55488524.680	89237426.905	4446684.841	247850841.182	85184.781	1418972.496	13734791.029
1-Palmitoylphosphatidylcholine	305437848.389	16772090.282	350816635.653	2457937108.218	740544788.761	598842252.368	118437129.337	59629660.479	298692500.645
Camptothecin	4318014.760	23833826.084	5720192.481	16575813.393	43469349.876	26051605.203	4063965.335	24672364.930	2848029.958
Diphenylene dioxide 2,3-quinone	107842448.661	68044922.365	113255604.046	2414127.563	2388567.445	2343224.279	13161456.584	7989298.785	3520651.410
Humulen-(v1)	198109	112948	208967	166849	152696	154455	128534	128809	147912

name	S1	S2	S3	Y1	Y2	Y3	Q1	Q2	Q3
	20.778	86.359	49.068	89.596	72.820	07.276	34.941	64.428	87.186
Cephaeline	8809052.633	5410155.056	8172782.976	2261972.730	1578621.531	1579990.282	3352271.227	6865745.409	7019608.353
ADP-glucose	41557743.820	10185714.694	25236112.805	18277201.354	6382405.264	5504542.104	7117557.209	6309881.530	18560357.354
Ganoderenic acid A	14491449.365	5143521.853	10225912.852	189978852.147	141696997.055	248134420.396	20492360.906	40759272.170	56100078.075
2-Hydroxy-2-methylbutyric acid	37661281.774	32882865.431	57301489.628	22474813.470	21799068.182	20888573.684	38897954.997	47857958.510	37286027.274
Laurolitsine	2533129.887	17764393.650	1502952.563	15129282.566	11429312.157	13952939.142	2023778.445	904400.671	1963827.814
2-Tetradecanone	39030205.718	13280186.520	50367758.917	13024336.115	2793121.006	6966059.300	10791123.397	5834359.223	12901182.849
Epilubimin	84251330.611	50159267.208	82705051.314	14731734.969	15429372.960	17406181.673	86745033.419	88979172.191	79772797.284
Mycothiol	8818748.162	4442190.922	8295355.792	4249696.502	3398872.982	4010492.368	1625268.830	71488.736	2495105.974
Indoxyl	11250445.998	11178088.122	9676223.082	2494503.641	4338757.468	5107225.761	1479066.867	5541038.223	4184396.411
Isoniazid	13122290.433	9202573.736	15034036.474	22454502.261	37627345.045	58061505.209	108560715.257	61319907.665	50434826.681
echinenone	121610897.823	39269303.255	66386766.852	51534823.836	27854695.744	16453814.539	24171773.172	35772483.033	58203475.254
Lycorine	106388254.252	61303502.056	96790356.390	27961166.206	25137678.182	24887953.913	13702714.064	15006769.528	13611887.074
Tryptophol	29043636.211	29447209.418	21045647.765	23329617.312	15488411.148	15516436.051	16300604.718	7834208.261	8214113.901
PC(16_0_16_0)	67456815.245	48404697.430	115146883.129	54120406.034	39945484.200	29693504.987	52394156.689	55114462.359	52030358.600
17a-Hydroxypregnenolone	2139232.231	3945620.501	6854782.429	2580145.516	2525745.375	2726711.782	2216082.018	2429415.681	2269274.425
2-((2E)-3,7-Dimethyl-2,6-octadienyl)-5,6-dimethoxy-3-methyl-1,4-benzenediol	17386340.584	40440876.144	16321092.780	56185981.204	45816527.832	47456834.189	30743928.432	74473712.670	75026171.867
Vanillin	231753032.152	70232478.986	115287260.135	87815542.900	88082331.658	85724300.001	123726668.599	125559500.556	124105387.073
(1S,4S)-Dihydrocarvone	57389647.853	21358280.458	41378868.987	7382229221.720	4969483490.844	6195045571.088	6690605.398	6625192.908	7036402.271
4-Methylaminobutyrate	44905186.058	134376803.796	232958912.109	96460107.838	97933437.409	57317461.369	485004757.215	97575340.175	103399294.532
Yatein	2685152.371	1216767.093	2792905.810	4154394.742	4521209.194	4381468.400	7163713.796	6641646.119	7451064.999
Gentisate aldehyde	87635686.383	50397598.476	81786212.140	136979152.205	130310959.698	138288837.111	78476288.953	88779452.460	86244325.119
Choline phosphate	22927575.632	13514175.612	25141695.821	28668804.555	23742911.010	15626928.260	23895649.658	29200425.341	28863734.549
Protoporphyrinogen IX	73375978.974	32436087.356	29332263.820	34097074.803	18762550.265	32183034.809	325389.818	1821856.169	1835917.147
D-Alanyl-D-alanine	56929229.917	35025783.959	58716284.277	12976893.789	11017573.835	10940814.306	59187546.601	68821604.464	70613579.405
2,6-Dinitrotoluene	10547380.323	23269373.679	8022824.328	19828859.420	14860359.456	5005102.623	3789867.798	10434896.469	66457753.213
2-Hydroxybenzaldehyde	122177577.572	70884553.101	121412027.424	42369067.663	38718779.433	20826877.214	43683536.181	43365411.487	85516835.479
4-Oxo-4-(3-pyridyl)-butanamide	8151424.053	4059326.301	175340224.704	110048385.028	123558026.858	142853337.814	14927703.377	18359321.976	16361071.274
Normaritidine	210007	121190	209643	877247	800910	813912	182099	185694	174779

name	S1	S2	S3	Y1	Y2	Y3	Q1	Q2	Q3
	336.598	517.710	577.567	50.525	92.305	86.733	35.126	64.708	39.234
L-Oleandrosyl-oleandolide	772261.565	344086.846	571326.036	36976955.463	34256958.000	37305063.477	14731873.364	15444511.326	13046683.939
Benzyl benzoate	20108808.050	11788448.437	16276666.992	8854593.464	7676833.177	6658056.813	13377373.938	13832416.024	14546696.316
Phenol	389250700.885	623262441.375	366850505.648	164723342.661	268113275.270	152465201.887	48548306.843	45882394.902	45422491.311
S-Methyl methanesulfinothioate	80324709.562	46612423.158	69879707.272	92333488.476	70364823.151	75665030.857	51925029.642	57512526.440	51701193.188
Canthaxanthin	37295452.947	54343463.756	42060365.033	10443495.303	46478048.394	27637484.252	20106750.116	22706739.410	44417620.719
(4-Aminobutyl)guanidine	24875424.471	14782379.009	25073460.172	53361731.259	46241948.220	48729155.762	11646245.777	12924235.023	12408188.691
Cyanurodiamide	2203898.938	1421845.328	1280552.024	9407270.424	8419012.024	8427195.815	17854674.870	17184013.880	17530005.491
Oleandolide	3368601.499	2688631.373	3347612.474	5814214.134	4639821.438	637567.738	1449685.939	1426221.537	1355646.634
magnesium-protoporphyrin IX 13-monomethyl ester	5582516.405	1148876.109	35448857.251	7342093.952	7907917.547	2474813.976	7748438.343	6068959.646	11644173.659
w Hydroxy testosterone	13579703.753	5216242.931	40905406.110	150030827.545	87216154.903	127875211.111	18555832.451	24819787.204	25452294.680
Dihydroceramide	25310053.569	114611727.770	186899782.239	88088999.751	39193201.950	54394838.537	33124683.238	44657151.876	58537412.596
meso-Diaminoheptanedioate	111672062.144	75727600.752	138622266.487	184128963.258	126462503.119	125346707.058	339403314.917	333918511.419	342823732.856
Diaminopimelic acid	87319047.586	51073345.809	87508607.337	84821780.482	74128661.610	83445875.602	155595543.616	178840413.881	179499166.309
S-Acetyldihydrolipoamide	30847218.531	22259689.086	33007050.113	42400017.567	31479229.907	43376432.520	34729885.472	61371556.433	61771588.988
Coenzyme Q2	142179402.041	87882756.350	142207303.449	95390190.789	80675625.344	18451523.960	71488224.737	75834273.434	77057669.737
Bursehernin	10640106.737	5740075.382	7533938.976	7622784.950	6362394.281	7078541.438	16429154.564	20496407.893	17911322.241
5a-Pregnane-3,20-dione	57951795.708	25415634.625	62734685.295	28204389.872	11129461.576	17028257.309	3811892.106	3892573.463	26993797.194
Pregnenolone	113347815.034	73809148.065	133274045.235	34346409.346	36600455.134	38462150.752	54828401.033	60039554.856	55863395.106
furaneol (keto form)	21402976.440	29295670.976	22422267.450	10167090.085	29850864.834	60890325.416	19636980.194	62705907.479	10125588.295
Guanine	712539431.799	443543154.161	717829634.084	256753532.216	217933574.571	231678547.708	599303414.674	643871405.883	649199836.682
Dehydrohistidyltryptophyldiketopiperazine	4153797.067	3548104.471	4019106.770	10743163.918	14072514.034	7996962.294	5864795.322	8698098.712	9415965.384
2,4-DINITROTOLUENE	292734627.713	170791175.888	299101966.091	11698519.700	10638697.387	14270475.870	688019865.297	723724142.915	717198313.946
Protoporphyrin IX	9161928.879	4717302.696	10449793.027	6665397.569	5519800.034	6401268.905	28280.997	573845.147	226406.655
Benzeneacetonitrile	68084285.817	39557013.384	65464715.700	50859646.615	35984979.950	42652071.789	37701028.084	38857219.519	35745404.074
Erythronolide B	10498234.199	4688826.825	6257365.180	3955750.283	3697274.879	1826198.409	3416220.194	3460399.757	2702553.259
Coumarin	10317464.049	6381511.292	9715385.979	12538717.543	9897507.858	10393186.199	7348728.774	7095362.498	7643119.982
androst-5-ene-3,17-dione	776955.289	414750.814	1585501.219	27669603.239	15909100.492	23851397.633	3256181.673	4180585.694	4559045.918
(E)-2-octenal	1728306845.78	1115734420.05	1869400548.41	69645088.126	58997224.393	58326350.844	456328092.903	526453848.632	487760501.203

name	S1	S2	S3	Y1	Y2	Y3	Q1	Q2	Q3
	6	4	2						
5,6-dihydroxy-3-methyl-5,6-dihydroquinolin-2(1H)-one	20557155.179	9017647.524	19441052.509	7419311.503	9171344.559	9891137.096	13372568.433	13252112.147	14365856.766
3-hydroxybenzyl alcohol	271939915.002	90535921.027	151230308.057	134300907.683	43685419.099	123082944.783	14316930.303	41131515.183	32443572.851
3,4-Pyridinediol	51662390.714	30180196.620	50598740.300	3192070.839	3584230.933	4407304.043	96436395.061	108009252.455	108358539.985
Dihydroisopentenyldehydrorhodopin	11361440.388	19168614.278	42479490.326	1598320.402	1154764.013	1393877.473	7386814.050	15543046.794	16828592.856
5,6-Dihydroxyindole	47438924.878	30404036.977	5081651.292	15645108.330	11236810.984	13211168.576	30348661.652	32294659.502	32407301.493
hexahomomethionine	30975070.013	15012866.570	36437551.889	24382469.318	18563354.989	22643827.066	149403.991	666867.337	845679.370
Kinetin	45381679.395	29839780.845	42448845.351	765792233.406	623266854.629	691775755.870	340068975.656	339794404.433	457736120.956
Phycocyanobilin	1094485232.284	1125335630.829	4378389813.837	1477849111.564	1093659031.293	1248301511.221	1096535634.766	919073760.160	1115690031.733
Deoxyinosine	288887755.491	191777632.307	301446847.950	26909346.548	21675360.407	27671789.850	238369578.132	239692277.463	223738462.334
Pyridoxine	149875285.278	14762421.692	127430995.798	42160304.409	46939010.799	25660682.711	37543649.293	40769189.251	62889637.151
Androstenedione	44931477.473	23912858.283	39632649.700	1174584.841	1145713.235	819716.605	14432133.973	12994036.107	13809070.893
4-Nitrophenol	347081541.291	147317416.446	75517419.746	42242411.707	44823710.722	47058436.834	424741333.129	131699346.867	355845847.847
Propanoyl phosphate	4090856.301	3893025.496	36161280.123	2440463.181	9821374.921	7743123.528	5627638.579	6015767.144	8651872.420
(14E)-2,11-dihydroxy-14-(1H-imidazol-5-ylmethylidene)-9-(2-methylbut-3-en-2-yl)-2,13,16-triazatetracyclo[7.7.0.01,13.03,8]hexadeca-3,5,7,10-tetraene-12,15-dione	8268893.227	5120739.976	5476347.135	3143342.327	2319962.242	2361612.879	8302955.218	3235976.099	8128468.264
trans-Coumaryl acetate	53170886.477	78969240.287	53486545.790	26862037.993	30426405.630	22384411.011	17986162.685	7221377.777	6779443.462
1,3,7-trimethylurate	129635112.126	94893924.867	137980408.557	73236711.329	59597445.912	65615301.176	97605481.796	110716163.727	107635311.150
(4Z,8Z)-4,8-dimethyl-12-oxotrideca-4,8-dienal	43617605.229	23914542.748	37100376.489	8431986.340	1749139.749	7298790.412	53526695.464	57047836.092	55452654.830
Rhodomycinone	28648452.535	10265974.703	21692897.909	1321553.081	1534165.955	1416037.571	35716687.564	73444405.405	47458025.250
Gamma-glutamyl-L-putrescine	11726409.344	32125136.776	12984123.426	8176150.666	5095732.285	5540009.940	5760529.533	8353184.323	7163304.422
Solavetivone	2779662.449	67865.030	1198338.313	1321295.858	35662.152	534092.168	19480.008	477773.260	763735.950
Phosphoenolpyruvic acid	2197104.149	869481.193	1262040.265	15014920.294	9460634.192	10257434.386	3276651.639	3635267.299	2049379.863
3,4-Dihydroxymandelaldehyde	138289653.121	79737314.118	142811004.510	82229109.623	74332617.821	74547972.322	101014789.583	113055917.146	106960811.995
L-Histidinol	1321277574.371	311562774.625	1687032951.098	621938262.267	202894365.424	372143299.400	239291787.867	109379993.864	461285277.595
4'-Demethylepipodophyllotoxin	66730164.167	72008154.756	151985139.460	1558732362.822	1398477511.377	535509634.464	236184211.838	222961185.918	467800074.381
2,3,6-Trihydroxypyridine	2642976.708	1013796.225	73516265.647	32745103.209	22475977.605	173933811.781	37663054.526	216305017.820	3092734.177
Linamarin	147953	438591	557839	606678	133380	660821	256148	180481	201337

name	S1	S2	S3	Y1	Y2	Y3	Q1	Q2	Q3
	85.453	4.823	5.731	2.413	35.335	3.123	62.510	52.929	14.894
2-Epi-5-epi-valiolone	5487661.915	3786953.679	4551371.240	12988021.330	5344984.092	14867513.942	4973446.715	5417186.515	6468478.606
alpha-CEHC	118353623.344	75019621.129	115416410.272	86813751.031	81645388.145	79319498.202	90546990.662	96226527.500	102355748.957
5-Chloro-3-methylcatechol	21323998.827	12781160.101	21221638.826	9480311.168	9417790.270	8718644.268	13370838.385	11582399.255	12058121.010
Phenylacetaldoxime	18795861.154	4293756.012	6457942.258	5216391.985	4923425.722	3964998.759	4954424.583	8585729.723	5904845.752
(S)-4-Hydroxymandelonitrile	23712223.155	15299400.401	24264585.613	11865364.289	7721769.408	2310055.388	19092281.486	13974956.562	13716659.733
N-Ethylammelide	12733703.627	30041271.509	56915248.838	32087558.288	34840514.346	62244151.662	15027090.328	108818295.702	48745953.396
N-Methylethanolaminium phosphate	779616578.400	476921069.696	789088522.950	234074445.133	225717329.312	211689210.964	325792543.918	326654694.123	312189166.763
2,4-DINITROANISOLE	10932778.860	11450367.886	10610193.452	56746209.159	56645891.687	51720337.572	71174914.947	19506035.232	13348371.739
indole-3-glycol	7532187.165	4246299.017	7145376.935	28204634.682	25416064.190	25270778.959	38012935.167	48494947.352	39224768.159
Citrulline	13502573.278	7764119.172	14002536.466	11303503.887	8069470.463	9719060.788	30029813.355	33357753.528	30969650.819
Methyleugenol	308444135.664	150093182.836	317363901.038	756113525.468	2386173.649	2483358.269	28018063.831	29006449.421	29102382.892
L-Proline	17539632.090	10840558.655	18412342.353	126140771.484	109118855.165	109641124.879	199224903.159	221334116.839	339807168.261
4-Methoxy-2,2-bipyrrole-5-carboxaldehyde	39228021.234	35902014.442	35562555.253	3169174.396	3141223.332	2754145.209	7643619.262	7337518.015	6978976.037
dihydro-3-hydroxy-4,4-dimethyl- 2(3H)-Furanone	29887500.321	17951241.330	32271078.785	46327161.755	39718318.120	46446100.300	32703622.417	39018092.796	35363188.155
Chrysosplenetin	12146961.719	10143814.813	15049622.092	3270350.184	1834379.345	2541941.159	10800821.002	14039492.654	11850700.964
cis-1,2-Dihydroxycyclohexa-3,5-diene-1-carboxylate	220084169.239	125482105.399	199298828.118	100409062.946	59189716.496	51947784.601	86485459.831	101616279.604	64478342.201
4-Hydroxyphenylacetaldoxime	43236306.186	23538665.371	36380716.506	1895934.650	1802797.184	1867981.351	73672283.130	81058663.999	81226648.726
Pyridoxamine	63525591.070	58817606.510	69640409.437	17894606.866	12128627.069	11954908.682	37037768.348	52081633.202	41304239.472
monodechloroaminopyrrolnitrin	29508302.037	15633371.227	24791186.833	24218444.815	21599900.143	21225945.611	14104712.305	16485819.105	14304408.373
4-carboxy-2-hydroxymuconate semialdehyde hemiacetal	3769752.161	3174050.602	15789646.564	28781206.241	18751978.389	18431034.733	5135018.349	3803588.659	3347665.520
Aromadendrin	13120260.836	8604805.161	13434340.773	59619775.422	50974999.554	52621993.366	5452549.029	4187153.710	1366053.713
1,6-di-O-Galloylglucose	46651877.192	35905174.225	47281962.261	326652989.917	107501785.760	118341993.489	847541.998	946994.027	709608.138
2,4-diacetamido-2,4,6-trideoxy-alpha-D-mannopyranose	3528716.592	1831628.231	4372408.449	88208841.941	98193875.572	95635549.639	41638045.683	46875386.836	41151186.779
2,4-diacetamido-2,4,6-trideoxy-beta-L-gulopyranose	29305756.608	18671685.942	26563157.927	96205403.609	83425582.573	93232319.789	38382191.574	41066050.515	41452870.553
Xanthosine	11547784.343	6289036.359	11946777.860	3960690.574	3470829.923	3235499.735	13490291.421	13804024.434	13485240.130
Pseudoephedrine	7537973.486	4163784.556	5806693.543	17270421.812	4867701.883	5818929.453	2270723.480	2171648.825	2027000.002
Isopentenyldehydrorhodopin	321460674.748	57046818.759	202695235.372	15512813.805	13678283.349	6823505.169	95787826.844	81236568.909	178081973.714
Serylglycine	1061383.703	566970.198	622655.469	16480197.655	7387525.197	5684430.245	16990391.722	29337572.457	24979042.116

name	S1	S2	S3	Y1	Y2	Y3	Q1	Q2	Q3
L-Histidine	6325488.540	3660092.280	5107505.220	2325808.747	3677504.811	28919096.945	9986248.884	35008148.498	11196488.237
3-Dehydrosphinganine	12249736.787	6755424.907	12325653.561	2589269.040	4650838.258	2357463.603	5730411.679	7339504.986	8329177.419
Leucodopachrome	5988166326.693	22675404.525	5942963727.324	7405287.612	6164709.583	5584285.330	128514031.750	140642268.355	132561847.460
2-(dihydroxymethyl)-5-formylfuran	26878186.819	14498677.941	24263535.300	34979962.100	37493125.875	29837739.585	110898847.562	176934031.376	86256255.661
4-Coumaryl alcohol	42833710.481	10370228.134	14735810.492	6243898.071	6711523.617	6320372.271	6302677.360	7245604.609	7073719.470
(2E,4E,6E)-4-methylocta-2,4,6-trienedial	17312305.358	10523435.349	25170436.926	9630542.564	10755595.965	9263202.340	8924438.224	10009814.286	9771508.912
Tryptamine	60758678.166	28353432.374	29728563.476	9795451.097	13184699.599	12081060.238	6453292.270	13677498.632	22077922.074
(R)-3,7-Dimethyl-1,6-octadien-3-ol	69505985.509	50771474.334	91702664.720	15251600.597	12350094.398	12529492.134	11112045.363	12180624.596	12495069.147
5-Aminoimidazole ribonucleotide	458989227.192	285564300.934	492103516.696	742955140.357	656893215.896	605208254.955	304674882.184	334413996.953	340314452.026
acetoacetate	209947922.999	129087798.043	190596682.771	1117160.270	914011.412	896415.145	1729987.606	5511557.215	5721067.743
L-3-Hydroxykynurenine	27363892.691	14921390.409	25721466.057	12123428.746	2202695.926	2907398.126	7756151.341	9279188.253	8813313.915
5-(2-Hydroxyethyl)-4-methylthiazole	45124243.283	28171681.431	44047791.894	35847359.480	33338751.165	35056329.388	12362831.271	14759897.273	14104782.789
6-Hydroxymellein	67899713.572	37450723.673	62917880.827	1757834.900	1546409.151	985137.808	4062774.095	6379932.462	4100972.785
D-Fucose	89424288.293	49349027.224	72133782.191	59337852.339	61221620.611	60359101.416	38336839.883	48241588.569	36972714.436
N-Acetylputrescine	18002721.839	9807550.143	18022775.637	283204389.953	269016916.156	265068839.807	9606471.743	11677288.892	7809299.147
4-(hydroxymethyl)-2-methylphenol	63373533.484	26482503.196	43712512.932	5396110.238	4588936.488	2112883.543	24645989.862	24266332.527	27171693.620
(2E,4E,6E)-7-hydroxy-4-methylhepta-2,4,6-trienal	143784310.141	71254910.262	202694344.913	19810388.107	17099189.151	16220412.367	30935363.436	32746742.717	26128236.822
(R)-2-Hydroxy-2H-1,4-benzoxazin-3(4H)-one	153616671.803	80220144.920	128204158.525	4748034.011	3391847.716	3595874.470	40801529.692	5682171.270	35856349.340
4-Aminophenol	11801197.579	8612879.086	15162244.658	5086881.285	2935052.944	2894340.848	4157172.204	6054616.511	5132691.838
10-Deoxymethynolide	154104056.035	87334817.331	178703108.464	72543070.082	60724883.886	64271208.760	53384940.437	52656676.984	53427126.271
(Z)-4-(1-Propenyl)phenol	37775981.360	19371755.829	35463665.683	27062424.025	22545493.337	23853959.068	28048709.396	30179591.756	33419113.129
aminophenol	163922073.070	92100529.651	242092006.520	102167017.322	147462608.662	96262560.001	147870737.459	99129658.089	105006412.527
5-Hydroxyindoleacetaldehyde	5306098.017	2495733.413	5518176.263	3875755.255	1755084.456	32223904.832	9419352.085	7665069.797	7752181.960
Estradiol	68118098.466	14848595.007	18824558.852	42785616.975	59198995.320	60033852.705	19117680.952	20980197.299	21509077.064
(Z)-[(4-hydroxyphenyl)acetaldehyde oxime]	23945734.568	13691579.316	27627318.692	16909487.146	15437382.255	14070161.395	989023.293	926589.478	980992.235
Ayanin	723361.867	427970.500	313308.612	2916079.010	1840683.889	1814932.302	1761776.019	2611923.134	1634842.776
3,5_4-Trihydroxycyclohexa-1,2-dione	17869216.299	13617211.097	13330634.182	22429455.721	27525624.050	24164603.072	14259176.682	10471984.520	13046573.128
4-hydroxy-2,2-bipyrrole-5-methanol	25897934.719	15034265.831	6755787.954	12549731.302	10325177.178	9233321.776	18349405.002	18274018.533	23928029.292

name	S1	S2	S3	Y1	Y2	Y3	Q1	Q2	Q3
3,5-Dimethoxyphenol	9007626.739	21014798.511	38201999.146	2510663.049	2289718.191	1946199.632	42430937.062	77197955.261	94370761.685
Clavaminate	18798902.546	10625143.414	15946140.929	761386.339	1138440.580	1104177.528	10891447.694	17519083.221	16076528.355
N-Methylhydantoin	3809692.383	19017050.322	3319847.536	6771156.378	2094018.011	12391840.120	6911985.895	6254830.092	6401829.667
4-(Glutamylamino) butanoate	56735622.268	35846355.830	65275858.610	825523349.442	709797244.271	764194025.535	592213350.928	649223488.822	653605076.583
3,4-Dihydroxymandelic acid	124293178.204	80677428.436	142480232.278	185291580.958	165016975.452	174532928.856	215656179.933	220870740.604	228118297.013
octanoate	48902465.675	49237626.691	49821157.041	111385422.765	25119737.863	136320099.165	29273960.255	51038255.150	225614430.663
Deoxyuridine	4117018.179	4742953.434	4297759.567	2746748.038	2687072.983	3269677.828	11525710.379	11567687.825	11846209.816
p-Cresol	17789293.336	12596982.043	20556343.036	17636408.645	18472615.225	16206584.851	24882576.227	23047356.999	24839709.817
Indole-3-carbinol	77311250.703	32896040.285	65443971.144	14931698.676	9957870.719	8779969.690	23394789.696	19805090.395	19200693.175
2,4,5-Trihydroxytoluene	18014662.107	9364780.131	17058145.137	119465639.783	75591582.867	96859597.174	25198347.290	4142463.445	27678472.004
1,6,6-Trimethyl-2,7-dioxabicyclo[3.2.2]nonan-3-one	49786798.365	29084012.370	52986776.377	8238492.801	8872404.668	6883424.595	10603604.154	12119746.626	11136668.901
Homocysteine	69893886.720	30936098.637	53703015.831	11565622.492	12003085.978	10641687.547	23271219.757	30743842.116	28087394.232
brevianamide F	5198841.522	5001375.515	26687146.205	14424428.675	10136633.816	16643033.831	23272126.371	27083925.085	15764242.469
Pterostilbene	9836073.507	5790570.104	11389856.895	873912.413	1164920.035	1045438.243	9347356.895	12653875.023	11535578.615
Anhydrorhodovibrin	44074495.076	42551914.194	56856081.420	30947258.031	36688765.186	27984658.923	47356778.887	36753774.606	40619428.916
L-Phenylalanine	27278155.260	36629192.534	22255749.525	2715531.136	2645508.187	2595412.787	14290655.732	4958471.535	15604518.576
Benzyl isothiocyanate	128444853.154	78176514.065	124957357.578	24065952.941	18764922.638	18319777.554	48343329.823	47843335.014	47545750.779
Epsilon-caprolactam	22280535.482	13246229.850	20391661.492	21410536.599	14290240.592	12167259.686	9495886.883	9383125.774	8431653.139
Albaflavenone	636837012.802	383930350.612	621835183.724	665055459.913	611500397.540	637536519.765	363739055.597	404776995.498	429579081.229
germacra-1(10),4,11(13)-trien-12-al	403325728.486	253358989.407	386512750.568	463774955.666	349967655.397	398223890.737	262116598.953	287953872.233	276846637.417
Carbamoyl phosphate	15760501.212	10272296.278	16732856.515	1724975.375	308724.305	793489.734	1508155.120	1719564.537	1578580.391
Benzaldehyde	24170790.248	17945693.990	28937400.099	7479841.605	5852330.643	5713625.848	9968970.743	7849700.414	12253734.612
5-Methyltetrahydropteroyltri-L-glutamic acid	14946110.340	8343582.722	10666106.204	31267902.339	24909217.825	14979350.205	222182.853	206243.633	190767.122
Benzyl thiocyanate	26673800.315	14607853.568	25639708.340	17381589.584	13298810.812	14712524.982	14808861.376	16898269.921	16730107.189
3-(Methylthio)-1-propanol	1821863111.600	1076535107.367	1947622007.117	155080441.843	137143470.343	145610795.190	689985946.229	768767223.316	747764136.719
3,4-Dihydroxy-9,10-secoandrosta-1,3,5(10)-triene-9,17-dione	13455926.124	6535870.403	7550949.597	26269527.366	14563363.728	22354148.900	12993225.374	19974050.058	19104505.696
S-Formylmycothiol	21457792.102	13796867.728	69068658.101	11305708.299	5698281.568	3929404.411	3689729.770	3489012.341	7076794.650
1-Chloro-2-nitrobenzene	2382029607.16	17707614740.0	2483716914.43	17485445460.1	15279933674.1	15080609265.3	19216626013.6	20710114465.8	19394440893.6

name	S1	S2	S3	Y1	Y2	Y3	Q1	Q2	Q3
	4	16	0	55	37	28	90	31	00
3,5-Dihydroxyanisole	67630055.350	51032506.297	89818464.402	837580936.990	729366912.386	709626733.502	76885745.812	77295029.659	82386123.869
Chelerythrine	6113594.646	264418.752	6115278.161	4399492.374	1514944.747	1586206.216	93948.303	132483.777	1048229.933
5-Amino-4-imidazole carboxylate	1422835.112	944062.970	28451540.978	7750037.040	3352190.876	3529244.669	2263722.213	2716197.866	2734675.165
7alpha-Hydroxycholesterol	2674700.969	50796775.117	136353172.280	48008532.182	11005532.524	12562672.848	20630642.549	27076607.272	19681083.770
Protoanemonin	64752532.136	20069829.668	15665971.993	86447265.171	80081933.514	29573555.940	36194707.182	53897521.848	13781753.382
4-(4-Hydroxyphenyl)-2-butanone	47899762.026	26797850.970	66964441.578	5984881.406	5310032.969	28115219.309	48407160.148	50630052.904	50295017.445
beta-Damascenone	27635688.581	27669857.366	42733673.779	20668618.107	9445648.312	18744414.405	63657824.569	68804001.925	46334949.999
4-Pyridoxolactone	90642085.658	46253834.135	62994347.452	30305687.139	36388060.658	30865302.608	29159887.990	34263464.500	29782518.417
Histidinal	2746418.093	6244329.740	23451149.641	4387237.596	3948082.758	1533922.351	7414760.570	2685841.738	4580255.554
beta-D-Ribopyranose	95929016.075	55772164.217	93652549.447	42272427.753	29560454.866	101759880.127	42637188.304	44919584.817	50682197.458
2,5-pyridinediol	62210850.704	38089977.850	17071061.569	15360609.939	12570029.312	21465030.770	85260246.026	85389488.240	74924547.944
Hyperforin	59576476.339	7859675.667	26066215.990	10875372.160	8660065.715	11753795.352	8572071.975	7718722.060	50948880.265
2-Nitrotoluene	81011547.986	46023112.013	79654576.902	16223381.056	17980110.110	18191161.825	41709324.515	38601060.672	51371822.485
3-chlorobenzoate	9368310.172	5285002.676	8141277.313	12333453.581	9079314.091	9472596.745	6069959.358	6755369.206	6433383.396
Diethylphosphate	101036615.040	66751750.371	102936826.773	47099022.898	46589496.018	44650982.821	64093916.605	63243293.557	59843397.620
THC 4-glucoside	32126124.825	18943746.531	35873212.670	15486262.925	11638697.576	13449994.754	291725.312	319316.466	524676.142
Piperine	30164191.032	16619823.624	23647602.078	22564826.052	19742400.689	15354238.944	12949785.918	14214483.781	12859475.337
8-methylthiooctanaldoxime	1138589.407	24338399.925	30773578.286	7194992.929	2839850.395	104959634.193	2903679.786	72051904.384	3312517.861
D-Xylonate	76055713.014	55939895.740	81276408.778	4636508.015	2266183.604	2130823.408	6786874.346	4331201.614	6613026.690
Phaseollidin hydrate	5151975.774	3986834.744	5573515.339	752835.980	757789.040	615835.842	1591408.349	2703300.715	4255006.455
Biphenyl	15561081.645	7441064.277	11788248.299	2074405.165	1126067.289	1651019.434	1410527.880	1541702.406	1643152.305
Cuminaldehyde	12869756.889	11568863.323	14517925.763	31018250.087	28643104.500	27307262.388	21524302.519	12028589.092	14531895.653
2,3,4,6-Tetrahydroxybenzophenone	67088888.708	42419481.148	57240067.188	133372435.798	119032867.388	138579269.430	118906424.767	65384595.194	129168505.342
Glutathionylspermidine	34756076.935	10330334.690	22172618.816	48305269.974	22555142.440	35670759.052	19964453.748	5976747.409	42939190.470
glandicoline A	55973557.822	36060303.335	58904495.183	150470653.112	136479812.149	130093119.176	63303960.879	58814549.039	62662244.502
L-Aspartate-semialdehyde	61412073.751	61800524.215	54629637.487	34164904.853	5179971.941	8021123.066	25355828.023	27435630.389	38258809.605
L-Cysteine	13675468.631	8242542.902	13332055.863	45991115.766	40641239.962	40611329.735	2303845.719	23432434.192	23894332.926
3,6-dichlorocatechol	32082696.720	6139468.663	37888285.859	4440726.145	15130495.145	25052939.480	4382166.010	17088116.268	3818316.361

name	S1	S2	S3	Y1	Y2	Y3	Q1	Q2	Q3
PC(16_0_18_1(9Z))	64192541.671	23292481.836	31089298.983	32130142.260	26823760.171	26559132.303	31807390.318	29984044.354	36192937.899
2-Methyl-3-amylpyrrole	131033292.277	78743856.086	121690364.400	23567174.082	18853136.303	21161004.445	34531042.701	38798910.074	36114933.873
27-Deoxy-5b-cyprinol	57457786.607	10036667.089	29773748.248	3758041.611	257816.986	340697.029	354375.780	466271.443	5052949.118
Styrene	110673768.693	63027663.987	103499464.645	6479934.502	5603779.582	21039180.136	82949538.333	89353661.146	82632560.075
3,4-Dihydroxyphenylglycol	30105485.068	18368098.096	43901748.616	9607410.616	6076801.363	12345105.763	11872694.295	11381430.848	16350119.225
Leprotin	228676400.275	58010433.000	187516309.309	56535397.923	39511265.626	47638133.303	36965860.492	27111312.667	110726093.608
indole-3-acetyl-leucine	5185068.643	82116289.685	4547288.245	121666418.917	48114158.892	125170710.587	16411229.719	15603702.664	14090148.901
Ubiquinone-1	32114929.135	17917585.878	30259406.698	4415697.516	2867603.367	3788394.232	6734561.770	7002077.295	7668865.501
Boldione	15993348.952	9009177.309	14978974.003	53847325.934	57504156.683	55395551.212	16502925.502	18869583.566	17262092.851
beta-N-Acetylglucosamine	254658022.151	160325820.766	258980009.331	203921691.662	184430648.530	173546676.954	194285518.523	216399569.329	206285105.754
trans-cinnamoyl-beta-D-glucoside	34078683.301	19735550.607	54022120.197	4354924.438	5323234.822	5441037.239	18721575.851	44694051.740	19206853.889
atrazine	10108092.710	12056446.311	10460024.356	2968772.241	3574064.837	3339029.545	9500515.065	12126269.586	7962125.770
Phylloquinol	12866867.724	2835783.246	6411750.868	6213550.415	5198930.355	3434418.447	4379715.443	2734414.581	6710074.925
Isozeaxanthin	1860573483.244	18883640.725	19181851.968	1178497.809	8658096.115	8257210.599	6609319.705	248949.908	842052.084
7a,12a-Dihydroxy-5a-cholestan-3-one	98116482.654	37441422.128	123645593.403	13210792.609	7128794.702	9712054.830	8983576.999	6098931.448	10290561.424
Carbamic acid	889014697.690	434538751.936	693829522.802	845489457.416	621222996.048	646505442.237	472871940.634	534212819.565	503926184.820
(-)-cis-Rotenolone	6209046.260	3323197.226	5919194.167	23604767.244	21351621.869	20452646.288	3372408.978	5309258.173	5172322.328
Demethylmedicarpin	1322698.817	3668132.634	4604617.114	814258.046	1864876.179	1769408.912	859688.416	983350.728	1324660.239
Sphinganine 1-phosphate	69158092.157	10403630.056	68648808.629	55366774.081	11088843.224	13808345.111	7223362.768	39999885.034	145890712.481
Pectic acid	30515939.460	293114492.854	35379008.330	14282471.332	66121032.570	153888251.419	50923917.136	259268699.306	13669155.302
Ureidosuccinic acid	46751087.952	27007053.914	43461708.535	92248006.405	71453121.432	72824206.894	54297815.365	61181024.617	56197069.467
PC(16_0_18_1(9Z)-O(12,13))	105203327.411	35593482.111	102039045.098	24707101.827	22857413.386	30975792.181	27412793.737	24254793.956	41289837.772
Isoliquiritigenin	5278837.888	2961798.195	5756222.162	3705818.590	3781137.805	3301639.691	2266230.890	2483697.503	3174845.366
2-Amino-3-carboxymuconic acid semialdehyde	92580512.820	56445813.824	86655385.878	7896803.810	7263396.957	6886269.505	51988068.699	49895071.480	110824138.503
3 alpha,7 alpha,26-Trihydroxy-5beta-cholestane	22625847.067	7091281.648	17831183.883	3263927.315	578741.838	3127401.928	3461461.721	649868.180	2470050.394
(+)-Limonene	72797230.913	44635457.281	79930859.948	70223279.108	61224749.145	65356249.115	44671271.424	45121046.834	44255082.948
Iminoaspartic acid	24639185.136	315241584.529	27924359.516	77029589.759	56420576.869	65413908.024	71159205.774	76565775.798	72347469.714
Glycerol 3-phosphate	108947205.419	8733761.917	50578073.000	24921969.333	6039311.141	33455362.281	14254223.719	11459947.055	6081984.606

name	S1	S2	S3	Y1	Y2	Y3	Q1	Q2	Q3
Glycerol	265913431.725	126109142.441	272912007.863	24900185.496	21105604.791	21665876.631	98589298.407	125620750.684	117035284.118
6-methylthiohexanaldoxime	52409864.387	30555189.826	55922885.304	18549051.036	15223389.992	19742639.161	22645207.921	24875120.689	21658320.179
Deisopropylatrazine	5838043.635	2902711.491	5939614.375	1956091.701	2674387.591	2864934.712	2601434.562	2683855.395	3980966.994
CID 440908	208164965.036	61114036.396	347244754.790	168227580.020	124928238.453	153381245.456	344473301.295	395231690.411	203734750.574
Uric acid	42157389.298	22884674.071	38163251.894	17375161.738	15377403.368	15562883.304	26480978.966	26812992.277	24613616.251
Salidroside	90352148.127	56218106.018	86863718.140	70436582.440	61894144.005	65435533.247	82446432.894	83936741.771	88285384.359
Geroquinol	16019286.747	28399897.194	12361971.209	38850415.367	4062468.797	14281774.533	17390461.504	34295993.830	9395994.425
4-Nitrocatechol	89016059.051	31532977.770	52540635.937	81093083.203	33315253.246	24322001.776	129098248.061	141930245.559	135491896.021
Valienone	48041398.841	29972294.223	46158897.704	5173165.413	17318437.902	4303779.821	4574094.254	21702996.608	22679440.266
(R)-demethyl-4-deoxygadusol	131692400.556	78741794.133	127269678.814	61602771.176	37041959.568	30853587.598	57307285.480	38550685.585	42134838.210
Tricrocin	3351866.844	2291765.769	3765369.272	7117545.897	5462344.641	3244697.176	3435045.737	3907891.442	3161091.187
1,2-Dichloroethane	111744873.860	70095583.160	117574938.310	68511535.168	60308242.324	64102203.055	73853450.179	82464394.597	82308858.210
Citric acid	12464357.263	6716364.136	12527875.000	2366855.343	1886001.829	2056237.238	29289281.384	32525884.566	30871120.287
L-Leucine	30958430.279	89239157.906	29427861.084	106997696.265	94401219.197	72294008.259	27910348.578	89769269.522	26162060.584
2-Aminoacrylic acid	647043703.944	256039002.795	900543134.535	602727337.388	1961294873.617	736893576.992	272159235.665	200815148.599	1946939712.214
2-IMINIOPROPANOATE	3743148426.456	128175504.872	4383334166.484	324234770.525	116373849.438	442757759.360	131455918.844	148728041.730	1419373636.766
Crocetindial	15514166.920	8711406.959	15592745.985	1920255.189	3029956.960	3696693.755	7304725.618	7961225.386	9301217.598
Gluconic acid	7799481.361	8001153.024	7373316.399	2289729.998	3246813.541	8024279.862	1589914.384	12343333.334	2294540.121
Dimethylbenzimidazole	10205130.204	6511474.385	9636315.447	12928648.581	11652437.440	11570667.934	20026334.934	27992198.960	20524940.470
1,3,5-Trimethoxybenzene	7171761.932	4794535.974	7400707.231	680046557.018	587948239.048	643417419.392	421316.317	439825.492	470876.633
Hypoxanthine	78065668.063	45459461.888	73210537.166	33890347.685	32466777.156	29695575.014	31468024.898	42168596.433	38581125.444
Didehydroagroclavine	4714321.346	6963296.599	5694214.937	1033005.731	3396508.620	4112025.623	13123986.910	3268748.205	12758831.272
Demanyl phosphate	71285211.253	42667866.594	72660021.994	28947664.099	23615327.998	22365977.861	63536297.606	61815328.603	64190190.182
4α-formyl-4β-methyl-5α-cholesta-8,24-dien-3β-ol	275222153.205	186107042.232	320111475.924	13869811.443	13898166.813	11690234.542	40460948.512	40036030.874	34998204.677
Glycerate	6715883.644	3500399.561	6422205.732	32781757.104	33496878.569	39510900.013	3185386.115	3457763.617	4218579.218
dihydrophloroglucinol	50820668.986	33058994.269	49377835.829	15329066.576	14534858.321	12221769.905	29934463.279	33841832.984	34247986.667
Argininosuccinic acid disodium	37290433.298	23690746.273	36287753.173	21908234.674	16254547.590	15828791.997	23049008.543	23203077.894	22193519.184
3-Oxoadipate enol-lactone	690201	400833	661715	119796	956654	989250	932950	989571	961517

name	S1	S2	S3	Y1	Y2	Y3	Q1	Q2	Q3
	783.461	570.986	761.044	4242.460	639.866	450.027	110.429	812.619	743.566
CAI-1	79151453.861	48852774.587	75427992.604	21633528.217	18830004.020	19679832.009	59093831.985	63351922.397	69087963.484
L-argininium(1+)	13050720.109	6785326.230	12222441.900	8560145.153	8722184.680	9412693.253	9966397.455	9570660.968	8276677.026
L-Homoserine	23047722.281	11330327.213	19875317.024	5576879.380	5776376.249	5028558.819	5548258.841	6090758.972	4697770.101
(20R)-Ginsenoside Rh2	53186947.149	38264874.627	28725094.829	844655.357	286392.149	407235.402	2686130.044	5178345.471	19626912.429
Tylactone	89180300.056	54101013.478	93002765.614	2917794.647	2100536.095	2240340.849	32794695.367	34821067.715	37935578.856
Dihydropinosylvin	81020509.943	739835783.972	80947575.897	671840253.099	539378695.996	554441266.738	706660364.138	724585090.305	695509606.155
Stachyose	185847286.401	64546015.705	195478702.226	138273460.791	30286769.485	24217612.637	31407188.147	28496758.013	74208072.756
isolychnose	50857883.923	20080070.052	20227900.511	3996532.441	6011306.517	7422379.193	13371108.857	9215541.569	5613133.729
Dihydromethanophenazine	28286591.945	7899339.352	22390076.261	12697360.021	10143318.936	10786770.289	5926562.292	6170221.990	14687471.245
Phaseollin	9858007.460	22319662.935	992824.325	313213.737	305329.574	969857.935	6342248.169	7184170.662	6788292.680
Sinapyl alcohol	7708821.508	9580122.400	7081018.470	107650904.607	74105985.965	79159416.316	17844329.666	19156991.658	18114149.658
1,2-dehydroreticuline	14578745.374	9164270.864	14197742.585	28259722.165	22283177.965	22403162.720	19118745.641	31194439.073	20312340.837
Arbutin	9197399.860	4120781.664	8127951.124	2283957.790	1775887.462	1645030.707	8714566.872	9294744.876	21063993.371
Aflatrem	9125450.322	6550869.811	8971745.481	829800.769	414895.814	295706.663	40206625.600	29387744.539	34402367.483
1-(4-Hydroxyphenyl)-1-decene-3,5-dione	15026410.198	9804168.663	14154273.110	83440033.718	57935341.761	50001766.382	8665528.244	20050685.250	10748942.657
Norgalantamine	39681332.216	23872495.982	41025709.581	86266994.038	75369375.025	88180085.098	57574924.753	55856099.352	57608270.254
Glucosamine 6-phosphate	828563.772	11040644.182	793022.030	3274686.521	2299588.321	2388398.548	4490683.832	3110442.067	3727843.987
4-hydroxylamino-2,6-dinitrotoluene	1919554.232	1167175.647	1976144.205	577334.390	553977.869	663953.333	787855.396	764696.492	665954.026
(R)-Canadine	35303321.929	19298273.519	26839181.640	12953978.928	5002090.417	15856102.164	12318662.768	20920444.502	14876314.098
Phaseollidin	70390999.760	39384525.766	69093349.571	203092061.261	176240082.337	169784340.785	57952078.840	64569482.140	60623406.468
Palmitaldehyde	6861508.991	4534523.955	10876737.152	20186350.927	20871960.545	19740813.850	7345041.590	8572350.834	7348400.484
1,2-Bis(4-hydroxyphenyl)-2-propanol	8101246.780	2899791.975	4559928.216	12771849.060	11482886.004	12929185.105	10001460.082	10720698.993	10519019.858
N6-cis-p-Coumaroylserotonin	14854630.176	10503173.810	15121064.299	148908692.602	141346612.199	136588937.437	11229106.051	11722294.345	11132834.200
Fucoxanthin	26550707.864	11169558.693	19967203.535	11115467.004	5870363.040	8541678.250	15453056.005	14618853.051	14646961.671
5-Amino-6-ribitylamino uracil	144792254.989	86501079.222	132017731.716	2874457.276	2821773.948	2677010.753	11285618.164	12026268.580	11980114.939
2-Methoxy-9-phenyl-1H-phenalen-1-one	87263970.329	49185549.068	57330674.352	34522536.031	61015952.763	67109250.527	44592856.991	45478758.426	66785291.890
Quinolinic acid	64767668.719	37103722.372	67862024.938	27411579.899	25622923.355	26514002.578	31444867.005	32301315.227	33216644.741
Hemigossypol	457953	310863	566935	425056	387716	420618	114327	128581	120416

name	S1	S2	S3	Y1	Y2	Y3	Q1	Q2	Q3
	46.547	88.206	70.517	955.001	439.899	440.314	832.641	682.916	874.565
Imidazolone	8507077.818	7219520.592	7043398.822	2837027.217	2125595.183	2313037.202	15616444.079	16528127.411	16063401.897
N-Cyclopropylammelide	14285435.076	8679846.311	10888284.182	2815979.902	2429414.208	2110744.886	19166294.282	17018532.212	15227931.960
2(3H)-Furanone, dihydro-5,5-dimethyl-4-(3-oxobutyl)-	10113297.365	4667943.084	6473272.083	360491.096	620965.742	223298.765	3520398.804	2609028.897	4217539.216
Deoxypumiloside	1334856.406	381276.081	5206392.134	29610567.671	15350341.850	10107015.081	14408964.578	23139108.168	9604367.828
L-tetrahomomethionine	8089905.674	3870527.402	2357181.553	4574342.419	3818096.471	4207400.535	2224656.812	2545285.906	2408891.640
Rutinose	7867775.449	6181203.094	6434764.279	452971.870	1632173.090	2647357.958	2665309.017	2728253.406	4495030.454
Reumycin	31229351.715	18992957.041	20270190.039	13423654.747	7713904.964	25157387.058	11884065.409	13550870.606	14010873.491
(S)-Styrene oxide	20262312.472	12957181.220	20970921.897	8224874.817	31071326.460	7764484.011	9705336.212	11484958.226	9824343.970
4,4-dimethyl-14alpha-formyl-5alpha-cholesta-8,24-dien-3beta-ol	93188161.531	56644368.074	98394985.986	4076950.471	3315602.051	3203188.206	12282586.775	12709349.473	14717882.313
Dihydrozeatin	49693831.017	25643461.422	47072467.653	8106031.982	8844694.538	6486954.094	4043395.586	6015853.879	11267222.201
pumiloside	40530170.981	10635452.865	53077250.859	6054123.373	23871975.782	18179286.034	21244003.949	20879881.084	9958315.459
benzyl-6-hydroxy-2-cyclohexene-on-oyl	6639633.250	3244563.036	6649344.471	14335514.555	11479472.521	12809847.164	10828361.328	11421967.912	10104437.907
Methanophenazine	321094249.853	97979279.374	270316258.522	120572283.410	66608362.426	93317655.276	41723363.205	135137583.658	56746888.710
Dihydrofolic acid	156534548.812	87172810.504	132034716.355	137259522.131	133216167.033	126668089.753	100242981.995	106995752.089	107745059.237
Harmine	120226863.396	66524990.202	113607588.441	1437378.166	1386820.886	1358765.190	54803984.445	56129176.066	52746206.988
Sinapine	157171980.507	158234595.061	186682820.499	666615.622	691711.112	902440.023	35145423.504	68947650.726	65580389.433
Neomycin	1665572.380	1559878.977	1328976.754	111800202.671	111851098.436	144031028.547	29602952.114	20314296.184	51586993.783
(R)-3,3-dimethylmalic acid	48475725.957	50093304.190	17940093.155	7194862.327	75873531.621	39970523.642	54580273.034	13721636.178	22269009.307
L-Fucono-1,5-lactone	34707988.996	161813117.904	22280216.403	7784489.554	28167532.683	9678673.803	71196123.369	18044792.358	90868340.503
2-Hydroxy-2-ethylsuccinic acid	61526854.723	248569252.389	20281306.353	26240589.399	45585334.023	31911098.805	14788112.672	14848586.071	42944903.558
L-Serine	20155940.835	32620618.523	32344298.184	11150966.470	11186587.379	14146027.040	43920996.350	48839470.553	70442848.066
UDP-2-acetamido-4-amino-2,4,6-trideoxyglucose	5812250.956	15634281.241	12941030.393	104618930.731	126282562.311	152019859.052	13722982.696	3403836.804	4518576.835
27-Hydroxycholesterol	5867845.107	2748210.419	3338413.802	12742759.078	11238480.422	6506803.149	9542512.486	10952276.266	14340756.820
5-Hydroxy-2,4-dioxopentanoate	2909014.237	6124168.863	5383878.740	2220112.215	2363362.951	3002534.302	3853568.740	3934593.388	6415801.153
2-Inosose	44478020.234	86316454.761	79786352.897	94894684.470	99003345.916	118509470.071	106358802.700	116467746.586	185461635.701
Magnoline	1626260.605	4537158.953	3557363.107	23600781.188	28866402.955	29495231.963	32637274.187	43519141.550	58341081.698
Nogalonic acid methyl ester	41816969.182	43705482.663	286791508.275	74315141.521	543730647.896	195729902.052	64511471.178	6868534.021	56716638.157
Docosahexaenoic acid	10079245.422	18586972.087	15996673.103	11107206.639	11274185.937	29508736.411	12046043.101	28924178.515	18368224.945

name	S1	S2	S3	Y1	Y2	Y3	Q1	Q2	Q3
Auraviketone	10494852.138	76398670.090	28172906.265	338939715.000	575913903.154	220763067.103	6700802.017	3541027.653	3919393.846
FT-0776012	10738287.216	16774197.497	17089280.358	7422198.415	5165426.118	7419870.475	11116851.840	10145398.623	30009302.630
3-Demethylstaurosporine	3126815.891	6579532.461	6617468.676	13345571.292	13852009.562	17015699.736	9260490.157	10286342.994	17831891.808
ST 28_2;O4	4609412.514	5010446.920	14072532.757	57710040.243	21416043.077	3058158.402	6446291.829	17672753.124	11929842.359
alpha-Tocotrienol	392565.785	5114438.760	3330750.181	205101.046	6329915.832	1474113.749	5519912.642	5306450.036	5534269.936
D-1,5-Anhydrofructose	12667474.646	181570227.730	135927831.533	38609101.685	49408291.347	120022815.645	42837091.418	14831018.653	141036048.970
9S-HpOTrE	10006369.942	103142801.781	93425580.498	87185776.815	80564406.791	69738351.520	97759309.155	90917772.563	157804654.779
Prostaglandin A1	2212108.080	14579075.751	12258259.863	3933884.946	4306366.789	5763263.612	6805977.779	1837488.930	14765637.093
Adenosine	48364724.707	98248460.654	85637145.494	97432063.091	96221728.639	132067997.628	47943712.518	49251090.187	80763051.585
LysoPA(16_0_0_0)	35441870.141	49235937.489	39107639.147	155459301.405	172729449.584	222337912.890	84476551.772	79347319.239	125823039.244
Pseudouridine	18123229.739	32820885.168	26053281.473	25885045.176	21898282.428	26422992.126	167872500.692	163968228.777	275432884.455
3-O-Caffeoylshikimic acid	44984225.303	65929076.883	59502635.668	267440974.344	1148670812.541	1480302417.597	42727275.063	45325912.302	74297130.342
Gibberellin A51	8401576.091	14934336.915	13071051.102	8084861.095	7519516.477	9276583.143	11292120.006	9727691.281	17709930.985
Trichloroacetic acid	9422956.419	17693269.023	15585666.294	33895577.715	23237407.357	31239522.783	81569017.250	10132164.202	45458135.950
Lactic acid	7477169.194	13328129.183	12017388.539	3495555.034	2864733.619	4351135.908	46846280.818	52121086.583	79920605.193
5(S)-Hydroperoxyeicosatetraenoic acid	2892047.768	6018132.654	4482540.355	5758799.827	5964981.025	9260783.861	1256007.718	97892.482	366223.249
Salicylic acid	12593410.402	23928244.575	22497843.067	37832384.693	38519013.981	59198973.555	4606172.304	4278242.134	6851098.024
Phaseic acid	4918825.959	8677347.687	8065847.466	6568068.207	6539410.794	8807862.014	3206197.544	3246967.205	5171342.798
5-Nitroanthranilate	4319370.724	8985508.966	7616212.071	9019833.354	8312541.748	11951169.014	10233311.443	10181316.126	16828047.410
Tricetin	9447485.074	15843012.772	16939685.880	12482759.645	5439774.475	6617069.085	690493308.610	57066404.794	109620298.722
Astragalin	7914415.325	4384171.628	14945474.314	53622490.096	54836115.083	65321045.775	17307409.651	15440966.398	30720182.560
5,6-DHET	2827338.175	5304018.034	4076683.876	3108868.530	4342139.800	5818105.380	3604103.362	4503780.508	5585544.382
DTXSID60975124	1442197.544	2753828.992	2668225.121	16748152.877	22696029.887	22396050.719	15622415.541	14480283.028	20310454.379
2-(beta-D-Glucosyl)-sn-glycerol 3-phosphate	14643558.990	27441148.547	22647147.407	108152070.442	106645946.741	129359519.901	53743247.337	60870312.137	91460103.207
Norajmaline	500564660.392	808371674.225	691666613.084	625871173.180	670109454.391	850652141.588	885539089.893	895151168.129	1486637092.652
6-Hydroxyparmomomycin	127694.682	519735.950	582551.779	18312321.256	19139028.266	16630038.399	9689854.330	10419662.102	17681734.478
Emodin	47639635.135	138338656.903	181446100.045	17750772.816	4466924.601	5435009.097	20175025.051	17829832.515	9470481.081
2,2,3-Trihydroxydiphenylether	136651	250922	245607	195647	219718	280495	992160	119786	201022

name	S1	S2	S3	Y1	Y2	Y3	Q1	Q2	Q3
	38.252	12.974	72.507	52.918	23.960	85.671	8.813	90.370	85.466
(S)-2-(Hydroxymethyl)glutarate	12130822.794	23010612.239	19951969.124	52482260.714	55245115.429	16334396.212	201702709.958	205231345.298	350962689.542
2,2,3-trihydroxy-3-methoxy-5,5-dicarboxybiphenyl	4586937.006	4897156.444	4961210.103	8104632.874	8748002.704	10519954.488	1602895.749	1942633.447	2748486.277
Uridine	13123190.146	25599930.577	23861656.831	26818693.310	23584920.964	35561026.014	197242372.000	201845892.987	339863553.520
Butin_(molecule)	3883462.786	5150401.400	4269102.687	9477426.938	13448177.602	14978828.909	132717.623	111804.127	198207.268
Baicalein	18297215.101	38819788.800	32437188.127	14749186.725	15133908.294	20073167.439	66903390.097	69526769.060	116159488.721
Naringenin	11365404.968	24640488.132	19357446.170	37309799.950	38265802.621	49706036.061	46496863.235	48691569.463	78164259.852
16beta-Hydroxysteroid	2142286.869	4064365.934	3704516.052	608060.522	1348339.439	309572.592	438606.025	1009746.047	762762.549
Behenic acid	62508.007	64546.956	29893992.101	74704388.111	29092967.133	36779669.670	202397654.775	118466150.698	156491185.626
2-oxostearic acid	92019347.180	175974396.679	126964939.609	1906674956.701	2034965667.795	2440077637.943	790238417.268	483969045.271	1561779610.684
Prephenate	5430949.576	10898025.572	10066739.337	3902935.407	4542921.349	5608346.784	4968226.603	5309133.269	7947897.219
Germacrene A acid	29146032.534	29456750.508	31356856.297	8409137.783	7555628.580	3424570.400	5553247.578	6469910.590	10671701.272
O-Phospho-4-hydroxy-L-threonine	9257600.328	17257573.077	16569630.152	10797481.062	12097636.379	13312073.268	48263609.923	49095596.479	81886528.244
Biliverdin	511325.620	790853.956	1120581.810	386345.623	82061.698	61752.675	1513991.642	1076594.873	4665675.298
Palmitoleic acid	78621607.105	187035168.412	157155029.943	170957286.736	193354429.626	233908988.780	97645769.534	65028404.537	183551525.987
FA 18_0;O	111552769.828	96359349.294	90784636.931	52697149.265	52148131.766	25765312.188	178285165.304	333620110.924	286991493.376
ctadecanedioate (C18-DC)	664732991.162	1325764138.272	1110583327.593	39500328.743	34697111.151	10481154.089	153312407.786	14894868.997	26489854.552
Hexadecanedioic acid	87422940.841	156738880.129	142757395.727	38228469.801	41377209.559	52576190.711	86181450.281	115695050.337	128367503.993
Desaminotyrosine	412859984.226	797841880.271	721640568.817	35381260.692	35391506.177	46171465.044	74837486.423	72800354.541	121296407.425
Traumatin	1985404.815	4165183.008	13133188.511	2869591.704	3141718.207	3560620.194	7246351.023	7809178.182	9466746.918
Heptadecanoic acid	221215754.145	32029541.208	25782076.225	23556430.009	29850199.157	26750141.946	51480499.670	27061654.727	20929128.411
Deoxymyxol 2-(2,4-di-O-methyl-fucoside)	21089926.933	2745914.047	23581403.053	18725458.715	10634965.511	2533455.637	19117482.130	17570292.381	7169272.768
Thymidine	8541592.542	17095393.885	15108440.364	6737113.615	6910058.734	9542957.055	192331703.807	191176591.387	310091649.964
10,11-epoxy-3,11-dimethyl-7-ethyl-2,6-tridecadienoic acid	18135144.981	34573528.123	29403280.265	356051181.396	404127765.815	459614254.552	172044410.201	99086281.305	307368100.439
D-Sedoheptulose 7-phosphate	24635930.498	50349761.652	40174060.794	488615062.440	449123247.010	561673121.009	152025796.375	160726228.231	253768508.394
2-Hydroxyhexadecanoic acid	505289972.437	717712987.365	652055644.207	113421780.271	180020891.805	139046035.068	281902476.215	478163234.736	761390335.849
FAL 15_0	123357228.740	218262714.661	215901178.444	45819060.180	46784846.565	58425757.392	72513539.892	73505350.476	29192373.261
beta-L-Fucose	9636537.329	18479544.873	4993547.441	55875585.488	54143966.923	75276491.616	34197388.479	37263002.516	13694817.737

name	S1	S2	S3	Y1	Y2	Y3	Q1	Q2	Q3
2,7-dihydroxy-4'-methoxyisoflavanone	13689563.488	26377945.068	24125400.484	2384388.464	1394963.430	1591545.802	1571660.437	1713350.946	2468394.148
Dodecanedioic acid	2265796.169	11016116.518	3572288.246	4943200.008	6666360.102	8336184.497	2532342.288	8873578.456	4332273.893
MCULE-4400997775	1784433.846	3360765.200	3428497.915	45821413.965	52891179.798	66050452.937	40420436.849	37511715.683	57734229.346
juvenile hormone I	52024.130	6829164.353	5380659.105	52897211.039	62436564.911	79005904.754	12534256.075	7726033.029	13032581.424
4\\%27,7-Dihydroxyflavone	2092450.687	3588879.972	3671809.364	604979.752	675750.997	571272.756	2119208.223	2420417.977	4122675.034
1-alpha-D-galactosyl-sn-glycerol 3-phosphate	1535984.379	3709543.786	4775376.242	16381524.519	14948671.368	20476122.843	3394968.731	2618571.889	6419777.269
Daphnetol	30292114.304	64000312.529	59308207.512	54236740.814	57971056.166	18920300.114	209913361.497	216594567.063	362825580.205
Urobilinogen	26766272.647	41635670.248	42949408.994	249204790.914	203784290.104	317295643.260	173553578.173	190851981.938	295585689.023
3-O-Feruloylquinic acid	7617312.807	14552727.196	12520223.316	8655236.779	9214161.979	10740693.817	4270559.693	4158567.524	7336828.089
linolenate(18_3)	80253896.004	39219344.259	32264891.385	3095223144.597	3306049291.126	1272327937.112	769033524.330	507462415.590	633418243.559
Sarcosine	165536007.641	335414122.987	292385612.849	158462717.503	156642977.425	199781916.858	373420606.124	360850962.551	573832998.226
Kaempferide	11066718.500	20698932.234	19778895.758	247967.897	248273.083	240782.763	9002870.788	10352666.573	15753157.815
Tetradecanedioic acid	1636168.848	3249370.037	3279280.349	9272384.130	8313803.256	11885746.438	2065115.544	2279236.695	4513591.951
Eugenol	34164208.770	63117314.504	55095585.182	46973887.288	53100170.725	66100898.153	96706073.976	90508323.917	159999255.246
(2'E,4'Z,8E)-Colneleic acid	105846819.317	173285329.269	156019145.147	3164262134.376	3274992853.420	4157255701.534	1557169934.267	1481939024.991	2469407379.563
[(2R,3S,4R)-3,4,5-trihydroxy-5-(hydroxymethyl)oxolan-2-yl]methyl dihydrogen phosphate	66759908.615	116574127.221	102809564.813	13879106.812	14793901.962	19317849.169	52091899.103	68395694.572	86139641.215
Norcholic acid	5259290.918	9161058.146	6567008.304	1595923.351	1454922.790	1316325.155	2695484.737	2343267.674	3240893.561
Avenoleic acid	691408.062	1320403.437	1637058.653	1487419.316	1302021.862	1285052.229	1037121.107	953320.386	1499683.761
Deoxyribose 1-phosphate	1658057.954	3965381.895	2921286.727	1114825.380	1721141.158	1904672.809	6258928.400	6032105.950	11184453.395
3-carboxy-4-methyl-5-propyl-2-furanpropanoate (CMPF)	6424019.730	2813962.859	2429054.167	866586.558	941906.198	1621095.439	4238779.400	1529419.511	7200934.190
Acacetin	8935654.083	16396832.273	20786926.535	522806.987	111404.643	135602.936	5092116.974	5595001.963	9392136.603
Myristic acid	9858727.594	3553958.237	5474692.719	6834710.118	5432848.720	5700729.609	7353862.316	4065250.160	3794892.290
1-epi-Valienol 1-phosphate	57406784.135	111992019.714	99465236.317	123821299.086	142070475.488	181757320.589	152457288.916	147220686.924	240962044.616
Fraxetin	589091.499	5018283.651	4597902.002	2907354.503	3306797.510	6502728.293	4826496.214	458242.148	8577943.432
cdiGMP	4111537.725	7453687.989	9906037.505	13708195.904	6030735.839	19456317.267	4797546.767	1432214.562	4686354.398
SCHEMBL2822263	4054179.006	7698635.089	1681172.083	12243327.175	11730328.078	17594893.080	9408445.519	8935467.455	31975801.162
1,2-Benzoquinone monoimine	76809712.299	151348869.987	130829280.048	25459034.700	13172636.638	17529155.145	112137301.313	107911246.781	175194513.745
Dehydroferreirin	823616	162628	135220	526994	517301	718381	192071	199927	358819

name	S1	S2	S3	Y1	Y2	Y3	Q1	Q2	Q3
	3.441	04.483	00.492	1.139	3.134	3.734	42.541	03.588	66.354
3,5-Dihydroxy-1,4-naphthoquinone	14807737.255	30965570.100	8574043.958	4492134.868	3132069.623	7328553.316	22809988.415	18740914.112	37311074.858
2E-Dodecenedioic acid	73477709.236	127336794.145	111315914.571	67221913.465	73988580.303	92310981.395	122835828.378	120896547.516	167934154.477
3-Hydroxyl kyneurenine	6006630.047	11155733.216	10204418.822	27880779.278	29189450.246	37273765.814	14689805.297	6410157.804	7643732.585
nuatigenin 3-beta-D-glucopyranoside	6638447.173	14198342.473	18198166.771	24318822.109	75652238.020	55511390.067	103629741.388	25961931.832	21645976.406
3-Epigibberellin A1	949203.078	1705351.198	1721288.262	788725.484	884599.421	1032776.902	1533935.977	1432259.153	2429069.082
5-Hydroxyferulic acid	12726055.108	24540243.923	20491016.289	1821961.498	1794530.019	2528842.035	6564104.762	7879803.906	10589861.561
9-Fluorenone	1563622.563	2372042.897	3432644.654	594010.178	415144.497	748483.478	5135468.529	1905420.283	6561018.682
(-)-Bisdechlorogeodin	24058927.455	43808335.919	40388650.534	11060486.024	11840245.532	13832887.354	46492158.136	54184742.785	87485903.575
Quercitrin	275755821.739	485148557.208	454859310.844	553544097.622	557924100.144	715383790.531	303865607.363	300395135.431	497337583.870
6-hydroxy-N-methylmyosmine	9972344.578	17488942.029	15936396.330	8340001.257	9057076.650	11542686.493	7582017.113	7519300.186	12616714.446
Perillic acid	55646640.387	104836677.631	65552560.373	4076521.878	6807916.903	5351993.226	20776262.174	20454527.762	47200606.204
3-Oxododecanoic acid	20338853.735	26833958.002	23451525.956	13483682.535	13896880.137	18288555.666	2777088.563	2624120.394	3984633.170
L-Ribulose 5-phosphate	8394271.100	22828038.583	20467768.125	40873311.201	40305866.384	56547884.971	50808328.469	46245357.012	66125206.417
23-Nordeoxycholic acid	107516276.424	102112372.810	73586304.155	295042749.353	304367882.450	62848682.179	135146774.027	609879394.547	35178786.088
3-Sulfinylpyruvic acid	18719094.928	44972004.451	40670509.275	7732356.886	6872497.531	9260402.896	17381961.526	15549421.791	27160639.324
10-OPDA	39117990.472	60796113.915	57222478.394	204887739.310	217500676.122	267472730.177	202260057.402	208628961.390	219180631.061
Norepinephrine	2448805.082	4159513.604	3174098.712	2684929.693	2693309.773	3447189.468	3323236.320	2523118.862	6825679.590
D-Glycero-D-mannopyranose-7-phosphate	10313676.376	15340877.287	14855881.729	1363933.488	1288126.747	1587457.125	16563899.766	16367705.253	25281329.951
Coniferyl alcohol	996210.278	2055764.457	4203140.883	650327.603	508882.634	231601.887	2918359.165	3029086.826	6327353.699
mannose	4220689.223	7092534.068	6671242.672	980359.063	1076521.201	1142145.486	8136212.752	9154665.916	13969642.797
2-Amino-2-deoxyisochorismate	1368503.039	2606722.588	2366210.468	508073.363	429790.826	592299.801	3205556.709	2794416.718	1101581.201
D-Galactonolactone	6128666.214	10488715.718	9891438.521	21096365.623	21877740.070	28766841.052	31856251.919	22778557.012	37557762.347
ST 21_4;O2	19048369.646	26896712.206	23316740.693	12130642.703	16425966.131	14466539.981	30542440.975	32379799.942	38554546.422
Sebacic acid	138320859.970	255940614.203	216254809.378	56819862.525	54708359.114	71135781.395	80944317.844	87830896.879	180200619.452
Symmetric dimethylarginine	617172.338	2875854.937	3796589.059	3678386.574	2057312.409	1629662.052	2589514.661	3490384.889	5679254.138
Fustin	14822354.678	28449680.550	24891803.115	20525371.969	21629166.011	27448853.231	15910810.031	13119512.625	21777030.030
1-Naphthaldehyde	2487154.438	5452073.806	3419362.494	7178032.801	6922907.589	10001390.765	3187488.753	2904759.265	3849558.656
Farnesyl phosphate	5064519.388	9367875.054	9779270.727	5622019.717	6251447.394	8366855.069	1858380.525	1922141.604	3203513.257

name	S1	S2	S3	Y1	Y2	Y3	Q1	Q2	Q3
Trans-4-hydroxyproline	427037498.437	888087285.370	1174867274.927	89469701.983	322746201.967	269750674.222	92102500.218	82207755.005	1277200849.464
Deoxynonulosonate	11164376.866	23589803.970	19009922.718	69890013.283	71969039.166	95947315.971	38977192.758	41472275.120	67657426.430
3alpha-Hydroxy-3,5-dihydromonacolin L	732249.399	1415096.516	1460030.767	1878828.173	1795428.660	725828.688	1163970.417	1430110.827	2176973.222
Formyl-5-hydroxykynurenamine	599580.908	888318.555	1103710.786	10556395.494	343659.238	577073.781	3807826.706	11365397.062	3861161.941
JI-20A	658317.697	89862.708	91366.741	2197746.596	2619153.770	2936816.872	1459991.576	1344058.854	2092639.725
2-Undecanone	83457705.197	153702677.996	131320989.651	9283653.512	9194145.539	12763457.246	6420717.121	6481334.337	10704559.734
5,10-Methenyl-tetrahydrofolate	252994.175	1094236.067	367537.305	2646936.642	2137487.230	3682415.208	283784.150	32274.057	1319888.616
2-Biphenylol	997242.853	852781.605	911651.895	3460054.783	3687570.774	5063724.849	119680.578	211229.299	258463.410
Aflatoxin-M1-8,9-epoxide	4057365.712	7319149.078	6208170.358	7271344.456	7553367.724	9501470.759	8983759.311	10071896.929	15555639.418
17-(4-hydroxyphenyl)heptadecanoic acid	8887399.248	24007574.147	12150654.391	10241044.273	10990506.314	10637929.265	5517767.147	6091624.256	9016682.171
Pseudouridine 5'-phosphate	23515688.754	42465627.134	38808144.725	74308788.684	78174891.095	99875204.010	10230904.739	10729543.002	17998229.377
Genistin	25236103.363	50995114.634	46264137.778	34750209.646	39554619.152	53591526.830	120140253.214	122070673.001	212576581.338
6-isopropenyl-3-methyloxepan-2-one	2830498.581	5694230.186	5090886.585	3466692.401	3531670.781	5025928.486	3694673.495	3829029.495	5852334.876
10-Hydroxycarbazepine	50052842.511	98713145.219	87014784.951	141822831.151	149617028.782	205176977.881	105924449.542	111566634.538	189240310.173
beta-Glucogallin	31255485.338	54754951.699	52398942.641	203239600.284	339846365.153	96080934.452	7679447.193	8361787.218	13362621.701
Histamine	9143908.608	9929641.422	9807197.241	9173981.511	7961699.200	6770121.979	10396391.618	10962527.008	12993175.918
BPAquinone	10183305.536	17497552.603	15633267.774	5692359.109	4894233.460	6202168.506	6166830.522	7788132.179	11908701.414
6-Phosphogluconic acid	704689.132	6398320.163	1422068.407	65896837.523	67990270.250	80749220.869	3414504.273	5184397.174	5689173.775
(1R,2R,4S)-limonene-1,2-diol	31279852.988	57138400.983	52168820.355	15513612.022	20112285.395	26344377.200	32570142.813	48733013.394	80514565.285
3,4-Dihydroxyphthalate	15679279.102	29643857.410	27102904.923	2427058.869	2553509.751	2638344.107	12747593.435	13267324.018	21256233.613
Stearic acid	193807578.576	235575358.382	215353205.078	137108628.807	196355418.865	160675993.590	241058703.063	75521696.013	283976711.445
(-)-Pinocarvone	12863187.263	25201445.118	22064861.200	8215459.198	8402502.871	10801486.217	11798832.399	11751690.227	20546867.834
Prolylhydroxyproline	20001814.881	33942233.578	35607501.341	865396.260	553262.544	544608.996	11698167.986	12825446.022	20041517.039
5-Oxoavermectin 2a aglycone	3976740.006	7806496.097	5216592.321	43628917.946	46802324.017	52707155.006	37535689.727	27277745.512	56975625.824
Azatyrosine	6434910.240	11485592.151	10117744.559	1420651.626	2559391.857	1912262.105	12459396.460	4412525.949	14841200.659
N-Acetyl-L-phenylalanine	8587483.901	19153220.140	13857415.430	12539527.630	13909072.398	17380799.114	20780467.465	17342573.018	30296377.712
AminoDHQ	51258580.832	91787586.023	81698100.206	246786544.973	260260319.016	324169467.751	177693806.683	178455672.957	289598794.382
Acetylphenylalanine	7352587.518	13023840.846	10627289.353	6399898.316	6626555.695	8150048.881	27670536.643	28450961.144	43911796.173

name	S1	S2	S3	Y1	Y2	Y3	Q1	Q2	Q3
Phenylglyoxylic acid	31908247.849	62352292.253	52833667.845	4278025.221	3533091.857	4685998.396	38227749.926	19696693.309	30491626.829
2,8-Quinolinediol	13060865.992	20081183.871	17639201.584	2478174.158	3318684.463	3183662.431	50475054.502	49417831.892	85396140.741
62641-07-0	1737318.131	3055128.371	2893109.005	4809678.408	4692567.523	6172078.597	2020520.710	1241923.432	3921207.920
5,10-dihydrophenazine	3617142.794	5419686.998	6561044.033	6361217.600	7311505.182	9632588.332	10468734.048	5811745.514	15880855.743
Azelaic acid	1140893.384	2831735.642	1945157.003	1354135.774	1315422.114	1449402.447	1603952.904	1989802.495	3407404.234
5-Oxo-1,2-campholide	4591357.611	9254897.705	7601017.035	896905.574	608303.082	837613.871	14633732.694	13797929.049	23081158.791
4-hydroxymandelic acid	61654533.049	97519566.110	76940552.342	21175379.486	13648296.174	28244943.970	62977999.700	43679792.262	62168995.522
Hamamelose	2189326.974	3727283.544	4278168.524	1616389.233	1692045.986	2070107.811	2356397.620	2491974.330	3952186.181
(5S)-6-Hydroxy-5-isopropenyl-2-methylhexanoate	14133270.747	13078566.799	13143786.968	2561309.368	3099697.506	3192722.079	1215496.399	4073210.249	2506684.317
Docosanedioic acid	20491190.127	40730702.511	39216669.739	8875283.801	11013886.738	10027244.935	28160053.340	8775966.700	52278022.335
3-(3-hydroxybutanoyloxy)butanoic acid	4948632.863	9536725.142	8923994.818	15325215.103	14985119.274	20330790.291	14172420.565	13548363.615	43922336.600
5-(Carboxymethyl)proline	3039086.942	6150819.061	5090413.475	112921410.085	115840574.102	152338972.003	85143751.089	87511361.214	144811591.295
1,2-Dihydronaphthalene-1,2-diol	5460832.146	9899590.680	6824682.183	422703.905	8125337.829	679954.660	1437446.819	1471743.475	2738164.760
L-Dopa	6539621.390	13111231.867	10904012.581	11301759.377	11465716.091	14173094.233	4819628.524	690349.061	7351176.366
2,4-dihydroxy-2-heptenedioc acid	124482013.765	242616620.981	218494310.074	74469279.715	75638577.259	100645936.770	121651322.356	128318300.583	211771077.451
FA 12_3;O2	17258107.670	34089057.991	30636743.489	43879595.093	42962441.562	57109597.565	110040362.176	110016828.825	170286038.189
3-HODE + 9-HODE	8737168.713	6073186.699	5608431.899	4349955.349	4547469.699	3724771.457	8787342.664	8508047.483	9772323.521
Aconitate [cis or trans]	129971397.233	245254755.608	216250546.267	163419743.712	166599135.717	214873365.492	116540008.250	118871332.523	196390705.132
beta-Geraniol	5300096.181	10464136.701	7551627.171	7395869.431	7649228.735	12269792.487	1733456.028	1910835.319	10315116.729
Scopolin	37438790.973	73679479.722	71525924.540	219761802.807	239482323.929	285078304.039	8082543.327	7488849.273	12782949.330
Quercetin 3-rhamnoside-7-glucoside	308288831.420	451561084.025	538527349.166	501975395.643	932626640.637	1282095550.076	313488431.091	253623586.630	556042033.701
Tiglylcarnitine	38664559.872	36642542.968	62784903.810	26711771.396	36052367.477	22296484.136	29625541.674	28929488.034	38341709.865
1-Nitronaphthalene-7,8-oxide	1053568.619	2546954.702	2214781.343	284918.781	267539.263	372409.153	1741524.169	1822407.250	2567045.888
p-Cymene	897736.425	1495440.381	1378691.884	460374.152	325929.925	1737590.674	506218.516	1027900.567	1134858.470
Phenol sulfate	7202141.302	13511279.587	11908896.947	3791903.401	203335.286	312409.671	2283634.668	2083861.585	3537174.106
Pyridoxate	42648801.760	81776896.892	71653507.128	18047218.225	17709745.086	23483153.008	67409873.430	90373808.784	105855981.540
Quercetin 3-sulfate	16835084.733	31278347.296	27685079.036	35305773.251	35994663.758	47156585.015	539755132.922	525650988.600	881671794.700
4-Hydroxycyclohexylcarboxylic acid	9737853.771	18652055.286	18094223.018	11482083.166	12247781.795	15479350.398	15727325.186	16283625.854	27795892.488

name	S1	S2	S3	Y1	Y2	Y3	Q1	Q2	Q3
2-Methoxy-4-vinylphenol	37076759.718	2191143.080	14305726.648	39839459.058	45025892.311	55864552.830	121653929.893	115272727.806	193457316.278
Hydantoin-5-propionic acid	8383447.437	2877170.823	13281772.532	44946111.510	1613587.503	63581133.684	3836241.503	4741789.552	6540018.528
FA 10_0;O	63619282.995	124227511.503	109299242.541	17420193.282	17736063.083	22364453.211	5087617.117	4952919.004	8280305.170
1,2,3-Propanetricarboxylic acid	112468964.820	222509888.987	196989875.062	339783771.392	436211917.578	476937125.628	422435038.182	417353596.866	714672873.333
Aminohydroquinone	38400635.196	74008637.896	65321698.643	45684232.354	45276227.345	63208131.844	95359029.237	90639991.841	154100990.127
taxa-4(20),11-dien-5alpha-ol	695271.290	1261281.343	914217.441	432992.014	408372.969	496416.117	980296.698	1110983.269	1266891.619
(3S,5S)-Carbapenam-3-carboxylate	51262664.337	94570001.666	83435306.622	95666067.593	105820636.879	127663496.453	166451010.952	169012438.629	273610159.032
Benzamide	1744463.911	9272888.774	8102610.274	3563406.087	3395577.190	3944358.724	9038855.155	9269521.445	17859920.470
D-Arabinose	158875042.781	319869449.973	271152004.043	78660757.253	85647564.057	105033078.288	267642122.287	263913945.497	439915150.026
FA 18_3;O	1619005.672	3215455.217	5437880.051	11723812.616	7751612.296	12379046.576	14029218.843	11199128.027	16613235.549
2-Keto-6-aminocaproate	8082453.814	26810445.866	3090307.676	1225034.636	1208469.826	1605996.193	13365602.944	10974109.435	22907180.036
Glycylvaline	3209120.855	3861191.935	4319220.133	547546988.052	584385526.560	717062493.670	2255357.829	1873865.901	2780610.361
2-Isopropylmalic acid	26264761.527	37297998.669	34890497.416	6947096.597	6977513.791	8791856.320	16960730.921	18490095.587	27118707.496
Chelirubine	29207769.797	50042528.858	48537818.832	12144671.351	13682412.894	15651527.030	29864655.697	35108986.438	56956357.959
Ethyl (±)-3-hydroxyhexanoate	761414.332	1190533.314	1159504.333	662007.054	833918.864	1100589.443	978367.482	1169208.103	1803788.445
Sanguinarine	75773596.204	70972044.383	118128330.719	68228088.552	43392544.615	53216223.192	75733609.548	57376643.117	140289058.249
Lumazine	19029248.159	35753459.104	32000959.414	1842495.577	1860960.956	2192658.844	8123358.049	7558943.373	14627038.349
Cinnamic acid	20766719.691	46440622.678	36589530.846	127809376.035	129536842.946	147998382.992	122591306.399	67000321.367	106311893.211
4-coumaroylshikimate	80078.604	1118208.130	390470.421	17160188.426	21025037.588	15501393.351	211884.523	239284.955	984125.312
Kaempferol	786599.057	1297138.775	1134549.025	3996702.084	2769866.221	4939238.447	12259060.641	12996120.881	19089785.440
Cholesterol sulfate	420775053.838	215949454.453	196942590.589	14095369.617	3221179.632	10126570.935	13500983.406	9050621.133	7263645.658
Indoleacetaldehyde	5306513.825	10280327.400	9332461.631	721211.667	786419.835	606795.905	4355771.540	4508971.426	8081647.100
Naphthalene-1,2-diol	1047380.234	1948708.549	1756477.968	1178088.959	1316781.456	2013132.357	538474.928	619375.952	674082.317
2-(2,2,3-trimethyl-4-oxocyclopentyl)acetic acid	18138091.454	37976641.872	32359533.968	3680809.482	5336400.621	5325085.924	10441087.109	10879928.384	18074373.024
3-(3-hydroxyphenyl)propionate	8827848.217	17530009.755	20276210.473	18861373.669	19906024.499	25187941.852	35338300.368	29333780.407	47416121.020
N-Norgramine	7035849.606	16545195.291	13862041.211	6886379.132	4175772.461	6149682.208	2302451.505	2355107.033	3811927.183
3-sulfolactaldehyde	74720937.498	143911226.204	126684216.776	58450030.597	60893347.150	76127314.532	117538136.668	121373428.129	189796120.258
L-Erythritol 1-phosphate	1272900.880	2638673.970	2367270.302	689380.107	1302168.358	1222802.194	2064959.515	2123942.184	3686209.847
3-Amino-5-hydroxybenzoic acid	249754	451133	403614	820863	757030	963835	241546	164037	277234

name	S1	S2	S3	Y1	Y2	Y3	Q1	Q2	Q3
	58.109	78.243	46.969	3.337	8.418	1.387	30.865	57.874	74.174
3-Hydroxyanthranilic acid	10423398.077	18640131.051	17916664.602	614051.669	690453.986	3444080.072	4226179.533	20654992.825	8113379.016
3-Sulfinoalanine	9658028.659	21863027.588	17010619.578	276397.995	388674.083	478329.016	29932698.961	2764963.317	63459685.914
2-Oxohept-3-enedioate	496920444.913	939143497.571	844656430.074	131683582.863	136549862.207	178533935.810	590610870.106	609344643.419	1017393328.865
2,6-Dihydroxybenzoic acid	22084240.600	43260079.706	38828104.534	345340545.113	397417519.092	501494465.497	123347083.815	123896063.323	197215983.774
Gibberellin A9	7039495.574	12544256.095	12653952.561	19050575.273	9744238.288	11516168.789	11968734.236	13482991.464	19499826.841
4-Acetamido-2-aminobutanoic acid	54747858.040	78877497.688	92572769.032	31408403.850	32850425.104	43534037.605	123467713.823	122559775.076	201520849.376
2-Formylglutarate	1679099.406	2806867.666	2617914.434	3056790.343	3160612.310	3889373.875	4019573.304	4514538.882	6515419.118
Propioin	10155303.909	8129502.642	6466317.291	33718294.017	31541842.459	42107382.602	2144053.672	9427306.678	15853237.932
N2-Succinyl-L-ornithine	4717389.420	8642283.201	7560145.556	54387.490	37339.770	54660.302	37249781.832	38297950.533	64355203.215
3,4-dihydro-4-hydroxyphenylpyruvic acid	40336692.814	85389078.090	70654709.215	5837370.988	6535027.166	9572394.580	33936451.140	31624521.055	53662254.488
4-Oxo-1-(3-pyridyl)-1-butanone	1423933.491	1616019.727	1431469.947	4735232.915	6413416.397	1390525.453	1150990.853	912101.399	2430738.138
FO 2546M	89779.072	218421.596	188507.400	7198311.215	6962583.227	9535668.103	8417848.756	8329543.958	13692411.780
Indole-3-carboxaldehyde	76256625.339	135165269.035	120831834.598	44342669.682	44381320.562	59752098.980	66055169.653	65699377.094	114272781.533
Maleylacetoacetic acid	18330756.202	47651169.378	21760897.012	6016479.477	7476673.239	13912644.990	25489244.499	31784687.262	44311082.585
Kynurenic acid	20281109.946	36605770.808	32168958.975	15813899.088	17121497.540	21233094.339	24049563.339	25366626.229	40540000.316
2,5-Furandicarboxylic acid	164652625.689	333369062.902	279982482.476	10999048.844	10165215.431	12782274.441	92996136.380	91418449.503	144666988.326
Succinic acid	280659180.238	555615715.688	478721903.167	139730518.935	149445102.826	193923780.017	129974983.206	126420434.182	208556510.717
3-Methylbenzaldehyde	34371383.295	64721183.169	57964140.475	1599357.654	7222467.038	2027943.688	43646589.740	42699202.177	72426956.549
Ethyl (S)-3-hydroxyhexanoate	19417933.239	37167701.551	36082004.740	3306249.318	2893543.072	3539264.478	6297162.518	6598793.558	11394352.312
FA 6_3;O3	15440291.819	26567140.924	25016677.592	6226737.100	6175527.567	8598957.008	24933794.578	26410922.577	44748129.554
Adipic acid	35446760.372	73579774.544	58395297.337	15339735.765	14806416.031	18911217.252	13319606.585	5867597.524	85437287.146
Chlortetracycline	1319622.790	2097514.024	2765686.208	89824908.151	87478680.753	4432893.783	3047593.306	5250327.723	10528771.080
2-Keto-glutaramic acid	6349960.556	11390318.047	10353625.154	13406609.699	12649896.862	17084495.393	24209260.245	25118142.641	39360006.918
Adipoin	27971288.476	50923507.448	47778767.512	22806325.558	24442384.734	29582157.418	88718241.827	193808543.203	153797965.767
(R)-3,5-bisphosphomevalonic acid	1455186.664	2906791.437	2836650.842	21494102.270	22886908.851	27651611.612	9679324.212	9166969.591	13879106.424
(1S,5R)-5-Hydroxyaverantin	25167004.911	46500297.805	43066004.712	706009224.147	745244309.485	1007069546.824	18064046.742	18070513.040	33560270.318
Ribonic acid	102201212.996	188032506.748	182792538.774	6794804.897	7527216.997	10106963.700	32616546.794	33142857.232	30695067.571

name	S1	S2	S3	Y1	Y2	Y3	Q1	Q2	Q3
Juvenile hormone III	17702734.195	35463649.876	30538075.255	49273969.552	21744237.754	20994844.362	37985336.581	34092476.206	56206522.419
FA 6_3;O2	12792110.434	22393737.178	21310179.552	6903674.253	7649013.926	9127127.842	22793486.325	22550784.818	39110972.077
Nα-Acetyl-L-lysine	196845.539	3101743.586	2507643.821	1356846.702	1192470.466	1586610.224	1785787.205	2035127.868	2977092.658
Limonene-1,2-diol	23952383.851	44352091.235	42635360.620	3652406.583	3190317.476	4740233.306	185895865.176	186342958.394	303806975.027
Allantoin	24511132.858	46074976.788	40671560.362	1355861.776	4665457.861	1286166.590	7133899.661	7133029.205	11702090.838
Isoetin	1721023.334	2727329.740	3150236.458	6409477.860	10791877.080	14078305.410	38919190.526	34443561.736	50116689.779
4-hydroxyphenylacetate	13330705.755	23681245.430	21165535.810	18495372.863	22125480.211	25918985.345	31112647.418	31048518.035	50603991.879
3-Hexen-1-ol	5272771.845	8483542.357	7956856.001	15366092.614	16851555.266	21550051.321	1228681.310	1205065.202	1926488.331
6-Imino-5-oxocyclohexa-1,3-dienecarboxylate	2921779.488	4075872.729	3809890.633	2727289.056	2798479.264	3528277.662	4753340.749	3050260.440	7226892.744
Panthenol	31096457.855	61432904.471	54819561.579	12058935.767	10789918.475	14163996.917	27738673.884	26775378.358	43101150.986
Sulfoacetic acid	153438563.564	300746149.344	271340421.403	82423685.854	85063164.847	106108403.812	325685106.682	327481572.296	541432410.735
UDP-N-acetyl-D-glucosaminuronate	3365512.491	6015254.362	4855159.302	2667692.425	793556.034	2790892.504	1690153.388	1154189.569	2432739.495
Valerate	8355898.332	16966953.228	15221665.075	94127244.987	93594506.051	119193657.356	57807931.882	64085181.198	97764269.743
4-hydroxyglutamate	16634623.555	34106918.758	28482118.005	65606712.642	70412845.654	90167934.191	8670361.450	8313313.857	14235815.002
Malonic semialdehyde	18971294.027	11277035.393	8304746.344	8844865.245	10200443.353	15610564.925	18715047.801	10836676.806	84837168.665
4-hydroxyphenylethanol	96230.154	840636.542	775883.653	58839.099	25676.903	85385.417	1340091.390	1265966.649	2008544.188
6-Hydroxyhexanoic acid	719329.381	1235448.491	7082710.440	234788.244	273067.098	217306.509	841823.526	1170164.025	1201631.635
Isobutyrylglycine	7117829.434	14193590.069	11888246.351	21835608.357	6283357.633	7809994.576	9968433.787	60555447.371	95507389.701
5-Epi-valiolone	452924850.995	869871121.199	755963104.047	5120543792.082	5439482108.191	7025314012.165	1893642674.638	2037571549.306	3209532564.299
microthecin	1065983.878	1842994.740	1628157.887	5232362.127	5437016.410	6927594.866	2010803.325	11619305.381	12532562.386
7-Aminomethyl-7-carbaguanine	13464561.767	13783763.473	32180111.775	4843424.548	19232338.633	21874403.270	20916699.297	3835944.182	44307054.481
5-Acetamidovalerate	21192178.765	42992234.998	36479874.355	17257847.462	18044448.096	23158342.596	32701898.375	33061091.554	73323589.778
Urocanic acid	7839286.374	14509782.262	13220832.215	4681315.022	4540107.661	5715298.096	9464035.254	10790958.987	24380984.849
3-Nitrophenol	105794407.019	174501551.990	67468446.908	9747113.536	9344576.550	21801393.637	419540770.316	408388675.900	654353847.160
2-Propylmalate	59815644.680	119294078.405	104055492.966	371988177.481	396810854.611	516341390.072	204409128.419	197167965.154	334944162.659
Lipoamide	99370368.321	186777670.854	167653181.831	93790178.509	100940687.128	124920038.195	765831002.838	766200400.561	1280866570.615
DL-Malic acid	283137863.456	529867381.783	502509959.873	1234876256.319	1228096791.466	1576017549.726	385124567.967	382959851.540	642005752.682

name	S1	S2	S3	Y1	Y2	Y3	Q1	Q2	Q3
Cyanidin-3,5-diglucoside	68614705.309	62152582.399	223325792.131	178620963.732	33090202.227	128344978.427	28909944.355	10389507.582	6515883.498
2',4-Dihydroxy-4',6'-dimethoxy-3'-prenylchalcone	914233.240	1757177.168	352583.402	574273.566	706224.178	980728.434	196784.137	775073.194	1312094.186
(E)-1-(glutathion-S-yl)-N-hydroxy-2-(1H-indol-3-yl)ethan-1-imine	579040.332	642626.204	603503.457	1655604.878	3085481.701	2208701.036	1763776.364	650888.371	3484670.769
Threonic acid	34369321.081	63146946.216	57626479.538	150277672.869	161144998.962	210645369.451	105035690.020	105995674.243	177417607.608
4-Acetamidobutanoate	10825178.120	22047126.495	21366036.824	49630780.820	48317922.032	43980001.930	39746611.850	14430088.148	85432883.309
Hydroxyisocaproic acid	7821540.282	14965834.246	14138911.864	11085060.668	11869442.858	14206249.298	9229608.578	8960695.846	4433199.986
1,2-Benzoquinone	22713101.731	47708638.762	41160354.435	45303792.769	43304193.045	57777455.106	112687567.460	109871490.245	176274644.719
Tyrosol	123100593.842	222331651.729	195731686.802	4545284.359	4343526.878	6147014.354	825175197.184	845985151.434	1396596429.718
Coumarinic acid	11000924.534	20341789.116	18191344.538	17008791.686	17810449.909	21868262.931	10800273.606	8447806.044	3224574.390
L-Xylonic acid	167621180.976	317535699.384	275830511.726	299847058.886	314381723.797	402438022.040	1083785131.801	1075375451.771	1758585177.214
5-Hydroxymethyluracil	9610319.474	18216557.558	703186.200	11209021.541	360877.700	381598.091	746373.749	852149.474	882578.954
(R)-Acetoin	26979701.665	54569726.714	48927831.273	228136799.465	209095344.052	277698381.670	135563573.595	136038244.554	229858719.983
Cyclooctat-9-ene-5,7-diol	16505981.427	21217361.078	18117164.570	18133795.774	49526552.261	22392642.821	10465260.304	9156590.478	12422661.354
Kaempferol_3-O-rutinoside	8698255.035	6238555.067	17922523.249	6551037.616	52682247.586	32994600.862	2871299.587	6268342.163	30599654.989
CYCLOHEXANECARBOXYLIC ACID, 4-HYDROXY-3-OXO- (9CI)	42084277.539	80401616.047	76946098.282	16846392.978	21574700.521	21942192.441	39877895.780	41969744.565	72843399.534
Mandelic acid	215295820.260	401350648.763	383838266.052	69436669.102	73003292.800	100315093.734	164835668.417	185082768.887	296688353.592
Stipitatate	14238545.773	27099850.932	25004186.469	4739567.270	4521083.103	5830225.271	1960872.525	1788549.483	3221385.917
2-Deoxystreptamine	25359454.790	120951246.157	60246478.259	6216774.326	6392731.448	34310992.393	48641794.958	10703140.586	71941747.782
4-Hydroxy-3-methoxybenzenemethanol	64215051.909	122129411.200	105939702.860	7273611.096	6653300.054	8796537.415	43880159.265	45884786.569	75200839.985
[(3S,4S,5S,6R)-3,4,5,6-tetrahydroxycyclohexen-1-yl]methyl dihydrogen phosphate	103293230.997	219624402.423	182325301.890	75052531.505	70229903.118	92108436.682	35771289.530	280691866.297	320975887.100
3-Methyleneoxindole	129976509.115	247600225.644	222004450.514	11001545.116	10567058.669	11500253.759	62287195.016	62033128.279	99678470.231
4-imidazoleacetate	9471206.783	17984033.234	16840092.873	6832531.440	4972018.844	6554109.788	63856900.934	61238770.278	107056814.592
Uracil	6787977.646	14017853.410	13215826.896	15561412.719	15805699.089	20412488.972	14748279.062	15280787.338	22331374.579
3-Methylmuconolactone	37645200.972	74924686.417	67911085.942	13104874.822	12456992.430	15923732.905	43905722.126	46190467.728	72155227.702
Pyroglutamic acid	42227870.220	79650353.263	72844962.223	677626634.358	745979164.748	944905731.179	221504060.159	223431032.915	360463209.295
Citraconic acid	30869827.374	57132755.774	51273871.256	37055997.062	38345692.896	49244486.745	23915428.577	26273362.868	41587048.607
Nicotinic acid	48206557.735	28664788.456	84372918.801	28807416.428	29811801.646	34443596.578	37204601.723	41681526.704	61817106.129
Ketopantolactone	290230	573355	456060	181754	382156	484705	540153	515815	105885

name	S1	S2	S3	Y1	Y2	Y3	Q1	Q2	Q3
	20.481	20.223	69.145	76.972	01.099	14.924	57.961	81.288	662.391
5-Acetylamino-6-amino-3-methyluracil	46386647.951	22876815.412	21888797.187	174039443.229	165519803.989	223584785.370	94321575.447	94834776.065	157148082.549
2-Hydroxyethanesulfonate	45134567.815	92740151.616	78959079.207	135166504.594	142330016.043	192800783.069	117558176.480	113640651.163	185076803.937
Fumaric acid	10339870.724	18772721.068	18208808.846	50257643.237	50061551.953	63745617.233	18630762.008	19805979.264	34290138.482
Dihydroxyacetone phosphate	5723073.247	14609556.050	11609361.883	10450797.719	15816383.694	22019001.100	55053282.531	42223538.944	59941292.184
Pyrochlorophyllide	4377657.959	10833707.511	15591155.169	6692354.588	6499149.965	9692596.024	2703001.310	580462.642	9339912.891
Mesoxalic acid	1446128.469	2638555.049	2350199.779	530432.205	475360.088	1040184.893	19196777.036	6642776.526	38380635.678
2,4-Dichlorophenol	21001537.119	119348657.022	211706347.398	13230530.661	12589079.908	37633283.314	20864070.652	49053757.537	147708520.390
2-Isopropyl-3-oxosuccinate	15303269.591	43662955.563	78028829.102	17484405.054	13727344.405	22690352.741	31460305.680	33501746.136	40049169.034
epsilon-Caprolactone	1334410.522	2465643.291	2410178.511	1267733.686	1716438.190	2258660.074	4953776.580	4864666.007	8498692.767
2-(alpha-D-Galactosyl)-sn-glycerol 3-phosphate	2387312.139	5372563.705	4620923.409	8939665.394	4209322.035	19523749.829	2080744.287	3523274.729	5854461.697
Malonate	8125859.703	18525753.681	14598604.822	52323836.874	56879949.313	75665099.348	4841274.403	4543394.542	8134061.269
2-Hydroxycinnamic acid	55776332.888	108433469.893	97569791.030	76564609.061	78356320.638	100133109.377	60302177.498	59585588.842	99003303.730
2-Dehydro-3-deoxy-D-fuconate	61675423.173	166502817.824	146643560.244	9499248.097	9454275.840	257924018.932	67922584.342	49993333.647	78208656.788
FA 6_1;O3	274292351.961	304894609.727	101971598.975	11400574.526	11661387.622	45838037.226	62585156.462	143826745.551	78388199.097
N-methylanthraniloyl-CoA	3391381.101	6060140.806	3710832.354	35217712.787	71010937.805	83458857.679	596312.978	113018.798	242286.618
5-Pyridoxolactone	21594410.812	40738328.602	35206380.772	1451161.957	1943444.569	520839.006	741374.254	744628.915	1314068.532
1,8-diazacyclotetradecane-2,9-dione	22892577.571	36449857.307	35713302.220	115511354.921	105171492.134	154989182.901	54078611.751	55174752.814	93814736.514
2-Nitrophenol	46952568.352	85560141.421	78138108.194	13477589.020	12739002.910	15850289.612	45317710.841	46328143.500	80247008.671
cis-Acetylacrylate	3686974.010	5804058.141	6445051.296	3007817.864	4153080.220	4167588.173	7410224.302	6496695.556	5495965.172
alpha-Hydroxyisobutyric acid	2181608.419	4209383.220	4264197.282	11090203.829	12325768.961	644289.359	1278288.912	1348751.652	3645373.975
Iron	35270692.146	33687032.266	7072597.637	26518400.681	31276019.164	68724501.984	79489711.179	287064120.369	169022.020
ST 21_2;O3	1144672863.069	903161156.222	772832691.638	6432823.480	22862799.070	9959878.339	1037624.462	1196607.833	2046113.590
Oxidized Cypridina luciferin	29240883.795	53568293.289	59741397.599	13261510.199	13158058.881	17703340.500	8034429.582	34834602.810	38678186.911
Oxaluric acid	124638777.520	222630966.405	202957606.759	364897778.283	371781778.825	611386423.449	83651210.088	88129874.110	143889299.337
3,4-Dihydro-2H-1-benzopyran-2-one	686977.440	1413099.091	1364652.174	15697669.813	23648310.225	18947489.901	4798427.456	5152817.609	9878825.884
Permethric acid	428350.396	474816.360	1902108.170	1150025.947	1062028.101	1465086.887	108433626.361	108637506.525	167673517.611
Tetracenomycin D1	66952654.260	129134805.212	148239842.214	951689642.588	1041185160.077	1299278810.987	61240463.949	32008874.482	27691388.757

name	S1	S2	S3	Y1	Y2	Y3	Q1	Q2	Q3
2-Hydroxy-3-methylbutyric acid	12135496.358	2782428.038	26695219.654	938074.810	1075657.491	1399465.843	11985648.777	12504183.294	19784316.817
ent-3beta-hydroxycassa-12,15-dien-2-one	7470361.501	17116055.326	14408632.742	10415819.476	9616197.219	7233285.002	17634532.237	17873765.821	30160624.331
Fucose 1-phosphate	463671.454	903048.118	3651794.067	25844183.979	27374975.343	33225335.951	12793812.811	13128489.694	24387697.384
8-Methylthio-2-octanoic acid	292786.744	850883.269	738625.775	54799808.926	56695319.753	71448468.354	822307.791	914948.816	314594.923
4-Hydroxybenzaldehyde	134339482.951	240260827.974	226924576.944	17260784.646	20013244.541	24316623.926	81341175.427	89830637.169	149388705.737
Myo-Inositol	104968530.346	197072876.149	170069148.678	82387864.480	76704498.481	98675646.541	164171000.962	139201102.689	239041022.328
1,3,5-Trihydroxybenzene	9273471.217	13830019.463	15396219.822	33384864.178	36182520.793	50053144.089	7040057.572	4922806.121	2430560.946
Prostaglandin G2	2669731.682	5263294.132	4795070.668	12458218.809	14690160.554	18305688.249	2648375.092	3613760.206	5959727.717
9JHS2AVR43	12791703.641	21844318.606	19693247.353	2086595.443	10498081.158	2434145.913	3178208.973	3786435.791	4079461.550
N(omega)-Hydroxyarginine	7588705.876	13800186.121	11813144.681	9575901.188	11625806.324	12378086.187	34882822.437	32891272.514	56220067.728
5-Methylmaleylacetate	981712.361	2451150.165	1852979.120	36901099.334	611483.047	908047.243	1253978.978	1049710.680	2097735.439
5-Hydroxymethyl-2-furancarboxaldehyde	3656772.548	1987213.414	2068278.386	5854810.489	1787603.794	10465056.464	8195023.540	8593191.731	10963178.324
Ciliatine	4213383.887	7834637.059	7737117.596	3988655.210	3734580.462	4627946.608	2786981.464	3123555.435	5581745.127
Cyanidin 3-sophoroside	1434769.501	8972730.731	2765765.711	7304448.585	13324923.194	9023960.068	3213164.518	4341571.963	3403050.884
SCHEMBL21051772	161947713.459	495166255.815	21373449.702	8461561.265	29311586.340	24602415.579	52389649.193	14978004.211	72645389.002
Inosine 5'-monophosphate (IMP)	14268249.483	27601353.047	24385804.348	3266142.818	4677718.262	3944905.831	4328614.000	4381148.283	6472060.581
Gnidicin	3739772.853	8361324.017	9867198.284	37636180.488	26472784.243	46351454.043	54965772.535	62603841.289	60116435.305
Trehalose	38836108.460	67185826.442	68457815.182	99176936.737	515230.012	1596813.963	8000941.872	7656254.722	11645344.920
Galactaric acid	107679407.812	211096147.424	185534788.733	115603332.575	128336831.139	175215637.840	359098116.825	348332270.278	566133634.173
Epicatechin 3-O-(4-methylgallate)	3579898.541	9870582.583	8481995.553	2064568.010	723526.629	1200849.389	580813.667	1444115.403	3287497.664
Stigmatellin Y	43508846.060	73378805.515	73334086.546	336350718.924	364256070.057	420778711.843	187313550.011	193980919.944	304195286.991
Dimethisterone	19840585.763	33027654.848	32260659.139	828173.476	4181979.722	5365378.716	11249253.380	1125321.777	16063807.738
Protopanaxatriol	7244348.221	7570669.681	4680516.523	10604103.386	11316170.139	14294876.611	13386730.460	8710826.833	14574789.532
Baccatin_III	6614902.850	11589130.160	8521273.890	8217633.157	7942512.034	11440215.737	1303504.853	3111147.378	3588731.876
CDP-N-methylethanolamine	124548368.810	212557722.998	201766642.141	132264019.435	144133076.432	183469637.656	50873578.314	41880775.471	77125910.135
furosemide	2102161.761	4768089.510	3998480.712	3306831.795	3333551.054	4320709.172	6214614.749	6620540.655	10673877.966
Delphinidin 3-glucoside	3574187.128	13789011.807	5157834.934	3454945.843	3632135.125	2181996.056	34531741.737	29429369.153	62469792.919
Corosolic acid	61687000.747	84262328.146	79960695.913	21347585.729	26149709.966	25173194.191	272124808.664	137544339.560	522517569.137
2,4-Diacetylphloroglucinol	261997	490841	472204	884051	967185	140721	128956	133539	229297

name	S1	S2	S3	Y1	Y2	Y3	Q1	Q2	Q3
	97.055	63.028	96.391	9.690	7.370	49.281	87.198	54.856	39.436
4-Gallocatechol	12431023.491	21381314.400	17841836.486	596164114.901	622669927.087	760437062.496	24387467.174	21770844.341	29227998.038
Myricetin	15802179.238	31834763.644	24188942.924	79314503.435	82688095.614	100785647.188	113585628.197	108803733.907	154753985.280
1,2,6-Trigalloyl-beta-D-glucopyranose	17748340.210	44241082.646	35581534.238	323390370.934	371012109.519	446026751.393	23041171.421	14545845.969	20081923.791
Cytochalasin Ppho	1692153.898	2312529.905	2721000.777	5705476.394	8891999.804	8940436.432	5876529.994	7570908.559	9453783.593
6-Methylmercaptopurine	5557644.720	9168266.875	135669729.796	5936374.861	6067283.938	8598100.446	12667186.209	22223398.904	22680979.457
PG 34_1	456702214.145	109965005.956	837536252.599	30556706.170	43439055.554	10886237.116	169069464.454	199960812.346	104270438.752
17-Hydroxymethylethisterone	50716735.856	77327006.674	70096893.135	18175957.008	26077105.310	23086614.795	59171665.950	60423442.472	82490480.106
Leukotriene A4	13008383.008	23992034.901	21963722.069	11330355.349	35562534.909	26826117.404	17081498.271	17381221.097	29076331.869
Tutin	2668761.198	4224038.922	3886482.096	2314218.604	2437499.681	5315968.540	1859506.159	2020011.944	2866792.887
9,11alpha-Epoxypregn-4-ene-3,20-dione	7738104.774	11723938.104	11625053.662	6091285.711	6218121.517	8687341.511	11036615.698	12164202.304	19696613.327
ISOPHTHALIC ACID	10117593.379	27657742.377	20750130.508	6814328.863	6873830.822	8330524.956	11564553.840	17929689.490	24355050.104
2-Keto-3-deoxy-6-phosphogluconic acid	92441539.073	170532917.159	150814748.240	14185645.951	12447496.211	14030058.501	18425067.144	20294673.868	30106366.082
Lysionotin	5616629.362	11102695.553	10682459.962	7980838.156	10262194.773	12886629.753	498416.905	350684.253	431041.131
dihomomethionine	115173425.266	210722593.058	184861775.220	487566065.744	487264897.469	625406684.495	22011025.305	17195989.632	30862909.662
Nervonic acid	4790333.494	13192548.151	11877139.206	2758842.091	13443650.833	5196240.507	70641593.363	29566714.467	78537733.373
Dronedarone	81849158.658	149820357.839	138728415.024	598112705.221	268639484.196	1036200224.515	78023557.480	263786038.180	908893426.606
Propyl gallate	2235387.703	4052159.376	3323383.824	1340907.661	1335043.238	1532923.129	1989960.550	896155.010	1658482.700
FA 18_3;O3	129470177.023	258181269.302	216725442.003	46937403.328	47374796.192	72050006.160	61116072.089	236310039.084	393130129.818
Alnustone	3309690.186	4133979.448	3664087.951	719273.341	264814.564	300855.127	886449.725	2802825.891	7084169.124
Gossypin	29799485.169	18778686.094	16322018.108	2292041.903	2444881.013	742538.466	1992505.393	2110472.303	1328194.390
Phytanate	126463.640	76957002.424	67346498.182	23268066.378	26257259.919	32618916.577	38606401.589	33367428.563	65949182.611
Isofraxidin	3288680.924	5269954.552	4094043.417	29155381.750	28444536.785	38441704.914	2106924.674	2223001.486	3297635.467
Loperamide oxide	148382655.652	76962648.318	74395278.438	5385898.695	3965730.442	1438173.686	7312507.961	7307102.963	3796075.589
Pelargonidin 3-(6'-malonylglucoside)	10427339.109	21386102.506	7535664.632	5745365.343	18855567.259	23859284.693	7508771.857	9687062.704	15916749.217
D-methionine	7867311.546	15520645.325	14975957.477	1625828.219	8490275.950	9705470.396	46070023.040	48612902.168	78797455.405
FA 16_3;O	213087232.498	400330688.960	367222660.167	195245318.160	202247193.438	264896633.440	141183487.283	141745129.323	231372347.519
(-)-Abscisic acid	20544262.385	38770964.594	36589708.769	24603146.791	24909403.219	31717756.271	18987894.243	5849887.564	37792179.520
dTDP-4-oxo-5-C-methyl-L-rhamnose	75640.6	380066.	126825	290351	546129	675441	523346	710592	198860

name	S1	S2	S3	Y1	Y2	Y3	Q1	Q2	Q3
	77	026	5.445	0.502	7.196	8.525	3.492	1.842	87.775
Homoeriodictyol chalcone	5025027.416	10517403.086	15165857.354	4407930.769	5233648.012	6079316.599	41809197.438	28465016.655	20104185.108
Ellagic acid	68279277.540	142925417.661	143347732.372	122367637.352	132731149.351	177792844.938	48818454.022	159624722.465	396296355.688
2-Hydroxy-6-oxo-6-(2-hydroxyphenoxy)-hexa-2,4-dienoate	13086225.941	26150523.044	21911766.180	50003133.776	54792164.938	70505891.973	925643668.061	1014369597.753	1558315204.206
Isosakuranetin	416129318.687	814167727.915	708998471.189	440068977.841	456100809.547	587707197.581	1201181637.746	1195375003.389	2042659421.164
(S,E)-Zearalenone	55126727.602	118874598.988	85917158.245	26808665.761	29183324.014	35166986.152	50353617.496	48460911.824	78175709.583
Cichoriin	3477978.047	6141322.994	4997794.556	8113019.193	8617846.092	11358219.136	958389.773	846439.130	3054080.724
8,8a-Deoxyoleandolide	82330319.626	59295187.830	17173029.736	10334461.991	15030200.162	3557394.929	22990943.357	19261724.074	973180.014
I07-0299	18126072.029	61716958.091	329959403.526	49087851.515	86004126.414	142264176.600	39400671.453	92866760.710	29408710.309
Afzelechin	6283815.596	12300186.362	9816841.789	11739642.525	11688179.656	15351644.468	10867593.839	10587006.237	17524583.162
3-Oxodecanoic acid	4630400.886	8417336.043	8268190.544	1189986.815	1355010.700	1939931.760	7681439.322	8288858.947	13456657.325
N-Jasmonoylisoleucine	376949103.427	822109160.174	611522518.217	262710239.847	286506859.126	342334261.716	754039290.064	813887830.411	1257913393.475
Biotin	16464949.039	29567947.310	27609792.602	88936348.537	91742393.830	118400016.773	6368422.048	5759533.704	9010522.432
Hematoporphyrin	3765593.153	4702240.481	6010092.242	27775411.686	32007185.886	37946618.656	30022576.409	30225209.093	44049097.781
3-(2-hydroxyphenyl)propionate	19350254.157	33953515.150	30313743.403	5245681.329	1596376.333	7540517.079	21901740.439	101948566.671	124717705.752
5-O-beta-D-Mycaminosyltylactone	360593.027	12885804.450	17109138.732	4022684.309	88785522.603	93449722.811	252862038.994	71725005.516	179517298.461
6-(1-hydroxy-3-oxobutyl)-7-methoxy-2H-chromen-2-one	92333418.558	181117223.313	157476013.296	37754404.124	41978707.607	43053193.058	73911469.250	73790811.817	113787984.648
12-KETE	2110083.601	3987269.512	4683612.046	1292787.817	1242163.446	1671256.113	1818082.883	3237616.594	3993654.709
Histidyltryptophyldiketopiperazine	33818492.344	68295843.875	60369503.023	25989884.365	23826215.236	33437960.829	36017983.106	38168174.075	62951199.676
(+)-taxifolin	29128468.475	58651510.313	52322264.971	9920492.905	9876643.070	68302554.517	26849148.186	27832839.304	46630301.030
Hulupinic acid	15371611.472	25880262.945	20935580.017	6312796.589	7121461.606	9104875.900	21064712.580	22639243.293	30740627.303
5,6-dihydroquinoline-2,5,6-triol	562209.870	1160465.284	891701.476	5000901.993	11785942.540	16968774.908	1523430.923	2433727.996	1167366.282
Aucubin	1198350.553	2159071.248	2155798.516	7877496.295	7120215.291	8997625.288	141311.589	130939.406	165143.779
Dehydroabietic acid	163502.106	463015.327	1952613.857	250396.021	2252878.914	1011292.561	2292048.386	378119.633	2188640.501
Gingerol	38297692.265	69721325.629	67319976.381	26105354.325	28849336.868	36063588.599	36274296.875	37117048.810	61005017.578
3,7,4-Trihydroxyflavone	9808776.804	17625340.837	16089761.436	33935584.975	33739959.403	43067896.955	2199551.935	385790.713	365903.173
11Z-Eicosenoic acid（20_1）	74380273.225	178683746.321	164105622.254	75137563.880	93487586.042	81870187.508	157491381.358	42310523.408	117405148.888
8-Hydroxypinoresinol 8-glucoside	691344.	135903	907032.	408934	450568	473605	394980	427678	416966

name	S1	S2	S3	Y1	Y2	Y3	Q1	Q2	Q3
	742	3.561	730	1.748	8.925	5.376	5.670	4.762	7.343
Gossypetin 8-glucoside	49847354.117	86209645.764	80763058.428	172699072.146	177791263.040	224741617.368	55531227.902	54081519.621	91078171.540
Petroselinic acid	6579309.347	17925636.750	14313295.893	8386393.097	5584247.701	7880678.274	11385329.772	6152026.329	15718866.519
Rhodoviolascin	2945876.335	5864778.532	5005548.304	86493345.432	80909424.859	77771806.533	12035320.791	15137078.443	31735865.024
Catechin	125648944.154	223847723.114	159922689.243	2487046048.640	3609338158.054	4595816115.018	124032519.097	195842422.014	218211415.254
Fusarindin	2547214.691	4697376.311	4298184.966	1327476.111	2326920.941	2407847.336	1053377.971	1505994.947	2157225.170
Imidazoleacetic acid riboside	5241347.815	8330400.127	8680446.238	364300.092	351262.705	422539.955	5051659.897	4830078.789	9063186.085
2,7-dihydroxy-5-methyl-1-naphthoic acid	1296389.673	2189114.272	2042627.717	2634539.421	2609593.588	3179219.191	259090044.023	253536790.085	414089520.439
Delprostenato	15107307.180	19128043.274	13453709.449	49951648.779	52239080.979	69114232.678	29749490.827	30329323.916	47240499.423
2,3-Digeranylgeranyl sn-glycerol 1-phosphate	1280194617.231	1138076887.705	1297417204.079	795304421.408	414095617.540	70310471.926	261105140.784	172115890.001	120763660.303
Epicatechin	1306490.967	596592.709	2406016.629	11593748.710	50870110.714	16261072.525	640191.695	438727.738	570742.161
11beta-OHA4	13076581.268	5003940.562	13073900.114	4486673.864	5323040.562	2754295.511	5388191.463	5398523.214	6543112.825
Antheraxanthin A	13719420.485	27563212.149	20378034.307	92220596.711	84117873.507	106565460.200	117690925.695	126624952.896	130232294.457
ST 29_2;O3	6816578.539	12839826.911	12128645.152	2973048.425	3257770.183	3222081.484	5064798.139	3999630.929	7696773.417
2'-O-Methyluridine	7720460.181	14862061.650	12856775.373	1399645.546	1466539.803	1594494.204	3690180.813	5755055.115	5506470.757
Heneicosylic acid	81478653.595	81977450.136	100891329.495	85085944.950	41565995.085	47412224.843	51487574.692	67196107.818	88289074.825
Benzyl salicylate	326430.457	1647865.953	1805579.204	228607.304	792720.267	844593.517	1502920.473	1782643.145	3017050.951
Valylaspartic acid	6266043.077	12167840.742	10379563.050	81938.007	50781.695	61133.700	41854884.054	40145118.026	66729551.715
Bufalin	4134376.687	14530738.594	4579900.164	1206221.005	836028.167	290828.960	4964758.415	5487827.625	9011153.994
beta-Bixin	44468121.112	80576816.192	72737655.489	201492.276	73265.947	212274.420	4547337.710	6904352.089	7821088.635
Guattegaumerine	1257865.525	2919423.010	4247918.640	222485969.101	81388115.193	201773257.223	32733300.847	52869298.632	84638528.023
4,5-Dihydroxypyrene	26677715.754	49556118.772	41650030.156	13882114.443	14556741.166	18449976.222	349582608.847	377508179.646	575418517.256
Eicosapentaenoic acid	4884572.100	10485545.733	8600687.782	1444137.568	1470711.679	726509.793	4078813.465	3801519.558	5253333.122
9-HOTrE	180077.386	95948.589	78520.165	59331003.642	78065577.402	30411985.696	48857349.306	44141234.168	35054322.110
5,7-Dimethoxyisoflavone	2699043.146	14005423.077	4605268.042	1661419.774	1561368.565	1677675.937	6689098.800	8550973.353	14713666.269
Dihomo-gamma-linolenic acid	5707556.161	8781048.854	10446632.577	26702140.526	28320694.614	34077689.899	4710670.589	2587973.234	9750980.149
7-Hydroxy-2,4,5-trimethoxyisoflavone	531419.153	326147.696	261932.616	38963788.144	44404148.304	57314187.640	2095349.857	959746.240	4449967.501
(S)-2-acetamido-6-oxopimelic acid	7990301.879	14708713.217	12088642.793	50995567.814	58622411.734	76661688.965	91369131.993	95032189.648	161740030.614

name	S1	S2	S3	Y1	Y2	Y3	Q1	Q2	Q3
Yucron	319456.202	644682.766	476502.517	287191.796	181356.955	229278.134	437160.200	625705.617	1085640.868
9-Hydroxy-12-oxo-15(Z)-octadecenoic acid	6990595.409	14043415.901	14363048.465	20065130.525	9119732.826	7721088.837	8066056.006	1939431.812	9474753.883
Rhein	16992485.805	76266255.649	191270264.336	55989896.266	25091203.164	33972357.840	51036616.172	52981518.483	141337315.699
4-Hydroxyphenyl 4-hydroxybenzoate	1045646.430	2423931.644	2067609.404	2661182.031	2751597.686	3280040.574	209355.952	66754.572	302550.689
2H-1-Benzopyran-2-carboxylic acid, 3,4-dihydro-6-phenyl-, ethyl ester	8240286.821	15611191.695	14485591.255	4551001.639	5244101.643	6050501.869	17594925.932	17153722.596	30470711.691
6-Hydroxyflavanone	3172636.516	4978241.338	5335508.459	2488830.155	2110884.106	3016256.617	4906515.522	4287170.067	7472086.031
Sudan_IV	6149654.892	10862436.332	7366207.105	299059987.413	291592805.517	405242615.651	90496085.292	192536258.306	162779197.512
modafinil acid	13990697.898	26665963.016	21459172.314	9132168.846	7148822.005	9000066.709	1894590.757	2395405.456	3709776.016
Isorhamnetin	4872864.724	8546819.752	8564471.703	6593739.561	6882999.733	9335501.424	60092822.198	61422096.025	101121154.545
Morin	3640928.012	7099022.860	6662185.121	909031.348	688399.970	1417221.247	248488.909	367765.136	581600.034
reynoutrin	18786036.187	34995004.115	32103767.378	35609603.289	32595673.013	44049035.959	17120054.376	16491668.963	28883606.182
10-deacetyl-2-debenzoylbaccatin III	868653.482	994010.753	823223.134	12575540.497	4168423.451	11749578.185	4135606.394	4783576.062	12303589.914
Aspalathin	836212.616	1012565.405	1569160.545	18137763.052	20640232.558	25645785.971	7894917.370	7897556.772	15802689.656
Isoleucyl-Serine	3648692.641	7413090.422	6120417.501	1127696.369	1059295.071	1541954.028	1232400.634	1461613.731	2165957.759
9-cis-Retinal	1518558.364	3035755.701	2817690.234	535997.851	719882.614	819855.891	2889007.731	2903126.751	4442919.100
4-Amino-8-(2-fluoro-6-methoxyphenyl)-N-propylcinnoline-3-carboxamide	912873.079	2291801.797	6581572.414	20649575.690	29384084.126	27714239.413	7567827.838	23822265.374	12556813.211
3-(2-Furoyl)quinoline-2-carboxaldehyde	12709727.865	19167918.907	17845822.280	8054693.843	2621690.889	21348.894	2814979.743	4518954.914	6969895.806
2,4-Dibromophenol	28640102.450	51324568.992	44542638.691	27642248.837	28120973.183	36903726.614	26107195.545	22924013.805	36337910.926
ditrans,tricis-Hexaprenyl diphosphate	33477967.951	44261338.379	45549660.643	30591886.079	54554378.162	53871350.940	2981628.261	2577410.257	2058346.374
(+)-Dehydrovomifoliol	7488489.540	13226198.414	12646700.774	329282.086	224116.987	316672.444	2402403.305	3018489.625	3753655.290
N-hydroxyl-tryptamine	1570088.843	3229503.060	4784424.526	329268.963	321146.137	423610.817	2705312.048	2470365.204	4478172.785
Leucocyanidin	2531615.041	4931581.286	4044736.963	147453498.847	151752596.262	199278925.218	10422562.445	10030160.872	17136266.973
Epigallocatechin gallate	19839231.712	32198541.940	29196968.992	408348369.139	432785421.687	562295691.479	12747146.411	4297113.037	4960887.457
1-Hydroxypyrene	15156090.015	28637744.372	24906235.732	10838050.668	11240045.030	15280115.239	6344913.952	5640081.448	10151436.713
Durohydroquinone	138923493.523	112448347.006	162690761.327	131192002.047	108970689.049	48272244.840	144713832.667	135248629.865	179006054.797
ST 27_2;O3	279923.803	89719.196	59243.921	8228844.310	8628020.590	12312263.413	2610766.490	2664946.480	4968526.282
Cholesterol	10252596.262	20504854.321	19303634.563	52349981.579	55903561.143	70885592.839	71167416.198	74901479.932	118922216.424
D-Ribose 5-phosphate	5195765.997	9676536.524	8477782.110	746217.456	608657.177	1017676.631	874753.006	889378.847	1857288.180
Fenoprofen	634244.	195178	102563	180341	892132.	111766	269917	301883	422839

name	S1	S2	S3	Y1	Y2	Y3	Q1	Q2	Q3
	961	3.111	2.522	7.951	501	2.788	6.029	1.167	6.401
6-beta-D-Glucopyranosyl-8-beta-D-ribopyranosylapigenin	83345528.212	115629719.056	164982478.015	105249973.607	129662083.552	152550587.078	90679336.304	106721455.381	180834121.369
Betanidin	11619699.961	9675923.927	13709940.174	12096272.581	9912105.773	3220093.673	10844384.943	12883329.650	15963491.376
Pentadecanoic acid	6568337.999	12944014.757	11044037.169	5206928.115	5328785.731	6985513.650	3056027.403	3424439.396	5578642.939
ST 18_3;O3	3698747.577	7424345.585	388356.110	2642263.783	2631256.722	3116911.212	1890266.148	2173140.220	3137078.432
Nuatigenin	2933533.216	7123469.905	5710171.230	6902190.288	6081878.822	8378115.438	4654484.118	2541981.416	8200020.590
Bergenin	1850738.476	3796108.243	3537498.309	1635747.499	1351430.824	1395822.342	1190255.741	1334578.203	1891281.614
Prostaglandin F1a	1466763.836	1801121.272	3022350.166	1359711.289	1462601.973	840927.512	2748820.550	1427464.913	1334868.319
Demethoxycurcumin	4726607.632	459090.895	18247014.685	10696680.133	5832029.946	18956554.253	27176892.127	10824635.969	23904864.449
Glutarylcarnitine (C5-DC)	91597.593	165094.795	1178269.827	613496.955	766354.579	946593.565	2043480.253	1778421.401	3658614.992
butane-1,4-disulfonic acid	18829371.592	35162402.423	34097532.832	8543533.847	7774531.989	7639637.377	30270098.862	32208640.780	48906383.454
3-Hydroxy-N-(2-oxotetrahydrofuran-3-yl)octanamide	3616071.635	5593380.864	6452755.262	1297445.936	1380727.233	1807727.694	21382962.978	22292285.210	33705850.868
Irisxanthone	3198434.334	31968526.512	27664183.625	82613983.217	14553222.733	129627215.565	9790897.718	6675291.882	6005602.132
Vanillic acid 4-O-glucuronide	224062301.242	422699151.793	373634478.549	2587303716.360	2786922305.697	3828107645.662	170506521.906	155714853.574	292421024.344
Sulfuric acid 4-methoxyphenyl ester	148067.872	294327.755	245025.565	767128.013	715996.072	1262805.191	51253485.239	15344030.112	47313832.686
Precocene II	3405438.652	6268086.251	5538534.843	1513045.240	1641615.857	1994505.789	3740769.184	4348327.234	6432979.948
procyanidin B2	19212976.015	63071046.695	61165768.868	416224232.917	432713951.764	578905265.447	20492958.155	29418124.608	47716491.739
D-Erythrose 4-phosphate	37847961.153	71682495.875	62643961.931	2431902.034	2195786.724	3406075.701	18978279.585	4655906.134	30124701.082
(-)-beta-Phellandrene	770562.955	1407975.084	4346457.050	659818.890	695514.052	735822.481	2002327.552	2369335.128	4026198.692
Quinic acid	46597572.075	84752996.058	83596767.518	825249843.233	877217864.892	1117956862.132	347399827.394	356753371.776	589016757.184
9,12,13-TriHOME	38356605.477	71486268.813	68724840.304	33487050.132	36010767.662	45692353.873	23950113.614	26392594.979	40856058.917
Mucronine B	14145697.970	24108250.784	28835040.023	1831813.041	1418064.433	2298642.155	19188800.405	19095671.644	33163224.362
Succinic acid, sulfo-, 1,4-dioctyl ester	5361448.071	9093987.458	7028745.960	3414712.678	3421142.834	3578783.374	5469273.621	6384650.113	9716660.288
Xanthurenic acid	13749322.350	25250077.509	24998387.219	779180.771	891427.059	149652.898	12029718.995	14249709.724	23239706.688
6-amino-5-oxocyclohex-2-ene-1-carboxylic acid	5210334.972	13775662.115	12506231.788	1352910.630	911966.414	1421683.062	5716310.403	3701271.377	10441523.358
4-Methylumbelliferone	4692807.932	9413447.335	7950487.945	1227001.698	1144721.315	1473607.499	2655465.800	2835583.935	4489676.391
Propylthiouracil	33954322.348	61462910.254	55204455.734	109157643.588	112306805.830	133082346.759	68318567.773	60734661.298	96325608.762
(+)-lariciresinol	10500537.013	16362440.211	15499628.734	9279462.488	10036561.675	13159498.287	11419749.342	10711747.913	15757277.341

name	S1	S2	S3	Y1	Y2	Y3	Q1	Q2	Q3
Shikomol	5305912.416	9680395.299	9067675.808	1279232.162	1201136.748	1597421.386	998988.335	1057189.969	2939457.095
Quercetin	419281845.033	1021167720.151	886830816.460	797402060.621	1168470753.177	974430935.692	1104033418.427	891847260.083	1406625638.881
4-Sulfolactone	6510638.897	13236044.126	11763032.061	22265410.690	24918202.476	29230347.395	53139050.965	52741326.642	88681397.716
Phenoxyacetic acid	3248234.712	5793214.025	4962267.215	1516020.866	1932347.504	2818087.153	4060678.844	1010082.724	4046861.326
Glucose 6-phosphate	8615583.380	16389966.127	14259347.714	702285.484	649626.888	811016.065	7174487.478	7610161.863	13696005.325
oleandomycin	271088.351	15726454.300	1407533.294	3053932.114	1990110.039	1235793.433	1940451.223	1906535.397	716741.245
1-(2,4,5-Trihydroxyphenyl)-1-butanone	8370580.125	13443155.785	16805200.117	2428699.082	2929116.758	4082852.939	5037563.342	5564891.580	12764861.048
4-Hydroxyphenylpyruvic acid	9574301.962	16581005.392	15434611.645	7166439.142	7114480.652	8673607.580	22625266.165	15226078.750	27486476.596
4-hydroxybenzoic acid-4-O-sulphate	5489928.499	9200777.877	8436837.495	11115153.970	12247613.926	15573754.937	43280271.217	48648456.612	74257333.625
N-Acetylmethionine sulfoxide	269490.821	550046.021	467573.018	1859339.985	1892421.787	2415490.428	1923065.871	1965216.735	3481219.499
ent-Gallocatechin 3-gallate	279044757.431	492199243.990	450636578.845	4722933915.968	4817110176.407	6010950727.942	178455687.692	110558847.907	79928872.647
1-Hydroxymenth-8-en-2-one	11889758.461	16721796.183	16213924.615	2753625.150	1059529.176	4938059.928	4813914.090	2227514.957	11633113.900
Byssochlamic acid	49659483.550	101415648.960	83287923.641	5730841.058	5715258.705	7147940.020	38417910.328	37136370.621	61321283.705
6'-O-p-Coumaroyltrifolin	189111456.406	445244022.386	308672184.014	676654789.095	716189005.243	945991397.492	185673031.115	202958241.539	286497594.246
ditrans,tetracis-Heptaprenyl diphosphate	30967662.443	54721176.943	36075313.031	197832716.181	213552014.473	181736733.026	197400080.149	306854964.802	344385228.277
trans-2-Methyl-5-isopropylhexa-2,5-dienal	787325.688	1374195.171	3334209.763	6368591.544	5832130.224	7760778.510	4171722.931	4591108.346	1747152.606
Disulfiram	470211.364	620853.395	1049473.666	2467450.288	2375870.638	3453115.730	1125607.654	1187845.974	1916683.637
1-Methyluric acid	905498.043	1513466.750	1503040.402	4862175.579	4889179.959	6683769.699	809654.994	1061603.813	13260145.745
Phosphate	32329608.081	63197078.930	53130441.436	41371916.074	42263192.189	52783782.868	133949596.040	145270627.988	230822784.026
Carglumic acid	488611.006	1014883.325	1076339.658	2915157.740	2963464.861	4258813.175	3900049.228	4411794.035	6447052.750
3-Hydroxysuberic acid	9711586.052	17952374.680	16778568.312	14070701.907	13125630.821	17110064.724	6673403.762	7784551.729	11300419.107
Syringic acid	5722887.419	11229472.918	8242248.195	3116296.127	3544088.305	4645416.700	13240319.342	11994937.703	19383567.289
2,4-Dinitrophenol	1271521.919	6012676.735	7147121.458	2340942.940	1506611.495	2897730.898	17613900.728	6592220.423	33469619.051
Propofol	3630106.730	7831015.210	6097179.871	3514347.713	4341262.443	5776754.875	2432652.784	2736865.266	3947755.321
12(R)-HPETE	9668509043.872	3135200053.721	829261751.153	195498955.837	591499654.584	26364137.561	37972132.400	408509083.687	19755574.763
3-(3-hydroxyphenyl)propionate sulfate	14481509.657	20756225.549	17664670.169	5658051.401	6581632.906	7405500.589	7345832.379	8126689.943	12835768.549
Kaempferol 3-sulfate	13454199.616	21695847.447	24247709.766	60563981.766	64112754.088	81030741.164	70665497.005	60696840.268	96572221.458

name	S1	S2	S3	Y1	Y2	Y3	Q1	Q2	Q3
Protopanaxadiol	721506.152	1736197.952	1089778.969	62916140.990	65149459.481	62248763.989	7262834.873	7314379.403	9455541.020
Nicotinuric acid	5395491.191	9603499.620	9102194.685	367877.142	327555.275	450656.560	3942055.886	1041311.573	6649812.774
N-Mononitrosopiperazine	39158542.311	52856608.571	106659162.621	52748590.898	18107521.292	41556882.698	62169136.147	118091712.660	182230591.569
Astragalin 2'-[glucosyl-(1->2)-galactoside]	132985.276	245160.712	190913.053	24738184.332	16866660.499	19962162.291	10060797.285	10243360.236	10898415.659
metominostrobin	8639317.270	23125602.320	14614967.628	9789178.505	6664276.176	9413727.960	12612725.995	14307724.642	27477120.128
2-Acetonaphthone	2573503.249	3637184.327	2850599.279	3604255.784	4098916.148	6193414.795	7407481.327	7881255.414	13421835.378
Picein	41380307.803	8985048.076	11236365.418	9556969.702	32249957.005	2452315.364	15718285.759	9345726.958	42372532.032
SCHEMBL5940872	7355052.823	14529640.222	12242103.712	3730571.485	3937011.842	4594091.375	12476519.035	11737897.281	19901156.702
Ethyl biscoumacetate	523956.976	3339672.836	977833.372	1725701.161	1718596.743	1518850.793	18433262.577	12354603.755	20537095.770
Caprate (10_0)	1535617.363	2906401.900	3040360.343	719909.870	868052.503	1073952.963	1508843.786	1826697.374	2370103.189
3,4-Dihydroxybenzeneacetic acid	17426789.151	32235686.386	31192707.493	9265291.229	9694376.240	12367484.663	13257436.306	12732700.883	20866434.044
Scopoletin	103664816.204	186892449.963	167458427.830	10631798.305	11268048.695	14407875.316	97244895.594	98619924.578	162355496.652
Caffeic acid	39406910.034	34309168.731	63550820.274	41400836.343	23306701.833	26178821.933	34340803.192	30470446.571	90919146.792
Vicenin 2	3163827.067	6336649.758	7187881.634	4752085.255	3954489.471	6550890.195	12938973.497	7732138.421	12948604.325
desmethylastemizole	537931.017	1643109.313	4686686.077	600398.794	786920.615	845565.102	8020208.535	7091360.162	10287816.354
Pikromycin	3080643.992	4828368.712	5278617.624	21888314.116	22699771.834	29352417.425	25884535.500	15273681.452	23033367.617
FA 11_1	36539533.619	64465615.782	59376400.460	236392634.456	261985413.820	342389044.457	431487565.501	421117591.653	682055743.680
L-Proline, 1-acetyl-4-hydroxy-, cis-	143986.309	381140.136	186787.969	585443.996	400446.982	556760.558	2424323.594	2726968.733	3876211.242
naphthoic acid	1685489.713	6320128.346	3066911.244	653732.215	853527.332	808025.687	1910816.050	2126466.495	5041766.554
Swertiamarin	1413279.117	2747408.840	2549882.565	1221587.239	1168116.073	2708929.505	793304.303	796007.762	970121.654
Prunetin	2294752.959	70885707.361	16583253.601	2331551.487	12047476.682	2660421.890	12464008.509	1085371.536	21683440.397
2-Propylphenol	3330948.393	6288322.075	6030187.700	2634974.017	2627057.842	3554389.553	1282218.278	2369733.224	5376461.869
Rhodovibrin	204766.119	1053294.057	232288.956	11345771.732	13233555.064	14326640.645	4544856.316	3908490.994	6266101.967
3'-(2',6'-Digalloylglucosyl)-phloroacetophenone	11660273.247	14598972.130	18379788.676	35691672.144	41614138.184	50918447.352	14789261.107	13191689.360	20158354.635
Tiglylglycine	42049756.280	79911102.904	71587217.985	26373720.231	11018747.511	30161259.416	82797924.537	83280321.534	137598389.120
Xylitol	12052159.026	23039796.341	20508692.442	15707154.108	19007705.472	26248970.830	15673431.849	17419631.300	26248005.088
Bellidin	392616.754	1559922.105	4099339.126	10602447.008	13916314.244	4738400.490	954141.601	527296.678	678937.186
PA(16_0_16_0)	55934235.578	440496843.551	270997127.896	859843328.238	382079063.606	376460311.608	1725247927.046	323975078.176	393936784.234

name	S1	S2	S3	Y1	Y2	Y3	Q1	Q2	Q3
4-Hydroxycoumarin	3378870.112	5157748.692	9803837.422	14634630.173	14658791.902	18987470.790	68226297.468	70837458.266	119040041.761
Isocitric acid	24945656.559	50506114.950	43540479.328	110328796.162	104783842.475	166423712.385	81864370.771	79712669.717	135203025.445
Kaempferol 7-glucoside	2669517.050	2492149.740	2014379.878	1822635.699	2620618.837	1953639.466	5948699.384	4346515.368	4638365.300
10-Chloromethyl-11-demethyl-12-oxo-calanolide A	32920697.907	25294336.800	41108573.434	27240669.355	25335655.723	9165553.501	31074703.784	27513255.402	41728627.242
(4S,7S,12Br)-6-oxo-7-[[(2S)-3-phenyl-2-sulfanylpropanoyl]amino]-2,3,4,7,8,12b-hexahydro-1H-pyrido[2,1-a][2]benzazepine-4-carboxylic acid	4806260.506	6700290.927	4387818.133	14378585.281	16711782.844	21860818.833	10892152.905	11387828.334	14003865.871
Picrocrocin	76437341.354	156676197.240	137099403.488	121914333.631	130141075.046	166234925.204	170145043.649	176328279.262	289995949.839
alpha-Monofluoromethyl histidine	11612061.637	24125258.501	20282148.968	12887586.209	13445796.811	17593030.260	73173463.719	3731417.858	108115704.273
Benzoyleneurea	58987488.344	148543407.972	100650291.638	12726206.783	13673044.335	17548153.413	77663124.518	75190994.531	124335187.091
(S)-4',5,7-Trihydroxy-6-prenylflavanone	254294100.939	615378446.515	543689465.448	265208885.890	302700933.415	318432584.919	392739147.484	193356211.450	640053044.430
Pimelic acid	2318613.987	4571265.301	5570587.847	1016017.663	152137.279	359798.907	3349931.162	4214190.668	5010442.577
Glucohesperalin	31640808.427	73827665.766	13840629.304	23658410.123	26622487.034	12086718.162	29936969.113	36863933.720	137418619.832
Hydrocinnamic acid	25712954.676	46126014.622	41388031.465	3103272.891	2452753.478	3880018.531	43025010.103	48280469.945	75050844.380
2-Galloylglucose	125162270.786	260197928.447	214044909.508	7245071.570	7722153.539	9206930.816	8780300.120	9266813.360	15206832.549
Phosphonoacetate	7567750.317	13621075.692	11274414.247	10255912.870	10593797.730	13574311.323	31369815.247	30773407.372	49077438.683
Daminozide	1709689.926	2764014.012	1441811.964	260967.874	175230.843	232986.435	3079280.013	2942545.081	6595981.989
Nicotinamide N-oxide	6482056.502	11659194.012	10973790.534	4727970.912	4138685.716	5358641.304	9635601.571	10489203.696	16280857.351
Kuwanon Y	463346.670	1102898.789	2875834.898	13687054.852	4926079.371	14846840.497	4598180.881	18165824.226	25425990.289
Benzenebutanoic acid	58457205.237	110942065.412	111550256.131	12211247.110	17718261.970	15757995.881	28959297.081	28813215.897	47655583.639
Momorcharaside A	76151296.921	24077632.279	28302125.459	327532001.444	467203527.933	146693938.389	55835924.543	24334060.945	57382100.638
5alpha-Androst-16-en-3-one	2252686.955	26988536.298	20719769.186	27355832.804	30560669.784	35323026.357	16008724.958	2514333.860	32409624.099
Dolichosterone	50987856.598	94339347.339	86989154.411	91512127.104	91985182.381	135535907.281	76634404.716	68193625.731	60291639.414
warfarin	1332822.868	2671444.968	2236022.473	666012.243	1041800.247	1330181.785	554193.304	428033.209	2807667.923
Hymenocardine	21536370.631	29392809.524	13216693.559	13419884.377	19446339.911	22914526.873	32536897.152	28921587.772	13176924.851
2-Amino-4-nitrophenol	84210739.426	165496073.305	152327080.870	120412079.106	117466271.688	181578965.187	987558533.928	996709791.203	1702767597.621
4-Ethyl-3-(p-hydroxyphenyl)-2,2-dimethyl-2H-1-benzopyran-7-ol diacetate	10610626.955	32985765.092	28367263.068	12382616.095	13761314.554	12522497.203	22341719.824	8611523.932	38921124.636
6alpha-Hydroxymaackiain	13300238.467	18124644.744	20899974.366	11383266.149	12808708.031	16344117.802	5743638.845	8276545.729	8219653.736
β-Obscurine	769780.552	341007.191	287962.702	197487.630	198108.731	220789.183	19997.024	36082.292	693769.943
N2-acetyllysine	247252	446617	392466	618753	543788	722691	129297	134509	232680

name	S1	S2	S3	Y1	Y2	Y3	Q1	Q2	Q3
	353.417	553.773	259.107	03.501	45.547	78.802	357.281	665.362	894.349
Spinasaponin A	167865557.443	53819166.160	31936531.208	75970302.455	139515902.993	228979556.294	74090399.167	68928787.055	131650405.598
24-Hydroxyglabrolide	1222904.036	2471166.562	2899519.144	666166.195	852365.816	1235886.180	774021.857	723966.915	8340013.714
4-Hydroxybenzylamine	857970.686	2064613.861	1443525.182	11681.848	235218.686	199212.144	653744.190	1082924.177	1413401.272
Gypsogenin 3-O-rhamnosylglucuronide	314362.479	4648359.664	64633.116	22520472.053	12388852.461	6373905.258	2996722.719	2300420.548	2977852.734
Nitidine	2288647.035	4108290.843	3875987.815	1065671.433	1177764.399	1578661.220	4009256.408	3655892.829	6620397.626
Squalene	448882.151	761478.874	641127.999	400931.225	450122.157	477868.037	338994.964	299348.408	658484.721
Gallic acid	40293712.098	79252766.017	70260473.023	92792648.918	23604089.277	121978371.840	16434875.750	7235698.640	132227020.905
1-Hydroxypyrene-7,8-oxide	3332773.609	6609926.665	6110155.958	374471.557	1593198.665	1852144.136	3623176.177	4514742.661	5985477.849
(-)-Catechin 3-O-gallate	164373635.670	330848343.739	314669571.488	948559842.702	985531037.827	1234518531.589	10709094.299	12923778.340	18145184.702
N,N'-Di-1,2,3,4-Tetrahydroacridin-9-Ylheptane-1,7-Diamine	9029289.924	24590683.475	16098847.100	722483.954	1385736.403	683027.444	4106185.304	5999932.342	7793640.488
N-Acetylserotonin	1364092.355	2163472.632	1798158.905	1711304.210	1776520.268	441383.816	731771.421	368284.968	1597943.623
Hydroxymethylphosphonate	2482640.592	4883080.719	4844263.409	1854974.479	2769255.610	2240247.530	3722415.233	3729591.781	5837436.129
Sorbic acid	1141180.822	2940250.419	1972978.170	501571.319	2248447.404	817221.744	1419945.209	1902649.666	2398983.150
2,2',4,4'-Tetrahydroxybenzophenone	22575919.382	42393542.101	35758281.664	204341806.001	219966627.441	283545048.367	59720736.993	61739332.348	105069761.931
6alpha,8beta-Dihydroxygermacra-1(10),4,11(13)-trien-12-oate	5353076.848	10482657.971	9363299.846	2922828.819	2911488.456	3900801.749	8375048.243	9250110.705	14426326.537
2-Hydroxynicotinic acid	16254547.979	30935923.918	27944435.554	845065.458	4426054.930	916130.518	68530608.000	123058540.776	138159789.338
Proxyphylline	6387865.650	14474153.743	12373035.864	9358592.074	11095251.327	13714696.460	11977074.071	16632452.670	21715373.703
(2R)-6,8-Diglucopyranosyl-4',5,7-trihydroxyflavanone	2976998.600	6301750.971	4751811.661	11149453.042	6160240.313	7872841.748	971901.513	1048319.656	1648871.042
3-[(1E,4R)-4-hydroxycyclohex-2-en-1-ylidene]pyruvic acid	906137.773	2952896.598	678072.803	1393420.218	1393940.897	1777558.336	808355.608	789146.079	1309947.255
tuberculosinol diphosphate	6476985.544	21178068.190	27400456.735	259883.389	161948.881	257191.193	5610386.188	9013166.905	3786780.246
N-Acetylglycine	113441867.369	311250459.794	332117245.602	109207313.417	114032166.540	74351267.556	83510525.920	39796926.085	78943014.309
Momordicoside K	5755987.747	3410583.439	3693150.726	874152.279	405080.348	1447471.390	2097185.567	2521677.120	1303998.809
1-Hydroxy-gamma-carotene glucoside	1789469841.122	627646469.748	149846026.015	536953038.111	297684471.541	28069955.352	94357482.525	111349109.705	42843620.312
4alpha-Hydroxymethyl-4beta-methyl-5alpha-cholesta-8,24-dien-3beta-ol	7665648.669	16184925.578	14374763.725	5622549.554	5924451.816	4088773.289	7780004.785	8333124.785	11225273.329
4-(cytidine 5'-diphospho)-2-C-methyl-D-erythritol	3292064.991	9149820.519	8207470.996	20357286.563	36277859.984	37290805.983	687614.838	497759.004	527867.420
2,4-Diamino-6,7-dimethoxyquinazoline	4378827.390	8273419.523	7423724.917	2999802.921	3291192.225	3998943.629	2317188.854	2337588.593	3500687.750
L-Norleucine	9399072.380	20118607.885	17602013.429	59519096.714	43690061.530	8433677.971	87374038.123	11932887.141	85371532.084

name	S1	S2	S3	Y1	Y2	Y3	Q1	Q2	Q3
17-Hydroxypregnenolone sulfate	17954084.183	35393130.191	30510357.791	2767742.602	2528423.875	3708556.373	4892959.241	4804052.272	8517369.084
Chloromarmin	57409165.398	607013654.363	520895087.919	6904783.615	7864857.036	9376803.756	3998427.934	3789896.659	6333340.234
Eudesobovatol A	352495.294	674479.503	186606.195	23874449.076	33499217.107	37511757.232	5278997.986	7790500.703	7782066.732
Coriandrone A	856762.138	1488719.649	1381167.078	211682.931	297309.329	228647.106	7900143.633	7791294.712	12458425.125
Estradiol-17alpha 3-D-glucuronoside	5138362.856	2371794.643	2374837.718	1970007.236	22880388.655	12527077.601	2854690.474	2206467.598	2053813.680
4-Hydroxyphenytoin	2882921.055	4141332.225	3464111.059	8760319.450	5564195.201	1804034.604	2893163.583	1241211.743	8867803.236
3-Epinobilin	4816660.119	8358038.571	7820886.176	15995219.630	1512666.749	21469021.606	5663225.480	4268854.176	7204289.096
gibberellin A37	1512326.145	2067617.613	2760515.044	3434822.157	2917775.723	2443036.806	9995820.345	10769047.421	4278843.112
1-(4-Hydroxy-3-methoxyphenyl)-3-decanone	4727397.735	9095139.967	8334807.241	20497578.240	13140260.577	16845632.383	3671224.300	3273384.445	6069385.450
Evodiamine	1566149.424	3131111.617	2330911.890	999851.660	854877.170	391751.789	5925899.556	7332588.007	6432974.916
2,4,5-trichlorocyclohexa-2,5-dien-1-ol	422590376.754	807310510.676	694537984.008	16349803.629	13460378.518	18063823.027	35432888.388	32750281.734	52454387.017
streptidine	4860441.471	9871556.748	8965140.223	5554043.701	6068267.674	6693585.479	7824661.910	7542481.185	12688201.735
Pioglitazone	11150814.410	17277293.708	18524752.054	33690273.324	35186216.106	37660148.559	15960275.510	21302729.022	31929423.008
Nicotinic acid mononucleotide	42784993.206	83809888.889	76133378.019	311244274.808	324825349.603	449791379.215	22514132.090	23015877.160	37495647.464
Magnolol	45673934.942	89499445.822	77440688.406	25316602.434	26640036.597	32988359.551	75355986.276	78182973.042	127596778.449
3,5-Dihydroxybenzoic acid sulfate	9657872.333	19548239.647	17026245.985	40165478.401	44394116.617	53663292.522	17993516.123	18870206.645	31279080.202
beta-Cortol	19910425.313	36348409.344	37318316.151	7412812.941	8803722.826	10385941.462	15307664.768	13121928.847	21974535.857
3,5-dihydroxy-4-(sulfooxy)benzoic acid	1502809.806	2592875.951	2924016.306	2540673.702	2960907.590	3742618.311	8840278.414	8708683.470	12958059.607
3-Decaprenyl-4,5-dihydroxybenzoate	412736026.641	243258966.246	14461977.182	34990503.360	42217003.238	19082016.843	97454988.124	45495673.448	15206625.781
Parthenolide	12224095.102	21461964.121	17658393.304	853183.367	1024488.936	1137011.770	21700928.764	22636040.673	37206219.559
4-O-Desmethylpapaveroxine	12508586.580	27749138.453	82932417.572	79191668.641	76348039.070	80469154.748	37182058.778	145317830.484	242208714.241
Galactinol	1025394.256	2004044.787	1679857.831	1262145.750	1241525.867	1652345.449	4665332.814	4407095.798	7109518.539
6-(Methylthio)hexyl glucosinolate	15281999.980	30394721.364	28008167.200	11217166.265	12083583.457	16836809.184	4757776.799	5355491.764	8279291.190
Populnin 3-O-rhamnopyranoside	3847373.735	5110116.126	4435446.716	5225947.043	5174360.622	6276829.886	4058578.564	12307935.299	17397047.370
anthracene-9-carboxylic acid	1370957.744	2213692.288	1984340.116	3241060.618	3350758.614	4316383.103	3965677.101	3446084.152	5750535.262
Geranyl-PP	5702342.277	4234648.636	4890122.726	4885067.059	5744785.421	7879871.697	2442980.189	1625598.037	3646564.397
AdoMet	313756.100	2049051.955	1953410.768	486888.354	461872.531	730268.350	1378499.725	1663719.218	3238563.868
Ginsenoside A2	2803827.616	6614033.218	154560.090	7322129.020	6168818.904	1680530.607	3765425.460	5453154.909	3070869.087
Spheroidene	130669	393303	315657	150312	128454	552281.	127657	157273	137633

name	S1	S2	S3	Y1	Y2	Y3	Q1	Q2	Q3
	91.189	07.125	90.450	5.103	2.522	205	99.249	79.550	64.638
Quercetin 3-glucosyl-(1->2)-galactoside	5629443.412	11057027.830	7541158.723	20351541.113	19154332.163	22719102.021	7611725.994	6883408.219	6883764.078
Ciceritol	44333.735	175294.572	785806.047	2219267.448	3970521.940	5592720.212	5448542.343	4827290.979	10762934.845
(-)-lariciresinol	6130905.684	11640282.238	3048678.321	34703216.823	30036055.622	39266727.216	2127680.072	981102.155	325568.232
L-Threonine	2987934.897	6163259.529	5426292.385	3987379.903	3703536.016	5053447.789	3417749.438	3903064.747	6003966.467
D-Arabitol	241358511.249	343361121.639	297230880.128	38901657.462	40188819.875	39407192.866	437994272.563	73964756.289	599033529.709
Dibenzothiophene	33106493.378	81434366.074	55780076.271	5419968.160	1690180.008	2220453.339	19357356.735	5055090.368	6850080.461
Guanosine monophosphate	2884386.270	6189338.074	1422607.041	860465.203	37752.052	220406.019	3613102.281	3469767.610	6349765.935
Pantoate	659523.216	1543158.569	1406748.672	1362700.902	1350569.144	1475084.776	3132936.189	3410419.860	4341550.533
4-Hydroxybenzoic acid	6009206.781	10109154.488	8804826.489	10926365.338	11328956.951	14857156.820	19795011.616	18083293.486	29210573.305
Rhamnetin	7821190.041	15662344.238	13422235.340	24541792.972	25639736.244	27915883.151	6924000.038	6885408.443	11045269.261
methyl aklanonate	7657632.907	14616579.939	12432183.200	25962036.992	27316446.276	34252624.543	2835202.985	3036656.152	4715031.528
7-Methylxanthosine	1386834.036	2388385.188	714192.389	2362226.903	2248743.379	3269353.935	604714.222	906548.400	683002.376
N-acetylneuraminate	621474.671	1618257.709	1541515.164	3274130.020	10733625.665	7330542.342	1836344.073	1905136.190	2770880.458
Harmaline	17451907.124	32903630.476	33414331.397	1514517.816	775471.574	1907567.469	10062501.003	18795440.594	18969711.356
N-Succinyl-2-amino-6-ketopimelate	3578151.188	7321414.919	6198699.262	35328658.817	37492782.289	44402953.453	1817377.556	1812612.130	1207219.327
Dehydrorabelomycin	2036986.566	3959410.505	3610989.804	2689351.067	3012866.890	3261668.332	5400365.457	4818416.177	7201875.970
8-Demethyltetracenomycin C	8068116.609	29723834.174	34470268.483	260966768.827	146046396.416	261621593.528	9955190.101	4608131.863	5142513.841
Adenosine monophosphate	7099965.261	14564323.607	12873277.162	5370560.451	6593070.122	7892044.861	19046284.397	16522021.871	25923528.140
N(tele)-methylhistaminium	2793624.205	5810752.291	5164766.549	74158.807	183008.156	120430.122	1804277.877	1915314.471	2205237.059
5-Amino-6-(5-phosphoribosylamino)uracil	6880394.070	17793174.794	12884096.845	2736375.898	3196541.723	4613254.064	3224149.237	1988127.664	5709198.290
2'-Hydroxydaidzein	2987848.544	4891248.742	8769398.103	3891101.412	3669393.265	5136838.180	7195636.508	3658791.451	12396729.292
Bergapten	20168351.766	35239199.904	33011309.139	4397331.613	3641398.492	6005195.156	20897155.368	15838212.798	24246754.114
4a-Hydroxytetrahydrobiopterin	16098660.422	8725619.372	30838040.906	17152649.846	16182746.840	37193868.191	29228514.489	30098689.281	59409253.530
Sorbitol	11973602.676	21726461.997	19223183.096	3801349.959	3601706.430	4665279.299	14507450.711	14572056.816	23028014.415
(R)-5-Diphosphomevalonic acid	212748.075	299806.196	248872.084	1013429.058	343135.359	1515363.577	4255417.413	3533127.350	5588667.281
Melatonin	49772015.624	100903988.239	94169324.708	17512202.817	12150714.910	24571965.506	8771220.923	7710928.881	52529749.274
Glycinol	6225406.781	22609481.157	10004936.754	18542925.673	18852935.114	23374246.949	7250377.644	7473105.469	13609765.560
Harmol	1421376.773	3326850.855	2270691.980	11767852.484	12177882.886	15478026.790	22133967.608	26899854.601	43973241.381

name	S1	S2	S3	Y1	Y2	Y3	Q1	Q2	Q3
Hydroxychlorobactene glucoside	388330344.546	8898009581.059	215412673.362	21400223.225	39328536.101	8308832.130	17718059.390	100477801.189	51752960.077
Diphyllin	66939927.854	114332155.036	107779689.169	824946799.730	899154517.059	1110816006.963	14694594.558	8027720.148	12867594.762
6-Methoxymellein	786029.100	10426263.729	10398225.818	827048.972	879716.205	1496605.990	1168877.801	1613464.975	2852703.958
Amidinoproclavaminate	1335353.375	2460586.182	2106800.893	722469.152	1248887.443	1441967.279	1715795.724	1803454.612	2854776.218
5-Phosphoribosylamine	86462613.494	152351979.696	125219862.381	1621976325.390	729505471.956	408320300.335	22513826.645	12842356.601	18284210.600
Ethylnitronate	997749.303	1513452.866	1436011.136	189894376.441	197279419.039	221532705.823	1497335.819	1036743.861	1453286.553
Orotidylic acid	1900574.395	3085148.771	3980030.624	31550360.686	31100555.826	39541031.780	683720.016	919605.695	865246.833
2,6,7,4-Tetrahydroxyisoflavanone	1172885.778	7733136.981	8417072.599	3885707.353	4390362.544	5970954.780	45818672.361	64962050.580	62459250.004
Daphnin	4178928.620	8440965.743	6988998.532	16568736.346	14771208.310	28424506.042	577713.874	2674161.197	2504326.399
Leukoefdin	6011283.400	19680723.240	9845507.486	122791989.807	99466695.718	153187653.654	1184698.993	5505132.583	4512042.902
D-Xylulose-5-phosphate	717535.018	761766.271	1119999.293	267658.729	220760.539	310296.188	1661746.557	1413576.585	1096028.030
Porphobilinogen	1027528.513	1069647.065	1717178.303	924954.019	1310509.415	286813.476	3354532.051	2733016.097	4684350.216
N-Acetyl-5-methoxykynuramine	630596.822	1127453.088	857238.808	2772144.208	2780720.503	3535777.192	577337.935	566235.973	768261.639
Oxyanin B	17649307.054	19855164.498	16260078.130	58943477.386	66721418.012	78007409.696	65312275.712	83068474.174	143517742.219
Dihydroconiferyl alcohol	2501556.796	4880883.864	971563.476	1328294.484	1545533.563	2049012.556	2878960.297	2775570.200	4754492.908
(6E)-8-hydroxygeraniol	16319640.118	29968905.509	25960413.989	316189.843	311246.924	288177.269	4849646.632	7776753.132	7867941.851
5-Methoxyindoleacetate	33300573.224	60972169.305	52667186.371	27716064.566	25770760.513	35287448.816	18824538.909	17478334.575	29342955.359
2'-O-Methylisoliquiritigenin	985839.123	1410344.085	2256947.618	938678.510	950351.028	1231353.849	1174051.499	1209982.857	1820853.795
2-Hydroxypseudobaptigenin	4921385.192	9884003.638	9034637.930	10174874.774	16179287.778	11537004.238	12676549.593	9306279.281	23997263.627
Vanilloyl glucose	13436866.648	25476082.693	22530858.925	31832859.347	30350130.578	41725527.120	3447169.614	3312299.923	5718411.107
2-hydroxyisoflavanone naringenin	341974.442	1265533.025	1120868.609	1249860.960	1288942.279	1672953.556	1320829.646	1065267.699	58189811.042
N-acetyl-alpha-D-glucosamine	3273688.455	5692782.363	4773921.839	43280525.492	45485314.549	58914361.920	4041689.924	4136815.025	6078898.404
Patchouli alcohol	2803377.763	8231087.453	4847911.987	2500921.684	2590471.783	3748652.100	1898023.050	1850427.646	3184438.934
SAICAR	7155069.694	12193002.602	10568554.632	19856194.994	25375263.878	34900859.374	11167953.792	11554619.644	18528414.194
Plumbagin	4384358.684	7948654.757	11437652.983	429792.324	364823.365	482891.798	3100763.602	2429403.743	2893788.699
Syringin	837066.817	1573727.541	2231162.842	16173756.448	16607241.084	19029773.716	8678822.436	6484323.791	11468269.777
Deoxycytidine	6416593.418	9792051.009	11049244.233	15298181.306	17003215.612	21345042.481	5178256.553	6690443.887	9508598.916

name	S1	S2	S3	Y1	Y2	Y3	Q1	Q2	Q3
(-)-Arctigenin	5795500.450	11440415.198	10458364.660	1032079.818	1311101.698	602322.664	8894827.216	11395010.856	17100254.028
Purine	29419635.891	56897890.129	49874559.609	37914418.643	38989858.068	52364665.096	72864583.731	72886805.587	119500162.842
benzoate	1374556.522	3776765.131	2125648.279	232147.254	206714.954	298903.316	789245.249	691306.833	1220780.469
L-trihomomethionine	3849971.188	11387189.212	5601678.408	7236315.653	8987214.772	10125250.710	5219194.866	5889164.136	7190021.053
3-Methoxy-4-hydroxyphenylglycolaldehyde	2931930.636	2225237.727	3068292.879	285606.176	854216.739	621919.475	3524758.730	842004.622	5698758.854
Ricinine	17775949.967	8922965.581	28239175.430	31048255.858	17123083.110	3718373.931	32579383.373	58280331.733	9342127.403
Lacinilene C	16721319.746	31494343.431	28201438.382	48283326.530	48608097.440	62922258.301	48612977.439	51872016.910	81389840.571
Kievitone hydrate	7216258.390	14073026.592	12946758.088	1900156.388	604832.700	918616.480	5279096.226	4850485.876	8741379.550
Aurachin D	26078637.012	4189472.684	26217184.653	139976735.034	124221296.390	35407913.755	174658922.423	174618449.141	89693021.382
Legumelin	3810343.000	8220512.912	6498398.319	1572970.430	7493296.910	16945361.679	21307003.528	29603156.083	45174739.256
Aesculin	2534658.419	4207185.560	3927052.445	8508667.742	8530697.500	10150502.495	1500787.349	916443.738	1659471.398
Casticin	2216884.936	4252979.126	3963463.980	11765686.796	10848470.841	13835724.247	164063.712	319846.191	315641.256
1,10-dihydro-1,10-dihydroxyfluoren-9-one	1169224.386	4988338.638	4856704.852	1046277.063	2079388.327	1419216.957	11340012.748	11717826.564	18978512.120
5'-Hydroxycotinine	5747523.388	16944696.186	15821863.033	7627517.512	7814059.948	9870977.458	9062167.427	10211890.687	14768756.927
D-Pinitol	8182447.819	28042042.430	21668632.339	23660372.873	6101599.952	17240675.937	22664934.459	44148730.531	108871418.437
Primeverose	88267579.936	167216401.722	143867806.145	586107452.412	628929756.932	823263776.514	896542130.624	900388991.776	1542753216.501
Aflatoxin M1	2025424.462	918983.701	988517.862	51949645.652	30890881.703	36001020.827	2544895.664	2783655.143	3700392.643
Protomycinolide IV	963066689.851	1338750120.292	2412734780.181	401608220.596	257971194.443	48538760.189	389534729.871	255024073.048	196460817.457
Rotenone	59323.049	267488.354	289550.767	17738996.729	19669571.900	24810499.908	28683833.325	30585340.459	50578051.497
D-Glycerate 3-phosphate	646959.736	1465779.737	831169.199	1452214.781	1437168.154	2322104.716	5377966.359	6607158.485	10740485.899
N'-Hydroxymethylnorcotinine	413690.079	1213915.976	712873.188	1997149.421	1846308.568	2560011.701	1218432.145	1393777.554	2160699.162
Mannose 6-phosphate	4853774.495	7840745.153	6886480.479	76889966.283	82981236.036	132640814.021	234551.216	151760.901	107579.201
Vanylglycol	2607562.086	4984429.351	3908322.715	787492.329	599398.573	780219.070	1095200.495	37957308.109	67655365.418
8-demethyl-8-(2,3,4-O-trimethyl-alpha-L-rhamnosyl)tetracenomycin C	1396571.121	6262322.972	4021550.541	1108022.208	3718442.788	1967699.613	5081334.825	4857954.043	18617526.942
Methyl 2-hydroxybenzoate	5057874.257	9123787.633	8146117.910	3182147.979	3633920.881	3725209.863	6474789.262	6850881.371	13007954.723
Alloepipregnanolone	13169604.460	11784930.575	28598750.306	13011732.397	16302263.290	5955875.026	81689303.597	20837806.970	116657593.618
4-Methyl-5-nitrocatechol	2730425.928	3563573.522	5348716.683	3275222.086	500007.352	4394850.925	10962642.461	19283087.462	23269744.362
L-Formylkynurenine	114530	255674.	229626	107565	121154	318109.	388312	402760	612423

name	S1	S2	S3	Y1	Y2	Y3	Q1	Q2	Q3
	5.771	388	1.955	1.709	0.518	343	9.845	0.717	5.790
7-methylthioheptanaldoxime	2843967.526	5348684.636	5268815.045	804119.020	975775.945	516507.111	5671478.590	4895163.516	9526705.495
D-glycero-D-manno-Heptose 1-phosphate	4382130.364	10776935.396	8822417.351	1649975.921	1547474.992	604276.559	1933505.818	1945761.307	2890748.699
Bergaptol	186363.700	3551006.701	4961890.613	2530264.291	1754266.024	2672800.922	2936574.783	361988.324	778246.796
Coniferaldehyde	132141093.856	224110707.504	197734983.454	12779434.541	12189235.953	15909835.903	57069633.445	60631073.560	89709692.631
Dihydroclavaminate	1820470.187	3520365.563	3311273.395	5594717.976	5970609.146	8311754.531	7257753.716	7644534.043	11285513.518
Fumitremorgin B	16658351.689	143072931.255	118879271.699	50800356.664	54632027.965	66453535.819	13619597.872	28787466.279	17734758.182
Aesculetin	30041616.934	61761449.633	52373212.622	2250350.693	2193215.852	2836606.673	5266832.568	5193510.837	13475125.018
Juglone	11762842.338	22762290.317	18824825.058	4632904.884	4613658.280	6136607.269	2416058.484	2800542.968	4097643.271
Patulin	5312400.206	10037621.378	7238983.191	6982128.680	562664.231	9027004.717	3798572.289	4298094.629	5985369.805
coniferyl acetate	9077759.495	15909379.679	12977370.978	3861095.458	4034355.072	5078519.362	526349.729	328233.306	724951.782
Menadione	1952231.269	3753615.120	3313714.372	4818119.268	5224158.116	6387189.362	1222422.268	1234206.236	1802097.187
1-Methoxy-4-(2-propenyl)benzene	4661077.028	7243386.351	6800550.488	873231.597	903563.541	1552196.762	6646180.020	8215280.556	11142973.279
Medicarpin	707683.131	1396877.960	896390.082	3624801.560	4052207.900	2630885.174	247557.099	66469.140	446155.866
DMPP	26196084.836	51322861.218	43796991.200	19817352.124	19806918.356	25417798.210	39401593.473	36150898.926	59963601.423
1-deoxy-L-glycero-tetrulose 4-phosphate	8310999.734	16665808.370	14001602.497	3062738.811	3344225.808	4466560.204	2829154.663	2529723.956	4177367.439
6-HYDROXYMELATONIN	21401372.061	39049982.547	50730343.269	17279951.896	12651008.928	15566454.854	2065183.485	3062918.660	3510294.147
Indican	39932832.146	73106089.554	66691141.562	106419543.219	111409482.051	136579264.386	15290546.022	14999898.328	27624907.530
4-amino-4-deoxychorismate	8071166.200	14068696.764	13258751.993	3380146.012	3601781.946	7886742.279	9421005.930	5273175.878	8133232.551
Chorismate	7323808.922	14040723.629	17707334.963	5692340.376	1184339.071	7067034.226	14938743.276	35212086.066	22101899.435
D-Lactaldehyde	14492468.582	29162919.579	27518088.362	155479275.971	168426501.353	200636969.922	93842273.900	102296718.990	169256123.995
3-deoxy-D-manno-octulosonate	11083575.250	21213096.878	11493668.754	85278627.750	89181122.966	108520240.149	34163956.911	35855622.529	54367340.321
N2-Succinyl-L-glutamic acid 5-semialdehyde	3804390.924	11512827.985	11944656.287	18477515.572	20064040.424	25634209.242	3582681.834	2602278.532	5579471.056
5-chlorobenzene-1,2,4-triol	86042675.965	151621682.774	141399107.858	80503752.873	78445568.864	93703597.009	128526653.251	129348927.862	188277423.934
3-deoxy-D-manno-octulosonate 8-phosphate	20160401.113	43178001.775	36424517.645	6815254.255	5466573.302	7915325.975	49699274.562	53826688.695	84705313.594
3-Indoleacetaldoxime	2042808.193	3662169.349	3542879.097	3528473.668	3550396.326	4451133.290	3649384.568	4191170.258	7180946.744
biphenol	3621500.510	4522323.843	5171447.212	6104665.207	6191489.531	7916431.063	1305240.564	1553025.685	3237387.066
Oxoglutaric acid	9382619.694	21392818.113	17604696.540	29172925.689	30818886.890	38012755.332	26846894.735	29513450.963	42584233.922
Pisatin	1126191.928	2128381.833	2268304.752	144090.298	130641.082	213386.767	13254417.422	13675940.552	21337356.518

name	S1	S2	S3	Y1	Y2	Y3	Q1	Q2	Q3
Aminoparathion	275590.445	510371.218	405417.726	220107.085	2511404.283	3403364.618	372963.693	795673.377	1864211.507
Pyridoxal	6581890.805	11771884.634	11231967.400	5437292.114	5917480.506	7589739.599	8049659.026	7241240.725	15568865.187
3-methyl-4-cis-hydroxy-2-butenal	6473899.796	10048513.150	8576780.133	5667969.915	5512978.869	7269118.486	4422248.250	5272327.990	9043688.964
1,2,3,5-Benzenetetrol	48610518.340	91866269.990	83909432.157	24129299.771	26055315.094	29369521.386	136676619.148	141567066.626	228277241.310
Imidazole acetol-phosphate	1417000.235	2547852.222	2236278.382	1853608.583	1886546.301	2159813.709	23684577.733	24817882.291	38467463.527
C.I. Natural Red 20	4918914.453	9278194.668	9984559.944	5809866.812	6148421.439	8149618.148	3770062.950	2981489.979	1501932.853
2-Formylaminobenzaldehyde	2190704.551	3772525.316	3541321.865	127525.476	502353.275	316597.209	2912878.875	3104691.848	5704426.946
Phosphoethanolamine	12456054.146	24210821.117	14858827.262	10775098.275	11380931.803	11096901.434	6858198.706	14827715.975	25767629.656
1-O-Sinapoyl-beta-D-glucose	11657134.260	28779741.222	19379273.269	23964037.342	24448412.358	31579401.685	4832815.449	4469702.846	6902951.077
naphthalene-1,2,4,8-tetrol	12763124.048	21766926.111	19496692.853	7052866.551	7364357.107	10147329.681	9825005.991	11195863.723	62963815.723
trans-p-Feruloyl-beta-D-glucopyranoside	570883673.211	1049387721.651	811031007.145	1214376608.965	1101695321.752	1590994384.520	257501037.363	272347025.088	425841815.100
Isopropyl catechol	1789750.226	3763985.626	3342649.835	1870617.028	2115005.556	1983381.828	2774614.907	3083222.675	6699878.572
Mevalonic acid-5P	138843.168	305751.313	1054348.486	294634.791	363654.674	389619.086	118286.750	700490.607	198152.782
2-hydroxylamino-4,6-dinitrotoluene-O-glucoside	969136.408	9060329.096	6523602.601	4760176.908	4011296.649	5710432.242	8633899.343	9231634.934	16556208.712
Trigonelline (N'-methylnicotinate)	248059.749	488979.721	424187.001	9962895.824	11214127.678	14899764.311	6479645.510	5831736.652	10482947.116
Dibenzothiophene sulfone	321814458.754	650401925.813	574196210.719	16257219.311	17627177.569	19385546.894	66048281.969	73920770.420	386015480.147
8-Demethyl-8-alpha-L-rhamnosyltetracenomycin C	2316600.720	5057514.524	8777539.431	33825637.566	18703661.998	27088451.430	447226.814	499760.528	781846.250
Melamine	5272637.108	10050226.715	8799585.054	5721887.088	6001133.229	7715973.607	2723366.173	5729218.343	11689226.884
Biuret	4340636.905	5790881.045	6751276.033	600880.866	644074.213	3915401.500	34115231.390	33303696.305	52687884.268
(3S)-3-hydroxycyclocitral	8039748.930	14434012.210	20880604.139	7568816.262	7542420.511	9582978.347	36270621.598	8005356.303	56451357.416
Thymidine-5'-monophosphoric acid	351824.014	159144.462	122477.359	1611725.852	1392421.089	2209763.650	2668951.576	9516772.729	5375608.074
4-Hydroxy-3-methylbenzaldehyde	932656.108	2042838.824	1788146.997	2725028.027	2949836.041	4178719.918	3609717.649	1303308.846	1509641.296
5-methylthiopentanaldoxime	2149161.239	2389587.750	3117056.053	35943620.252	38878081.779	48407015.619	12264022.978	10716128.289	17350264.542
AICAR	2273710.481	4219068.605	4202533.811	7530548.826	9018127.438	10110216.340	4661605.342	3915171.305	6785186.289
Noreugenin	1998634.918	4230144.533	4206365.648	11231625.730	12051562.305	17268186.764	3959702.566	3350951.824	5695667.510
Butyl propionate	13196013.667	24930876.579	21211338.170	4364793.894	5508429.712	5494059.447	6041601.095	5879604.558	9284714.278
Anhydroglycinol	943440.115	668944.543	1892945.966	13245208.577	10049679.600	14422449.466	1461119.084	1148951.460	1759809.765
10beta-hydroxytaxa-4(20),11-dien-5alpha-yl acetate	91024227.002	169248488.762	161253649.574	102089631.353	108092099.700	134403808.834	174717703.250	170564422.905	285074326.401

name	S1	S2	S3	Y1	Y2	Y3	Q1	Q2	Q3
Diacetyl	10961470.444	20892146.175	19646190.716	7194572.361	7862112.365	9882283.767	25352431.772	26599849.418	40953581.140
7-hydroxy-4-isopropenyl-7-methyloxepan-2-one	2415574.956	6285209.142	6097633.642	5351205.247	3592856.117	4365556.227	6480897.011	6133860.888	17764828.245
Homofureanol	13328750.791	25809825.728	23951666.929	7684820.090	9055007.368	10932609.141	24324131.167	24934260.156	42913811.820
Galactitol 1-phosphate	3757836.110	7581241.237	6072841.881	2352726.078	2469336.801	3179109.531	631295.414	530805.778	1063508.583
2,3,5-Trihydroxytoluene	21931017.992	42712439.836	37293136.903	9602102.388	9492647.852	11917818.322	69676636.464	72388679.142	113850259.603
Metanephrine	14388917.301	29288843.758	2879439.888	4828003.966	444248.922	4151828.223	2099211.441	2151294.320	3090950.026
Mandelonitrile	3691238.535	6724774.650	5999609.223	5825809.661	5467104.647	7608719.118	4023268.872	3714661.305	6335098.882
Mercuron	224600589.322	413385582.443	357162500.892	21510583.250	24478505.794	27638046.106	50209919.690	54134065.703	90357885.273
methyl gibberellin A9	25320527.129	41692583.808	45762483.728	84531570.968	38565135.094	46627076.280	38167143.372	48276668.188	65866353.408
castanin	4514222.089	20217387.771	9186404.656	6084496.547	28709426.775	19803814.482	5677315.529	8296253.212	13879134.009
L-Glutamic acid 5-phosphate	436035.039	763159.203	669449.000	335495.503	438531.067	780794.953	1433651.504	1222525.563	1845275.194
Tetramethylpyrazine	9913861.792	18722884.463	15829137.452	4839751.682	4507765.394	5921330.708	9006565.962	7742432.061	14236948.040
Tabtoxinine-delta-lactam	6774475.707	7922307.993	7667754.182	42471347.447	1818285.266	64357299.727	11409988.086	8295257.712	18965884.841
N-Isopropylammelide	13513767.104	28559432.991	24364215.445	1452749.398	1547947.489	2324701.976	37095819.216	39412126.882	59590024.650
Umbelliferone	1294559.801	1978237.159	1617486.076	5687074.389	6187000.034	810603.064	12555008.147	1601595.188	19195401.918
P 518	1781422.055	324533.168	269691.321	963189.956	1240386.112	192461.891	1262321.804	1007969.709	2596949.514
Phenylacetaldehyde	1408002.636	2713780.861	2428328.701	630179.870	793964.117	753774.052	1338552.429	1604137.211	2249060.515
Homoserine, O-succinyl-	24789246.308	46311274.059	43851101.159	5459270.489	6810159.635	8622238.518	34383778.679	31687597.284	50309994.040
Tartronate semialdehyde	10403567.342	8651793.336	11186821.288	9931280.107	8511715.235	3539722.130	12873563.102	11139301.553	13249983.529
Chrysophanol	749949.466	2978557.321	1561459.047	15084460.818	12136148.890	21665531.506	1782963.072	1526017.813	2028573.588
Vestitol	31041233.518	19800578.222	56184920.922	41028950.193	8477388.454	70482399.060	49741390.154	148341865.764	83217768.360
Artemetin	55896755.720	62481545.128	21517596.288	23512321.589	24438241.456	29068441.030	51798758.328	76255100.786	230789866.687
10-Apo-beta-carotenal	4387668.841	11403049.888	10721061.754	11480915.071	10273901.072	12624402.759	11625559.078	4572158.323	22841903.103
7-Dehydrodesmosterol	1812943.601	3726974.242	3236315.976	29930678.158	9010297.962	20362647.999	311718.943	237428.002	399156.510
phlorisobutyrophenone	9143759.841	17603185.373	16409068.181	1383423.881	1926669.955	2499148.318	4570456.142	4882419.319	6338020.008
Menadiol	237955.765	551977.251	396652.036	165341.795	266818.509	298597.781	298565.708	165778.795	520973.116
cis,cis-3-Carboxymuconic acid	654298.782	1058101.925	1179710.744	847101.053	993732.814	1021076.865	562332.850	778166.865	766428.152
3-Dehydroecdysone	8415899.898	16144818.725	15060592.010	1048219.199	1377647.219	1419047.093	10035174.254	10500265.515	18581124.346
(-)-Pinoresinol	198024	118181	200560	685824	616382	354438	138917.	41649.9	17477.6

name	S1	S2	S3	Y1	Y2	Y3	Q1	Q2	Q3
	8.292	8.764	6.351	5.220	6.197	4.031	798	61	39
Hydroxyhydroquinone	16378048.698	27939238.744	26870523.348	13162790.717	14027337.886	17493600.242	11861811.404	14129862.051	21684648.711
Benzyl alcohol	938102.771	1403544.531	1691178.766	1008433.592	1052602.849	1420659.208	380956.188	517822.787	1198631.417
N-demethylnarwedine	16062498.243	62143347.038	26486501.039	10041621.495	10764051.758	47870590.070	98013404.220	75227948.926	90320710.675
(4aR,10bS)-Noroxomaritidine	54974746.445	40913312.752	81164284.964	47997766.661	16735993.957	122992021.437	103595805.572	100788764.955	48799126.890
Caranine	160825592.749	34304201.339	107832339.818	27312585.046	13289511.905	80084274.025	46296464.273	92098026.697	42798880.593
PC-M6	9730512.493	1357188.888	2450762.750	28842331.977	27048348.310	229890.229	6446741.742	2862534.790	3332889.363
Catechol	7225198.058	13855977.531	13829395.392	1947986.866	1519606.428	2126839.564	6023054.508	6648393.311	12204143.947
4-Methylcatechol	14350230.996	22288159.713	19493622.747	4052100.062	3808500.546	4737848.949	9047201.899	7199285.947	15413710.669
Urolithin B	2592919.249	4739959.618	3747184.892	2398518.561	1579688.441	2895205.893	3288259.872	1217278.966	5534807.682
stylopine	719397.894	1329407.660	1295981.539	5523516.285	7092577.062	7513916.703	1298862.168	1223158.889	1077327.103
noroxopluviine	68768355.432	367084800.797	273059992.785	19301291.378	77159736.574	43180837.620	27865247.843	25442763.909	149277073.865
Sulfate	93588822.139	195170340.204	174030111.169	64386462.885	68322030.827	85496515.117	89098051.568	88722438.273	148570340.338
Imidazole-4-acetaldehyde	924888.562	5056530.110	1277940.419	622080.046	375290.212	528997.226	3029661.892	5309643.416	3693518.624
Premithramycin A2	2300411.781	4054778.000	7271051.071	5678052.953	7147518.267	7511102.784	344853.883	800938.696	1412467.905
5-(Hydroxymethyl)-2-methylpyrimidin-4-OL	34251.924	772735.573	753212.235	2047798.709	1782876.562	805167.578	1260166.384	2119480.482	2150701.951
Nitrilotriacetic acid	450849.925	6418710.437	584004.729	2125412.103	2235953.017	2657896.288	768015.503	1071044.428	1810599.903
gamma-Glutamyl-gamma-aminobutyraldehyde	4600168.384	5315148.178	2484192.836	77385669.296	85283469.961	114613139.983	10823192.146	8380912.268	14039533.832
γ-Aminobutyric acid	29747925.207	56091587.424	52110625.456	10734469.488	10776097.905	15296582.740	55610927.351	59738224.199	96904291.967
5-Hydroxyconiferaldehyde	2361805.768	8215651.809	12565179.015	7863492.688	7982717.866	2953216.623	3866265.981	2265952.877	23753536.127
Secophenol	1200989.205	2240724.034	1619862.732	8814582.669	11980813.484	15496000.832	3102818.774	11218984.731	10386096.967
Propyl acetate	1155212.735	6564473.442	7246752.646	8329918.459	8211690.476	10688670.637	14360285.479	10344314.768	15152574.291
3,7-Dimethyluric acid	6944038.090	13658703.451	11587717.010	3018629.493	2865926.963	4056021.198	9022597.917	9744523.912	13918637.617
14,15-Epoxyemindole SB	489583.971	2856334.880	1849691.949	2116663.504	3664298.759	4489795.651	5365087.264	3076494.723	4630978.995
3-Hexanone	12306941.426	22584895.137	19648718.778	8117550.544	6045496.126	7474143.622	6029147.397	1452403.055	7815953.077
ent-cassa-12,15-dien-2-one	318928.144	689195.581	653768.048	405566.573	368197.356	519199.595	438193.367	426194.939	821739.811
Naringin	3732650.918	5543021.433	5696796.814	3702802.333	4376973.270	7226497.265	2108475.165	1531174.427	3804017.932
Isopentenyl pyrophosphate	289399.501	950153.326	963018.660	58779.430	58081.808	111980.707	967538.681	538079.061	1255851.479
SCHEMBL14765177	2063618.046	3461888.727	3454505.433	46293.263	65989.232	375611.940	1614533.259	1593605.751	2699175.693

name	S1	S2	S3	Y1	Y2	Y3	Q1	Q2	Q3
N-Acetyl-b-glucosaminylamine	4801925.188	7906188.035	7495678.520	244516553.316	263106465.720	334374743.224	7394955.625	7216631.164	12136078.002
aminopyrrolnitrin	27929661.002	52085186.500	44343005.174	26649927.498	26254794.018	31246506.134	42825949.933	41509050.615	61819616.292
1,3-Benzenediol	5086467.134	10547038.596	8753510.604	3037364.794	3279770.721	4077743.315	6874431.642	7023082.160	10608343.139
6-hydroxy-2-cyclohexen-one-carboxylate	2648696.925	5398102.063	5138674.846	450038.639	619329.443	801567.522	2663257.597	2769966.108	3679487.029
2-Vinyl-4H-1,3-dithiine	14504776.758	26591587.058	24251442.037	108727537.006	113073503.657	146223372.518	117794206.466	121823049.659	203299455.894
5'-Deoxyadenosine	1968410.905	522970.172	3014861.058	1365512.531	1653648.473	1650788.743	3906210.135	6637893.231	9813719.187
6,7-Dimethyl-8-(1-D-ribityl)lumazine	2591269.923	4779264.820	4786999.452	25281388.091	23912375.990	31567390.638	7316333.328	7932499.617	12492856.940
Aniline	4642955.952	9132856.528	7676828.098	2737785.852	2981876.925	3459318.611	9484637.309	10318058.813	15295817.256
Oryzalexin E	13891335.310	25672852.994	24241870.237	6384511.430	6398394.896	9118950.175	13931861.274	14974061.836	21470103.781
3-Indoleacetonitrile	940942.573	1208650.826	1370278.455	10749743.681	10481640.518	14596097.093	1160676.742	901467.595	1429256.494
S-adenosylhomocysteine (SAH)	855012.126	1887467.780	1470032.438	8145111.786	8364871.295	8086875.174	4468800.281	4186127.695	7877760.274
Cyanuric acid	2823612.120	8979515.082	5731806.393	3627360.706	3812018.760	4156805.802	7112406.530	7459646.442	9543256.229
aurachin B epoxide	48589145.523	88165049.812	75791373.253	4577428.277	4376036.030	4808402.125	35708362.655	41482985.890	67924529.165
4-hydroxy-2-nonenal (4-HNE)	2375066.104	4508374.412	3563180.953	299250.205	306546.452	390556.633	4285940.887	4061157.502	6843081.244
Sinapaldehyde	1760281.159	3194003.458	5852428.211	19071196.445	19536758.087	1776476.319	4333036.106	4387007.777	6863748.813
Thiamine	485930.745	1093133.897	1045943.122	2269756.359	728818.123	776019.617	4749756.815	1185201.557	2377107.128
chloric acid	587949072.275	1712820673.267	1466945376.372	663497526.693	807434804.006	1343671430.594	1138290902.635	944454989.461	1922652023.395
10-Hydroxydihydrosanguinarine	44167126.663	86208419.163	82104158.502	332612949.973	497727894.609	1170235788.855	8445295.069	8184212.002	15917922.757
taxa-4(20),11-dien-5alpha,13alpha-diol	29791050.607	56144296.020	49212628.233	238343.645	228644.588	260132.951	13194202.653	13641588.493	20799653.012
Coniferin	29410643.898	52057035.373	46853168.051	73511015.932	71486608.194	162466061.799	72865860.134	45206900.485	74695889.250
Tert-butyl formate	13781831.667	26227541.404	24737677.431	20780899.521	19243379.590	23317305.741	50318653.173	53347248.505	89353297.518
Isoandrocymbine	3941191.991	12348688.370	3954480.851	2712014.926	1473733.111	2431615.463	1421122.308	1425484.801	1855376.723
2-methyl-1,5-dinitro-3-nitrosobenzene	2565124.708	1200447.116	3745691.693	20203.449	190265.167	1782703.506	49495148.242	48225162.550	84105559.944
8-demethyl-8-(2,3-O-dimethyl-alpha-L-rhamnosyl)tetracenomycin C	2464708.635	3370054.319	2630884.394	50218416.022	48593524.821	59863021.292	12017796.717	10974063.170	15836676.454
Ergosta-5,7,22,24(28)-tetraen-3beta-ol	64440887.747	164605941.711	142615974.726	22782027.462	23903234.872	26619599.242	63909746.489	27690580.060	132484816.985
Dihydrobiopterin	1270607.856	2459186.693	2167894.607	192497.118	157584.627	207267.597	6234984.433	6440160.103	9446394.197
ascopyrone M	3616768.449	5738377.467	5890732.502	2394695.690	2309066.968	3144361.376	4474243.946	4751668.327	6101739.318
3,4-dihydroxy-2-methyl-4-farnesyl-3H-quinolin-1-ium	471536	278405	228924	717393	870982	100926	164741	544923	308956

name	S1	S2	S3	Y1	Y2	Y3	Q1	Q2	Q3
-1-olate	98.856	081.501	969.916	20.119	03.885	444.288	751.021	59.391	391.879
Toluene-cis-dihydrodiol	2280054.285	4456927.988	4123380.730	699620.223	642511.823	718506.865	1492471.364	1580175.425	1739310.755
D-5-O-Methyl-2,3,5_4,6-pentahydroxycyclohexanone	72345305.894	136492643.327	125650103.857	366708922.666	405265666.998	541464760.579	372844935.138	415599401.475	618664521.103
2-cis,4-trans-xanthoxin	5663699.779	10738802.106	13715662.525	21944314.670	9389268.975	6664951.225	6972117.234	9272740.531	12494436.870
Coumesterol	9045051.901	16439669.903	11120657.885	9360830.163	14234093.460	18273308.815	7465735.699	6730713.012	11380354.288
Chavicol	698244.128	1260733.051	942948.619	1756118.839	894037.355	781461.912	1325966.882	1326961.504	1855144.203
N-Acetylmannosamine	849806.169	1523879.978	1726485.469	97923.981	103926.753	652244.422	482300.834	143307.592	1153376.461
Aurachin B	14720587.081	29686088.362	26925904.696	2694396.306	2438812.318	4168348.748	7871707.097	6912369.075	10884429.844
Pseudomonine	49759.273	130663.466	1972668.894	3403871.958	2669489.307	12166017.393	1326482.783	1438809.432	2902268.252
5-hydroxyindole thiazolidine carboxylate	298204800.405	555422660.382	411666996.407	881681593.961	786802266.767	1128283161.510	79176649.045	85354721.822	122352818.620
1H-Indole-2,3-dione	4091840.342	10186457.050	9180295.049	3974914.481	6880511.341	4113789.375	8269648.266	6061202.409	11760058.604
Urolithin A	38442934.463	51191878.589	59691028.875	8553555.598	10353380.861	21243938.406	997526.169	882628.815	1514178.620
Cirsimaritin	9954522.553	19859605.797	14190493.479	49471110.374	48800469.830	54060531.143	1288443.042	543064.882	596353.271
D-proline betaine	115582.755	193498.920	130991.923	166876.091	193080.747	71218.037	486116.754	471494.737	542996.443
Cathasterone	6587211.978	46289249.793	12931623.359	19679525.026	8641348.463	5718516.182	56439320.134	16851178.222	13488140.608
Zeatin	4342176.719	8214804.179	7416646.838	1966303.389	1822956.943	2462295.711	5601816.802	5630842.882	8723704.627
4,4-dihydroxy-alpha-methylstilbene	989847.556	1925918.285	1746174.950	1357215.623	1384248.940	1835383.946	17249248.820	2037891.987	3648979.539
Ginsenoside F1	1211730.555	3165282.638	72705132.264	48900093.453	57589891.137	50681223.908	363658854.475	198401204.197	74574150.451
glyceollidin I	397625.972	1129728.993	174611.477	14628356.374	15877307.238	17879384.333	3624848.093	4002518.136	5979200.778
Shisonin	11680090.451	23897964.541	19679966.252	16797254.189	25361727.686	22220804.730	9070537.186	9735099.676	23291104.243
Pyridoxal 5'-phosphate	15468937.712	7843481.282	28835873.203	28422789.983	5607281.781	27639206.283	1471414.116	645811.879	983217.786
betaine aldehyde hydrate	26189335.235	49365487.927	43863033.771	1171403.542	819184.852	1024511.296	2413498.608	2274151.823	3369876.335
Griseophenone C	724766.155	1214070.887	1385203.283	829121.885	3586575.623	1135483.308	19125233.015	18238986.710	30164686.328
D-Tagatose 6-phosphate	1575929.395	3450732.383	5623007.156	2788521.498	1263435.782	2985026.011	22789465.438	31526567.848	43122931.147
Daidzein	8733045.543	3258375.811	7743734.521	9022072.831	7376195.581	2733431.765	12895514.040	2887328.909	11286266.061

Table S2. Differentially Expressed Metabolites in Pu-erh Tea from Different Years

	name	mz	rt	ppm	formula	S_Mean	Y_Mean
1	2,4-DINITROANISOLE	155.0455	72.8	2.470112967	C7H6N2O5	10997780.07	55037479.47
2	Glycylvaline	173.0917	55	8.44	C7H14N2O3	3796510.97	616331669.4
3	4-Methoxy-2,2-bipyrrole-5-carboxaldehyde	174.0554	73.3	2.577224837	C10H10N2O2	36897530.31	3021514.31
4	Portuloside A	331.2485	282.2	1.848	C16H26O7	152815829.7	2784514.64
5	Sinapine	349.258	237.8	14.86242291	[C16H24NO5]+	167363132	753588.92
6	Prolyl-Alanine	169.0967	88.4	2.654	C8H14N2O3	48607009.76	131918751.2
7	ST 21_2;O3	331.233	235.6	15.531	C21H32O3	940222237	1426781.96
8	Isopropylmaleic acid	181.05	123.4	15.87	C7H10O4	4357436.35	329098656.7
9	1,6-di-O-Galloylglucose	502.1178	158.8	2.643642787	C20H20O14	43279671.23	834714.72
10	4-Methoxy-2,2-bipyrrole-5-carboxaldehyde	174.0554	73.3	2.577224837	C10H10N2O2	36897530.31	7320037.77
11	Dihydro-3-(1-octenyl)-2,5-furandione	211.1332	217.3	1.59	C12H18O3	5818790.93	47150942.61
12	Sucrose	360.1506	49.3	1.578	C12H22O11	508986954.3	18208760.78
13	1,3,5-Trimethoxybenzene	337.1616	54.2	8.769741572	C9H12O3	637137405.2	444006.15
14	Harmine	235.0807	95.9	14.79874826	C13H12N2O	1394321.41	54559789.17
15	Neochlorogenic acid	355.1026	124.5	0.692	C16H18O9	386788576.3	7858979.41
